# Human Metapneumovirus Nucleocapsid Inhibitors Discovery for Targeting Viral Replication and Genome Encapsidation: An In Silico Approach

**DOI:** 10.1155/jotm/3511825

**Published:** 2026-05-20

**Authors:** Abdullah R. Alzahrani, Talha Jawaid, Maha M. Bakhuraysah, Amani A. Alrehaili, Hayaa M. Alhuthali, Abdullah Yahya Abdullah Alzahrani, Zia Ur Rehman, Abida Khan

**Affiliations:** ^1^ Department of Pharmacology and Toxicology, Faculty of Medicine, Umm Al-Qura University, Al-Abidiyah P. O. Box 13578, Makkah, 21955, Saudi Arabia, uqu.edu.sa; ^2^ Department of Pharmacology, College of Medicine, Imam Mohammad Ibn Saud Islamic University (IMSIU), Riyadh, Saudi Arabia, imamu.edu.sa; ^3^ Department of Clinical Laboratory Sciences, College of Applied Medical Sciences, Taif University, P. O. Box 11099, Taif, 21944, Saudi Arabia, tu.edu.sa; ^4^ Department of Chemistry, Faculty of Science, King Khalid University, Abha, 61413, Saudi Arabia, kku.edu.sa; ^5^ Health Research Centre, Jazan University, P. O. Box 114, Jazan, 45142, Saudi Arabia, jazanu.edu.sa; ^6^ Department of Pharmaceutical Chemistry and Pharmacognosy, Faculty of Pharmacy, Jazan University, P. O. Box 114, Jazan, 45142, Saudi Arabia, jazanu.edu.sa; ^7^ Center for Health Research, Northern Border University, Arar, 73213, Saudi Arabia, nbu.edu.sa

**Keywords:** computational drug discovery, human metapneumovirus, molecular dynamics simulation, nucleocapsid

## Abstract

Human metapneumovirus (HMPV) is a major respiratory pathogen, and its infection is more severe in infants, the elderly, and immunocompromised individuals. Despite its significance, no antiviral drugs are approved to combat HMPV infection. Therefore, it is important to develop drugs that target the nucleocapsid protein, which is crucial for viral replication and genome encapsidation. In this study, computational drug discovery was used to identify new potential molecules targeting the HMPV nucleocapsid protein. In the first step, the virtual screening method was applied to a compound library, and the top three compounds were selected for further redocking using AutoDock Vina. Finally, the chosen compounds were subjected to molecular dynamics simulations, and the stability of the complex and its conformational stability were evaluated using root mean square deviation, root mean square fluctuation, principal component analysis, and binding free energy landscape analysis. Among the chosen compounds, 24,330,502, 24,292,974, and 17,515,455 showed high binding affinity, stability, and conformational stability, and their results were better than those of the reference compound, Gamma‐Fagarine, and they retained the ligands in the binding pocket with high stability. Therefore, the results of the present study reveal that the selected compounds, namely, 24,330,502, 24,292,974, and 17,515,455, have the potential to inhibit the HMPV nucleocapsid protein and are thus important for the treatment of HMPV infection.

## 1. Introduction

Human metapneumovirus (HMPV) is one of the leading respiratory viruses causing acute respiratory tract infections among all age groups, from infants to elderly and immunocompromised patients [1–4]. It was discovered in 2001 and belongs to the family Paramyxoviridae, sharing genetic similarity with the respiratory syncytial virus. The infection can range from mild upper respiratory tract symptoms to severe pneumonia and bronchiolitis that require hospitalization in vulnerable populations [5]. This virus is considered one of the major causes of pneumonia in children; as such, it comes second after RSV and influenza among the common causes leading to hospitalizations in pediatric units [4, 6]. HMPV circulates worldwide, causing seasonal outbreaks that peak in late winter and early spring. Seroprevalence studies demonstrate that most children are infected with HMPV by age five; however, reinfections continue to occur throughout life, reflecting partial immunity [7]. Respiratory droplets and fomites spread it, and it can thus spread rapidly among people in close contact, including in hospitals and nursing homes [8]. Recent epidemiological studies implicate HMPV as causing significant morbidity and mortality, with enhanced disease severity during coinfection [1]. Yet, despite this clinical burden, no licensed vaccines or specific antiviral therapies exist, underscoring an urgent need for targeted therapeutic strategies to blunt the public health impact of HMPV outbreaks [9].

The genome of HMPV has been characterized to contain a single‐stranded, negative‐sense RNA of about 13 kbs coding for nine proteins, including nucleocapsid (N), phosphoprotein (P), matrix (M), fusion protein (F), attachment glycoprotein (G), small hydrophobic (SH), large polymerase protein (L), and two nonstructural proteins (M2‐1, M2‐2) [4, 10, 11]. The latter, represented by the F protein, constitutes the main target of neutralizing antibodies, directly interacting with both cellular entry and membrane fusion; nevertheless, considerable sequence variability and antigenic drift complicate efforts toward universally acting inhibitors [6].

Another promising target of antiviral therapy is a large polymerase (L) protein involved in viral replication and transcription [12, 13]. Inhibition of L polymerase might directly prevent viral genome replication; it generally resembles RSV polymerase inhibitors as a broad‐spectrum antiviral approach. Phosphoprotein P represents an essential cofactor of a viral polymerase complex and, therefore, also a potential drug target for small‐molecule inhibitors [14].

The matrix (M) protein, which regulates viral assembly and budding, is also a protein target. Interference with the function of the M protein could be one way to prevent virion maturation and release, reducing virus spread. Indeed, many of these structural and nonstructural proteins are potential therapeutic targets, among which the nucleocapsid (N) protein may be an attractive drug target because it is highly conserved and essential for viral replication [15, 16].

The nucleocapsid (N) protein of HMPV plays a pivotal role in genome encapsidation, protecting the viral RNA from host immune responses and enabling efficient transcription and replication. The N protein forms a ribonucleoprotein complex with the phosphoprotein (P) and RNA‐dependent RNA polymerase (L), facilitating viral RNA synthesis. Given its highly conserved sequence and indispensable function, the N protein is an attractive target for antiviral drug development [12, 17].

Targeting the N protein could disturb the viral replication cycle by inhibiting its interactions either with RNA or the polymerase complex [18]. Various viruses, such as RSV and measles, have already demonstrated that nucleocapsid inhibitors are effective at impeding viral propagation. Small‐molecule inhibitors against the HMPV N protein can represent a new therapeutic approach to decrease viral loads and disease severity [19–21]. Computational drug discovery offers a promising avenue for efficiently identifying such inhibitors.

Various strategies are being used to combat HMPV infection in the absence of vaccines and FDA‐approved antiviral therapies. Antiviral drug development is one of the mainstays, targeting viral proteins such as N, F, and L with small‐molecule inhibitors [22]. The broad‐spectrum antivirals, including ribavirin, have shown limited efficacy so far; therefore, more specific inhibitors are needed. Monoclonal antibodies against the fusion protein F are also under study, but high production costs and antigenic variability make this approach challenging [23].

Computational drug discovery has transformed antiviral research by enabling the rapid identification of lead compounds [12, 24, 25]. In silico methods, including molecular docking, virtual screening, and molecular dynamics (MD) simulations, enable screening large drug‐like libraries against viral targets. These approaches significantly reduce the time and cost associated with traditional drug discovery. Utilizing these computational techniques, it is possible to identify and optimize potential inhibitors for the nucleocapsid protein of HMPV for further experimental validation [22].

In the present investigation, in silico drug design techniques were employed to select molecules that could act as potential inhibitors of the HMPV N protein. Interfering with the functions of such an important protein involved in viral replication might offer an effective way of antiviral therapy. Herein, a combination of virtual screening of a library of drug‐like compounds, molecular docking, and dynamics was applied to predict putative inhibitors. This will therefore be a stepping stone toward future experiments and anti‐HMPV development.

## 2. Methodology

### 2.1. Protein Preparation and Active Site Prediction

This 3D structure of HMPV nucleocapsid N protein with PDB ID: 5FVD was selected due to its high resolution (1.86 Å), structural completeness, and biological relevance as an RNA‐free, monomeric N protein bound to the P cofactor [26–28]. For this protein, the DockPrep module in UCSF Chimera was used to prepare the structure [28]. The crystallographic water molecules were removed to ensure the integrity of the structure. Hydrogen atoms were added to complete the protein and thus optimize it for docking. Key residues in and around the predicted binding site were manually inspected to ensure proper protonation, and no adjustments were necessary. The overall structure was minimized to relieve steric clashes. Afterward, the prepared protein was saved in a PDB format for analysis.

The potential binding sites of the N protein of HMPV were predicted using the CASTp web server. To increase biological accuracy, the largest pocket predicted by CASTp was manually validated by checking existing protein‐binding sites [29]. Notice that certain residues of this pocket (e.g., Leu167, Trp261, Ile334, and Met337) are involved in RNA‐binding sites or N‐protein oligomerization, an important step for N‐protein function. The results validated the choice of protein binding sites to perform molecular docking studies. The geometric center of this site was determined, and the corresponding coordinates were extracted for virtual screening.

### 2.2. Virtual Screening and Redocking

A diverse set of small‐molecule inhibitors was screened using the MTiOpenScreen web server [30]. The diverse‐lib compound library available on the web server was screened against the predicted active site of the N protein [30]. This library was chosen for its moderate size and chemical diversity, with prefilters for drug likeness, including Lipinski’s “rule of five.” Furthermore, the diverse‐lib library has been used in antiviral discovery research and is optimized for structure‐based virtual screening. The library’s accessibility as an academic, web‐based platform for virtual screening using MTiOpenScreen is beneficial for screening within current computational resource limits. The workflow in this screening involves filtering based on molecular docking scores, drug‐likeness properties. Top‐ranked compounds with higher binding affinity were shortlisted for further analysis [31].

The top three compounds identified in the virtual screening study were further tested for their ability to redock into the receptor’s binding site using the AutoDock Vina plugin in UCSF Chimera [28, 32]. The grid was set to a defined size and positioned at the binding pocket of the receptor, using CASTp coordinate information. As a comparison, a standard compound (Gamma‐Fagarine) was also used, following the same docking protocol. The binding energy of the docked compounds was used to assess their order. Interactions between the receptor and ligands were determined utilizing the Desmond/Maestro visualization module to reveal critical interactions such as hydrogen bonds, hydrophobic interactions, *π*‐*π* stacking, and salt bridges [33, 34]. In the current study, Gamma‐Fagarine was selected as the standard compound based on its antiviral relevance, favorable physicochemical properties, and its ability to interact stably with the identified binding pocket of the HMPV nucleocapsid (N) protein in the preliminary screening.

### 2.3. MD Simulation

MD simulations were carried out with the freely available academic Desmond/Maestro package [35–38] to study the stability and dynamics of the protein–ligand complex [34–37]. The parameters were set to ensure the desired accuracy in the simulations, using the OPLS‐2005 force field [39] to describe the interactions between the atoms in the system. The complex was solvated in an orthorhombic periodic box with the TIP3P water potential [38] to ensure a good description of the water molecules in the system, with a minimum distance of 10 Å between the solute and the box boundaries to avoid periodic boundary effects. The system was then neutralized with the addition of counter ions (Na^+^ or Cl^−^) and 0.15 M NaCl to mimic physiological ionic strength.

Energy minimization was then carried out to remove steric clashes between the atoms in the system, with the steepest descent and conjugate gradient methods used to an energy threshold of 1 kcal/mol/A [39]. The system was then equilibrated in two stages: the first was NVT equilibration, where the temperature was gradually increased to 300 K over 200 ps with the Berendsen thermostat, with position restraint applied to the protein and ligand heavy atoms [40]. The second was NPT equilibration at 1 atm for 500 ps with the Nosé–Hoover thermostat and the Martyna–Tobias–Klein barostat to ensure the stability of the system [41].

Finally, a 500 ns MD trajectory was run in the NPT ensemble with a 2‐fs time step, with the trajectory saved every 10 ps for subsequent analysis. The electrostatic interactions were handled with the PME method, with the van der Waals interactions truncated at 10 Å with a smooth cutoff function [42]. The postsimulation analysis was carried out with Desmond’s simulation interaction diagram (SID) tool.

To ensure the accuracy and reliability of the results, the convergence of the trajectory was checked over the 500 ns period. The trajectory was considered to have converged if the RMSD values plateaus, meaning that the values no longer change with time. Only such stable trajectories were considered for the analysis.

### 2.4. Principal Component Analysis (PCA)

PCA was used with the Bio3D package for R to investigate the conformational space occupied by protein–ligand complex [43–45]. Bio3D was used for these purposes since its ability to handle MD simulations has already been demonstrated, including calculation of a covariance matrix, eigenvector decomposition, and visualization of significant movements for the understanding of protein dynamics. The covariance matrix for atomic fluctuations was calculated and used for eigenvector decomposition to highlight significant movements.

### 2.5. Free Energy Landscape (FEL) Analysis

The Geo‐Measures PyMol plugin created a FEL using the mapping of PC1 and PC2 to identify the lowest energy states. The FEL provided insight into the most stable states of conformations sampled in the MD simulation [46–48]. The minima structures of the lowest energy states were obtained for further comparison.

### 2.6. Minima Structure Extraction and Superimposition

For the determination of the most stable structural conformations, the minima of the FEL analysis were extracted and superimposed on the initial docking poses using the UCSF Chimera software. This allowed the assessment of the ligand‐induced conformational changes and the stability of the identified inhibitors.

### 2.7. MMGBSA Analysis

Binding free energy calculations were performed using the Prime MM/GBSA method in the Prime Module of Schrödinger’s suite at the Department of Biology, University of Hail, Hail, Saudi Arabia [49]. The calculations were based on data collected during the last 50 nanoseconds of simulation. The OPLS‐2005 force field was applied with a dielectric value of 80, which represents physiological conditions. The energies were calculated for the ligand binding process. The calculation was useful for ranking ligand stability and validating the best‐performing inhibitors.

### 2.8. Physicochemical and Pharmacokinetic Analyses of the Selected Compounds

The physicochemical and pharmacokinetic properties of the selected compounds, including drug‐likeness and medicinal chemistry suitability, were assessed using SwissADME software [50]. The SwissADME software was utilized for performing this analysis [50]. The absorption, distribution, metabolism, and excretion (ADME) properties and drug‐likeness of the compounds were predicted. Moreover, parameters such as the IPinski’s Rule of Five, P‐glycoprotein (P‐gp) substrate prediction, cytochrome P450 (CYP P450) isoenzyme interactions, brain penetration, and gastrointestinal absorption were also evaluated.

## 3. Results

### 3.1. Virtual Screening and Redocking Validation

A virtual screening of revealed 1500 drug‐like compounds against the HMPV nucleocapsid protein revealed a range of binding affinities, varying scores from −10.7 to 8.3 kcal/mol (Table S1). The top three compounds, 24,330,502 (PubChem CID: 1111158; chemical name: 3,4‐dihydroisoquinolin‐2(1H)‐yl[5‐phenyl‐7‐(trifluoromethyl)pyrazolo[1,5‐a]pyrimidin‐3‐yl]methanone) (−10.7 kcal/mol), 24,292,974 (PubChem CID: 4292500; chemical name: N‐(2‐(4‐fluorophenyl)‐4,6‐dihydro‐2H‐thieno[3,4‐c]pyrazol‐3‐yl)‐2‐oxo‐2H‐chromene‐3‐carboxamide) (−10.3 kcal/mol), and 17,515,455 (PubChem CID: 7125864; chemical name: 4‐benzyl‐1‐(4‐fluorophenyl)‐[1,2,4]triazolo[4,3‐a]quinazolin‐5(4H)‐one) (−10.3 kcal/mol), were selected for further computational evaluation due to their superior binding affinities. In order to determine the consistency of these interactions, redocking analysis was performed, and the docking score was found to be −10.2, −10.3, and −9.6 kcal/mol, respectively, which confirmed the stability of the interaction with the target protein (Figure 1).

FIGURE 13D and 2D interaction profiles of the HMPV nucleocapsid protein with selected inhibitors and reference compound. (a, b) Compound 24,330,502, (c, d) 24,292,974, (e, f) 17,515,455, and (g, h) Gamma‐Fagarine are shown docked within the predicted binding pocket of the HMPV N protein. The 3D structures illustrate spatial orientation within the pocket, while the 2D diagrams depict key interactions including hydrogen bonds, hydrophobic contacts, and *π*‐*π* stacking. These visualizations highlight the enhanced interaction networks of the selected inhibitors relative to the reference, suggesting their superior binding potential.

**Figure:**
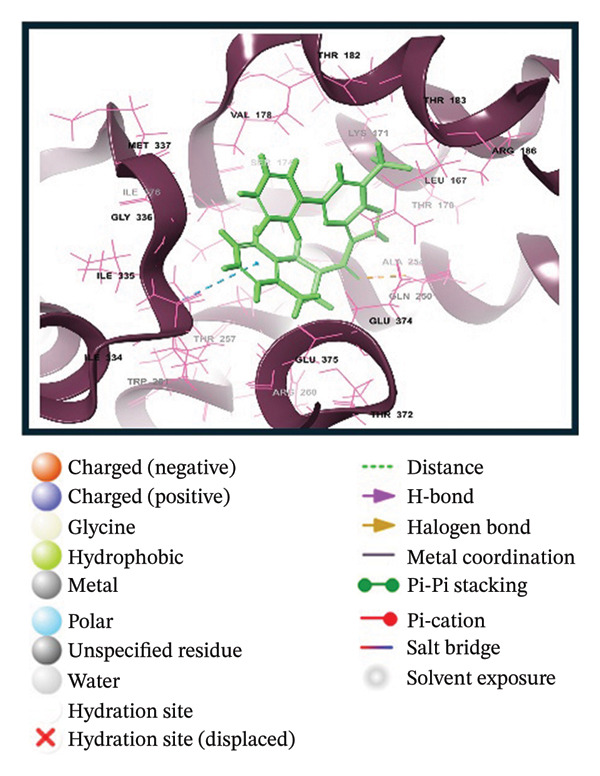
(a)

**Figure:**
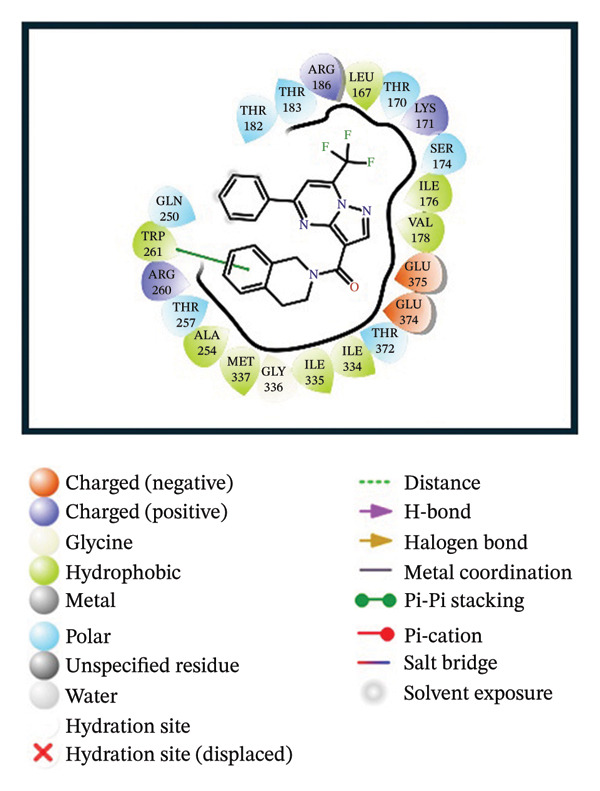
(b)

**Figure:**
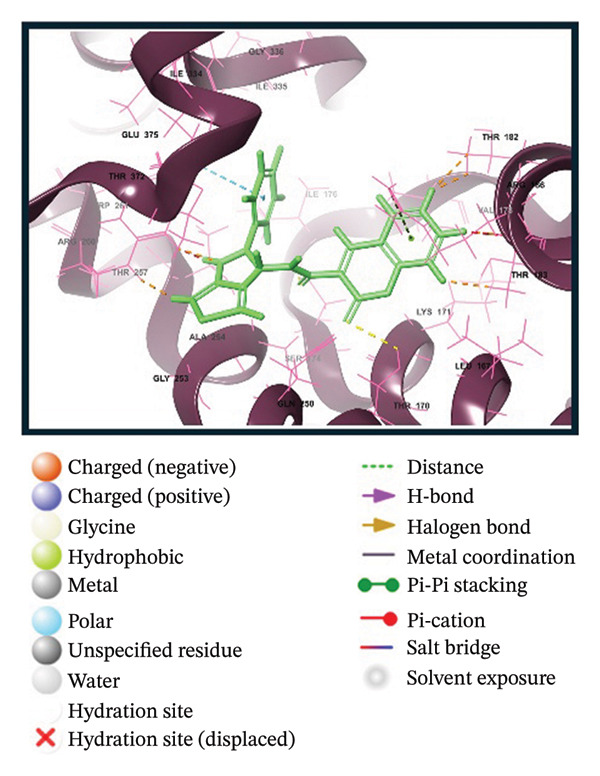
(c)

**Figure:**
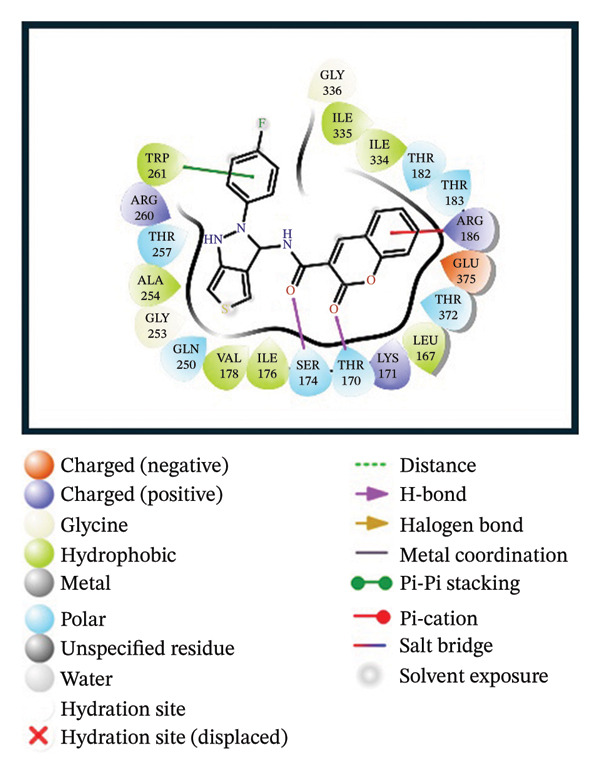
(d)

**Figure:**
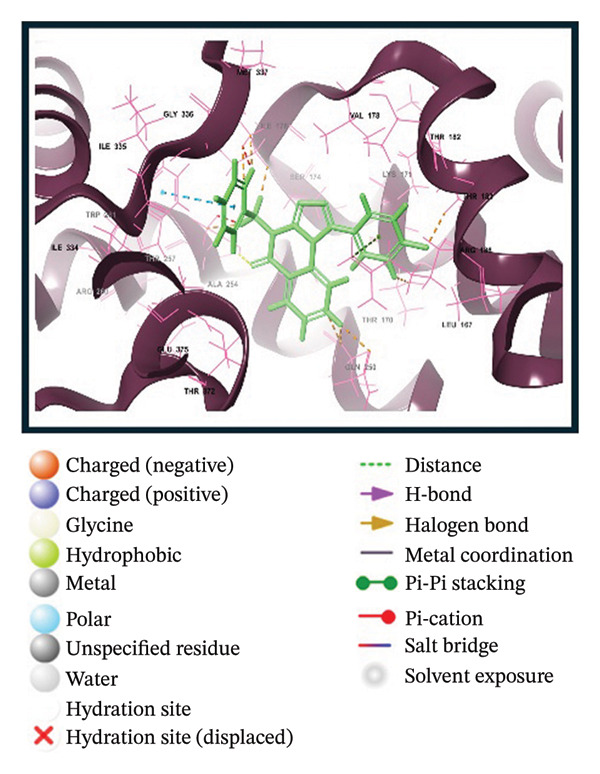
(e)

**Figure:**
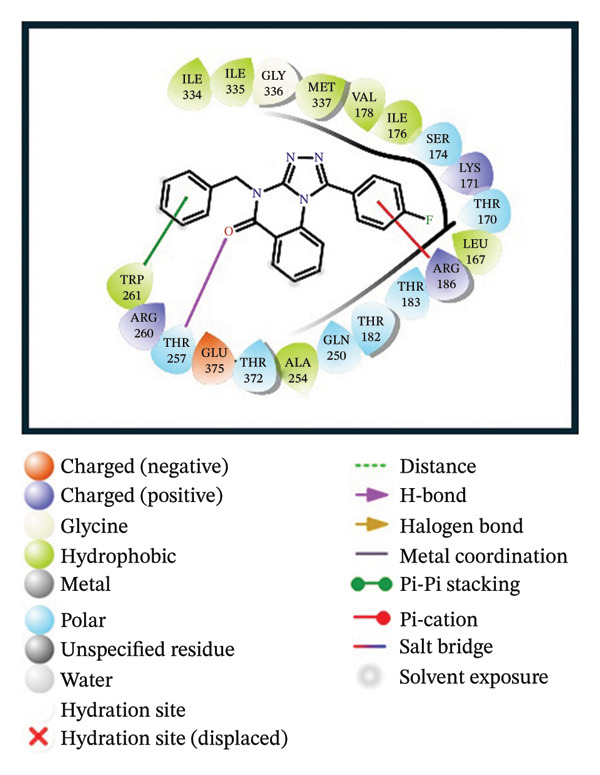
(f)

**Figure:**
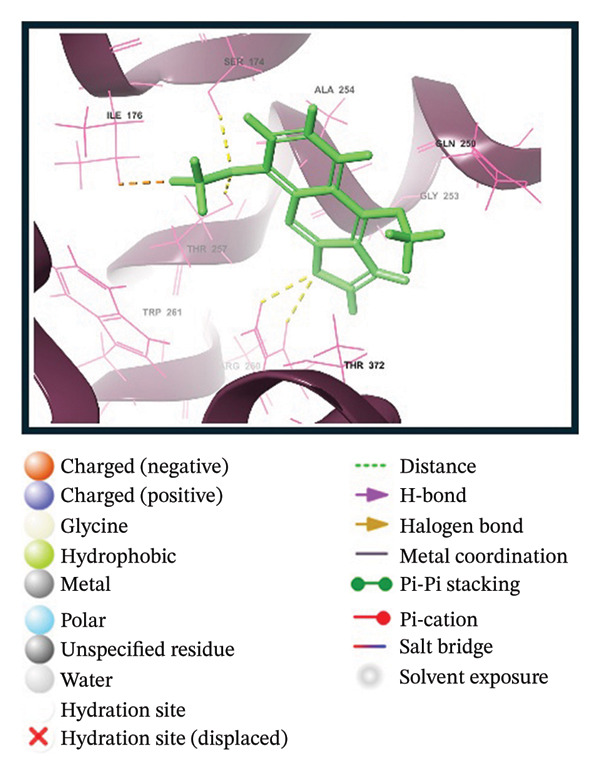
(g)

**Figure:**
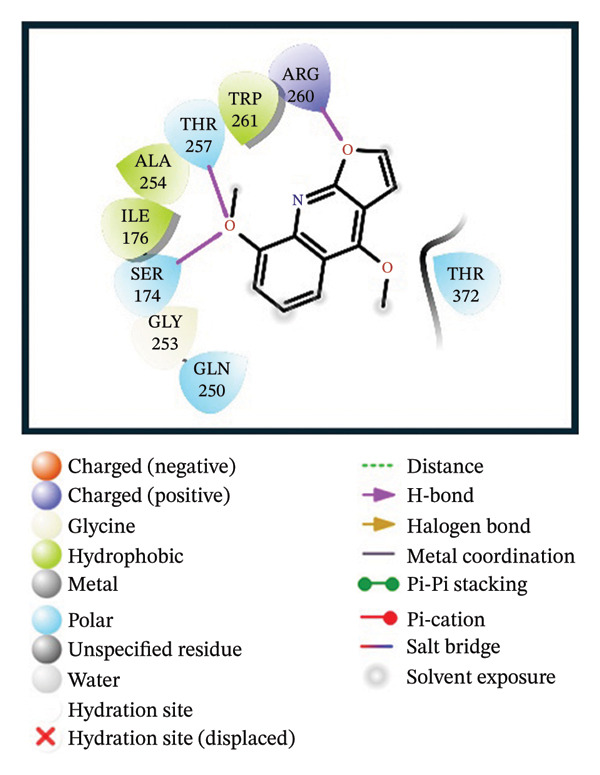
(h)

Redocking analysis was also performed against the compound Gamma‐Fagarine (PubChem CID: 107936), and the docking score was found to be −6.3 kcal/mol, indicating a low level of interaction compared to the compounds of the present investigation; therefore, the latter have a higher interaction potential with the nucleocapsid protein. Therefore, these differences in docking scores suggest that the newly identified inhibitors may be more capable of disrupting protein function and, hence, better candidates for further studies.

Molecular interaction analysis provided additional insight into the binding mechanism. Compound 24,330,502 exhibited an extensive hydrophobic interaction with residues Leu167, Ile176, Val178, Ala254, Trp261, Ile334, Ile335, and Met337, with *π*‐*π* stacking with Trp261. Such an extensive network of noncovalent interactions confers stability in its binding. 24,292,974 formed hydrogen bonds with Thr170 and Ser174 and hydrophobic interactions with Leu167, Ile176, Val178, Ala254, Trp261, Ile334, and Ile335 throughout. Additionally, *π*‐*π* stacking and *π*‐*π* cation‐*π* interactions have occurred with Arg186 and Trp261, adding extra strength.

Compound 17,515,455, on the other hand, exhibits a hydrogen bonding interaction with Thr257 and a hydrophobic interaction with Leu167, Ile176, Val178, Ala254, Trp261, Ile334, Ile335, and Met337. In addition, it is involved in a *π*‐*π* stacking and *π*‐*π* cation interaction with Arg186 and Trp261, respectively. On the contrary, Gamma‐Fagarine demonstrated a limited amount of interactions, which included hydrogen bond formation with Ser174, Thr257, and Arg260 and a few hydrophobic interactions, such as those with Ile176, Ala254, and Trp261, with no *π*‐*π* stacking interactions (Table 1).

**TABLE 1:** The list of residues involved in the intermolecular interaction of each HMPV nucleoprotein docked complex.

S no.	Complex	H‐bond	Hydrophobic	*π*‐*π* stacking/*π*‐*π* cation
1	24,330,502	—	Leu^167^, Ile^176^, Val^178^, Ala^254^, Trp^261^, Ile^334^, Ile^335^, Met^337^	Trp^261^

2	24,292,974	Thr^170^, Ser^174^	Leu^167^, Ile^176^, Val^178^, Ala^254^, Trp^261^, Ile^334^, Ile^335^,	Arg^186^, Trp^261^

3	17,515,455	Thr^257^	Leu^167^, Ile^176^, Val^178^, Ala^254^, Trp^261^, Ile^334^, Ile^335^, Met^337^,	Arg^186^, Trp^261^

4	Gamma‐Fagarine	Ser^174^, Thr^257^Arg^260^	Ile^176^,Ala^254^, Trp^261^	—

Higher docking scores for the selected compounds, compared with Gamma‐Fagarine, and their stronger molecular‐level interactions provide sufficient evidence that they might emerge as effective binders to the HMPV nucleocapsid protein. This calls for further stability analysis using MD simulations, as discussed in the next section.

### 3.2. MD Simulation

In this context, MD simulation was used as an additional investigation tool to offer a deeper assessment of the stability and dynamics of protein–ligand complexes at physiological conditions. In this respect, MD simulation can offer significant information on the ligand stability and protein flexibility at an atomic level, which are considered crucial for assessing the aptitude of a compound. In this direction, this study was designed to carry out a MD simulation for 500 nanoseconds to assess the structural integrity and stability of the three identified inhibitors in relation to the HMPV nucleocapsid protein.

### 3.3. Root Mean Square Deviation (RMSD) Analysis

The RMSD curves obtained for the complexes are representative of the temporal stability associated with the complexes. In the case of complex 24,330,502, low fluctuations were observed, and the RMSD for both the protein and ligand was stable at low values below 4 Å. This indicated a stable conformation in the protein and the ligand within the complexes. In the case of complex 24,292,974, moderate deviation was observed, where the protein RMSD was stable, while the ligand RMSD was fluctuating, reaching a maximum deviation of 12 Å. This could be an indication of flexibility in the protein and ligand, which could be a possible adjustment to enhance the binding process. In the case of complex 17,515,455, stability was observed for a significant period; however, a slight increase in the RMSD was observed at 400 ns, which could be an indication of possible ligand repositioning. In the case of the Gamma‐Fagarine complex, high ligand instability was observed, where the RMSD was above 30 Å, a possible indication of low ligand‐protein binding affinity and possible dissociation from the protein (Figure 2). In all cases, the RMSD curves for the top three complexes showed significant plateaus, an indication that equilibrium was reached within the simulation time period. However, in the case of the Gamma‐Fagarine complex, significant deviation was observed, a possible indication that equilibrium was not reached.

FIGURE 2RMSD analysis of protein–ligand complexes over a 500 ns molecular dynamics simulation. RMSD plots for the HMPV nucleocapsid protein complexed with (a) 24,330,502, (b) 24,292,974, (c) 17,515,455, and (d) Gamma‐fagarine are shown. Stable RMSD values below 4 Å for the selected compounds indicate well‐retained ligand binding conformations throughout the simulation.

**Figure:**
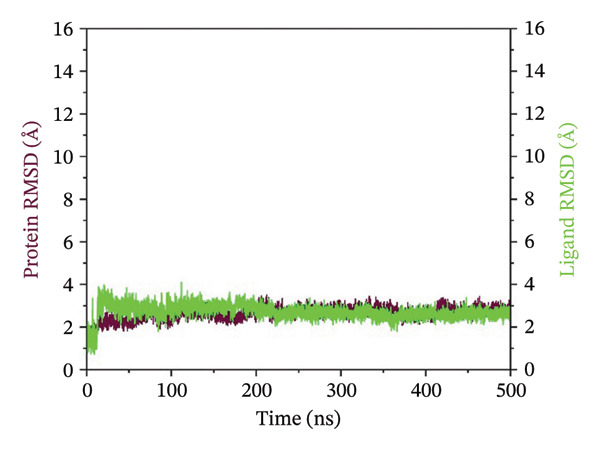
(a)

**Figure:**
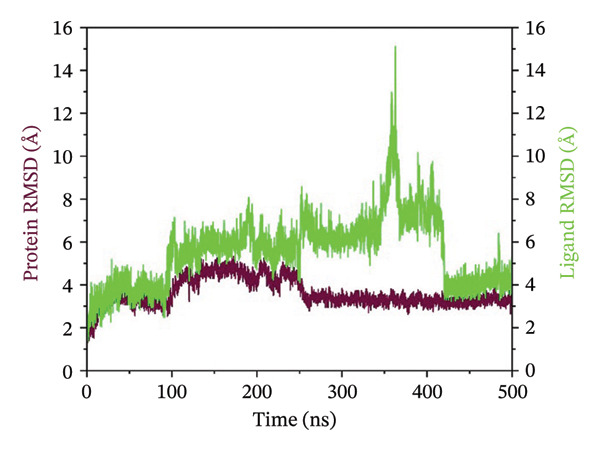
(b)

**Figure:**
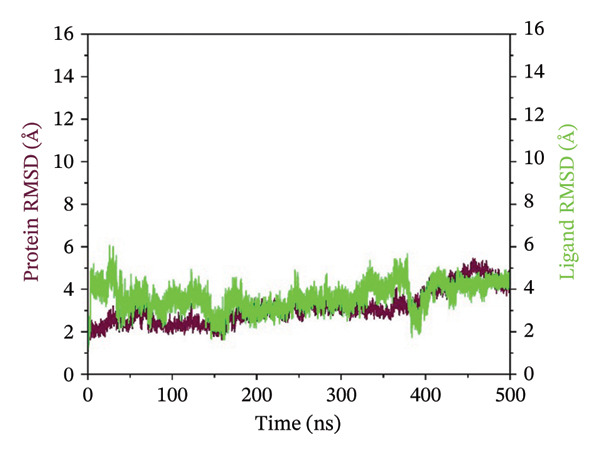
(c)

**Figure:**
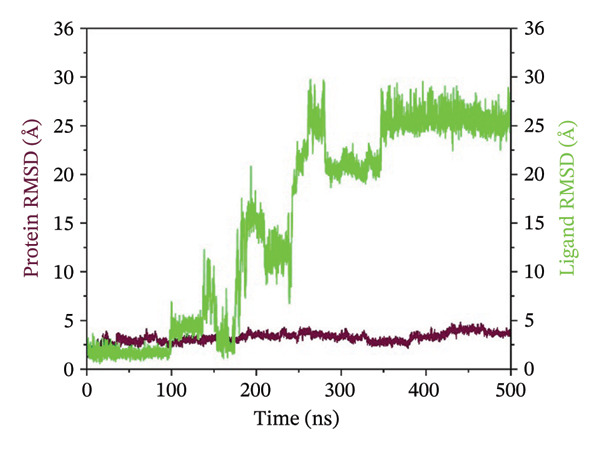
(d)

### 3.4. Root Mean Square Fluctuation (RMSF) Analysis of Protein

The RMSF plots reveal the flexibility of individual protein residues throughout the simulation. All complexes exhibited higher fluctuations at terminal regions, as expected. Key residues within the binding pocket showed minimal deviations, confirming a stable ligand interaction. Notably, the N protein when complexed with24292974 maintained lower RMSF values, whereas the protein in 24,330,502, 17,515,455 and Gamma‐Fagarine exhibited higher fluctuations at specific binding residues (Figure 3).

FIGURE 3Root mean square fluctuation (RMSF) of the protein residues. The plots of the RMSF of the HMPV nucleocapsid in complex with (a) 24,330,502, (b) 24,292,974, (c) 17,515,455, and (d) Gamma‐fagarine are shown. From the plots of the RMSFs of the different inhibitors with the HMPV nucleocapsid protein, the per‐residue fluctuations can be easily visualized. From the different plots obtained above, the inhibitor binding site has a low value of RMSF.

**Figure:**
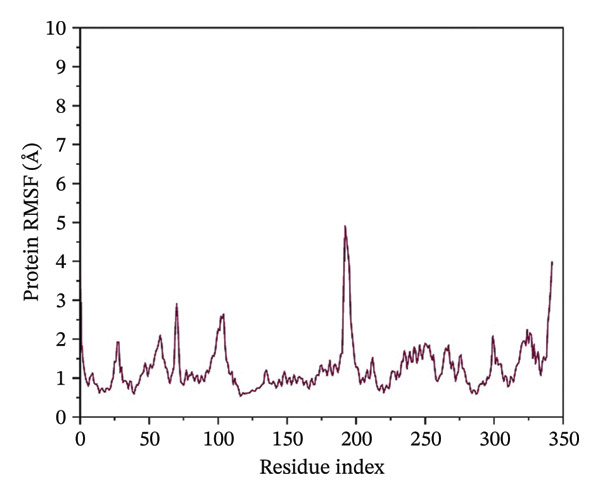
(a)

**Figure:**
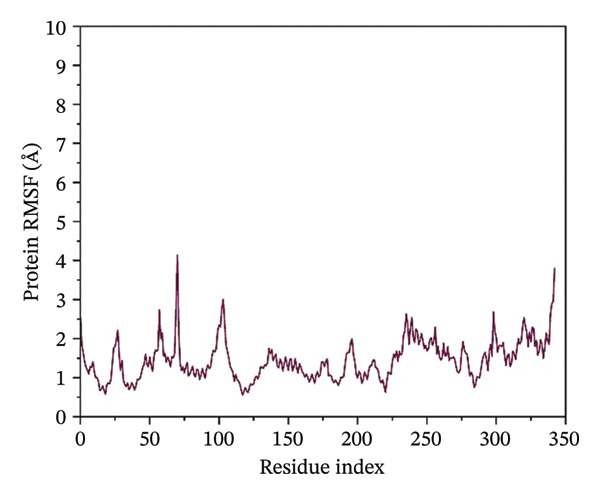
(b)

**Figure:**
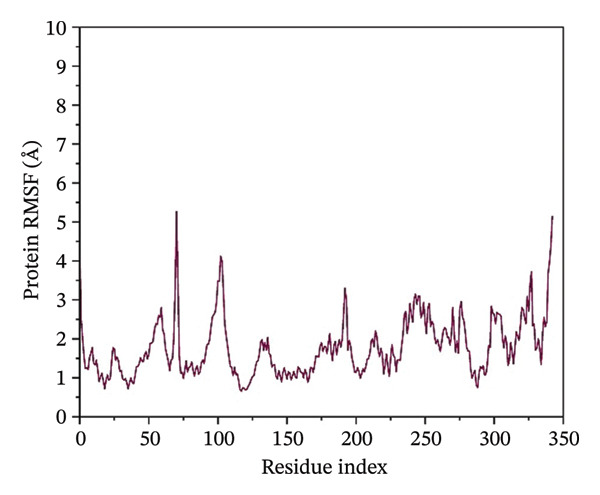
(c)

**Figure:**
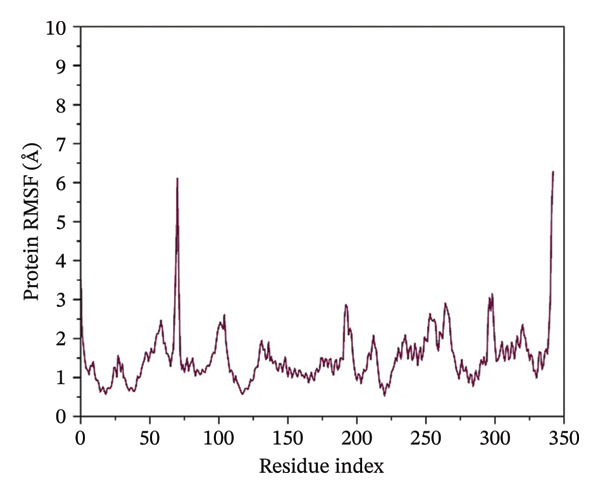
(d)

### 3.5. Root Mean Square Fluctuation (RMSF) Analysis of Ligand

Ligand flexibility was assessed using ligand RMSF plots. 24,330,502 exhibited the least ligand fluctuation, suggesting a strong and stable binding mode. 24,292,974 and 17,515,455 displayed moderate to high flexibility, especially in the case of 24,292,974, which indicates that the ligand might be able to take up various poses within the binding pocket. Such flexibility might call for a cautious approach while interpreting the results for binding stability. However, Gamma‐Fagarine complex displayed extreme fluctuations, with ligand RMSF values reaching 12–15 Å, reinforcing the observation of ligand instability from the RMSD analysis (Figure 4).

FIGURE 4Ligand RMSF analysis showing the internal flexibility of the compounds during simulation. Ligand fluctuation profiles for (a) 24,330,502, (b) 24,292,974, (c) 17,515,455, and (d) Gamma‐fagarine are presented. The minimal fluctuations of 24,330,502 confirm its strong and rigid binding, while the reference compound exhibits high instability, reinforcing its lower binding potential.

**Figure:**
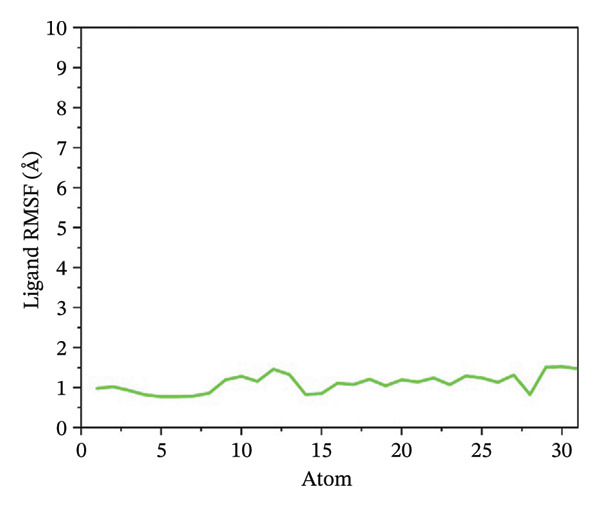
(a)

**Figure:**
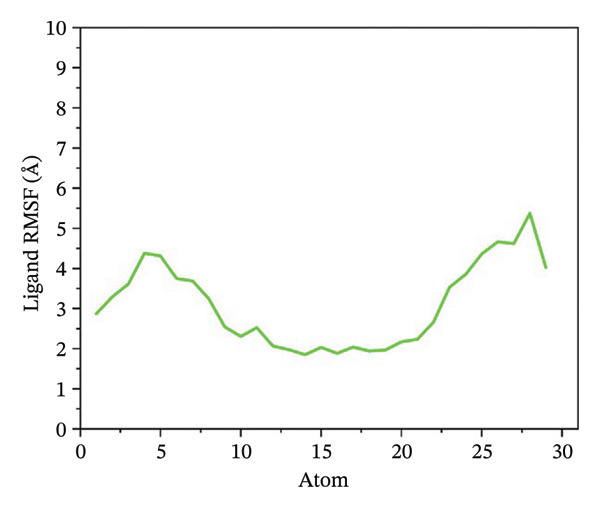
(b)

**Figure:**
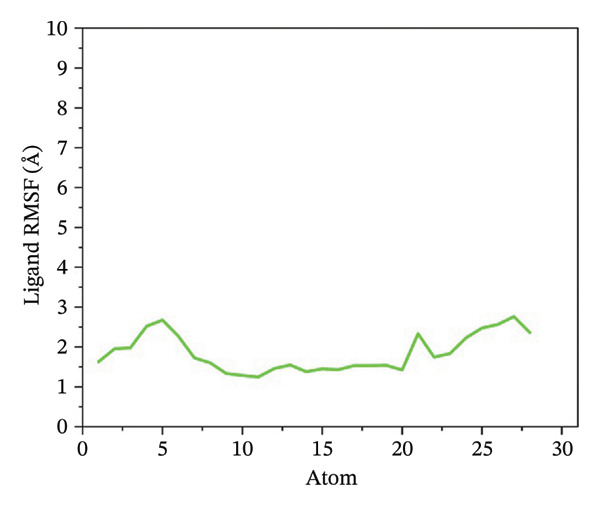
(c)

**Figure:**
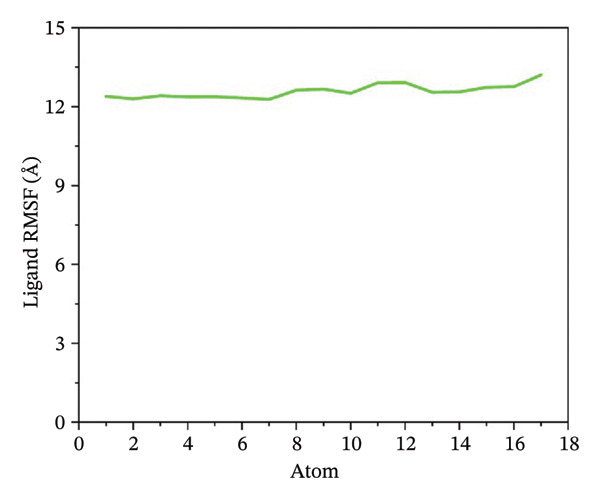
(d)

### 3.6. PCA

PCA was used to determine the conformational space of the protein–ligand complexes over the course of the 500 ns MD trajectory. The PCA plots for the complexes provide information on the structural dynamics and flexibility of the complexes. The plots also provide information on the significant changes in the conformations of the complexes over the course of the trajectory.

The PCA plots for the complex 24,330,502 have well‐defined clusters, indicating that the complex has relatively stable conformations over the course of the trajectory. The fact that the points on the PCA plots cluster in a single region of the conformational space indicates that the complex has few structural fluctuations. The interpretation of the PCA results for the complex 24,330,502 is supported by the eigenvalue plots. The eigenvalue plots for the complex 24,330,502 indicate that the first two principal components of the complex, namely PC1 and PC2, contribute 54.1% and 15.1% of the total variance, respectively. The structural homogeneity of the complex indicates that the ligand is tightly held and that the protein’s dynamics is not altered. The fact that the complex has few transitions to different states indicates that the complex has a strong binding affinity.

For complex 24,292,974, PCA plot observations indicate that there is significant clustering of points accompanied by some degree of dispersion of points. This implies moderate flexibility in protein conformation. There is also an observable change in distribution of points in PC1 and PC2, indicating some degree of conformational change in the protein structure during the simulation process.

The eigenvalue plot for complex 24,292,974 indicates that PC1 contributes 50.1% to the total variance of the protein structure, while PC2 contributes 20.9%, thus indicating a moderate degree of conformational flexibility as opposed to complex 24,330,502.

For complex 17,515,455, PCA plot observations indicate significant clustering of points accompanied by a moderate degree of separation between clusters of points. This implies that this complex may have undergone different conformational states that could be related to different ligand orientations or protein structure. This is also supported by PCA plot observations that indicate changes in conformation as shown by transitions in the plot.

The eigenvalue plot for complex 17,515,455 indicates that PC1 contributes 51.1% to the total variance of protein structure, while PC2 contributes 22.7%, thus indicating significant flexibility in protein structure as ligands assume different binding orientations.

For the Gamma‐Fagarine complex, PCA plot observations indicate significant flexibility in protein structure as shown by wide dispersion of points in the plot without significant clustering of points. This implies that this complex may have undergone significant changes in protein structure as shown by wide dispersion of points in PCA plot observations.

The eigenvalue plot for the Gamma‐Fagarine complex indicates that PC1 contributes 56.0% to the total variance of protein structure, while PC2 contributes 18.2%, thus indicating significant fluctuations in protein structure as ligands assume different binding orientations (Figure 5).

FIGURE 5Principal component analysis (PCA) plots illustrating conformational sampling of the protein–ligand complexes. PCA for (a) 24,330,502, (b) 24,292,974, (c) 17,515,455, and (d) Gamma‐fagarine highlight the structural dynamics across the first two principal components. The compact clustering for 24,330,502 and 24,292,974 indicates stable conformational states, while wider dispersion for the reference compound reflects extensive structural transitions and low binding retention.

**Figure:**
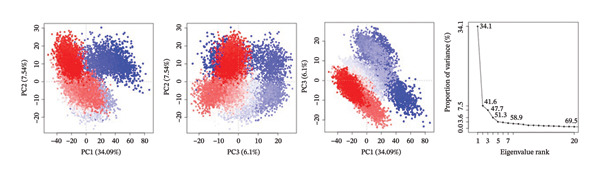
(a)

**Figure:**
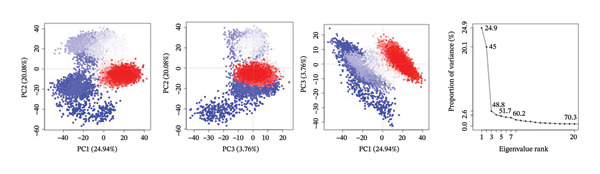
(b)

**Figure:**
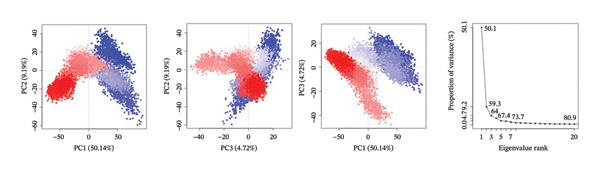
(c)

**Figure:**
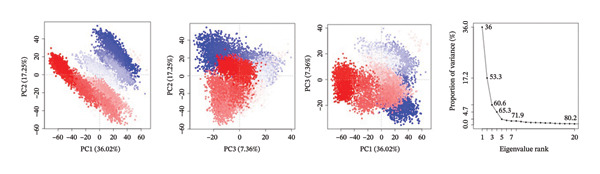
(d)

### 3.7. FEL Analysis

The analysis of the protein–ligand complexes’ stability and the determination of the energy minima have been performed by the application of the FEL analysis. FEL plots are graphical representations of the distribution of the Gibbs free energy of the system along the first two principal components of the simulation trajectory. The most stable state of the system corresponds to the low‐energy region of the plot, colored blue. The lower the energy minima, the higher the stability of system. A deep and broad energy minimum indicates a high level of stability and a good state of the protein‐ligand interaction.

For the protein–ligand complex 24,330,502, the FEL plot indicates a deep and well‐defined energy basin, which corresponds to a highly stable state of the system. The low free energy regions of the plot, colored blue, are concentrated, indicating that the system was in a state of stability with no significant changes in the protein‐ligand interaction. This high level of energy stability indicates the presence of a well‐defined protein–ligand interaction landscape, which corresponds to the presence of a high level of binding affinity and inhibitory efficacy. In contrast, the FEL plot of the Gamma Fagarine ligand indicates shallow and fragmented energy minima, corresponding to a highly unstable state of the system and a low level of protein‐ligand interaction.

In the case of 24,292,974, it can be seen that there are multiple energy minima, though with a slightly more spread‐out distribution pattern when compared to 24,330,502. This indicates that the system has accessed multiple conformations within a relatively stable regime. The presence of multiple shallow minima indicates that there is moderate flexibility in the system, enabling it to make small changes in its structure while remaining relatively stable.

As can be seen from the FEL for 17,515,455, there is a relatively dispersed energy distribution pattern, with a relatively high number of shallow minima when compared to complexes 24,330,502 and 24,292,974. This indicates that the system has accessed multiple conformations during the simulation. Though a relatively stable conformation for binding has been identified, a relatively high number of flexibility transitions indicate that there are possible changes in the ligand position.

As can be seen from the FEL for the Gamma‐Fagarine complex, it can be seen that there is a relatively fragmented and dispersed energy landscape, with a relatively high number of shallow minima. This indicates that there are relatively high levels of conformational changes, as well as relatively low levels of stability when compared to other complexes. The absence of a single relatively deep minimum indicates that there are relatively high levels of transitions in the structure of this ligand, indicating a relatively weak level of binding (Figure 6 and Figure S1).

FIGURE 62D free energy landscape (FEL) maps based on PC1 and PC2 projections. FEL plots of HMPV N protein bound to (a) 24,330,502, (b) 24,292,974, (c) 17,515,455, and (d) Gamma‐Fagarine show energy basins corresponding to stable conformations. The deeper and more localized basins for the selected inhibitors indicate energetically favorable and stable binding states, whereas the fragmented landscape for the reference reflects instability and frequent conformational shifts.

**Figure:**
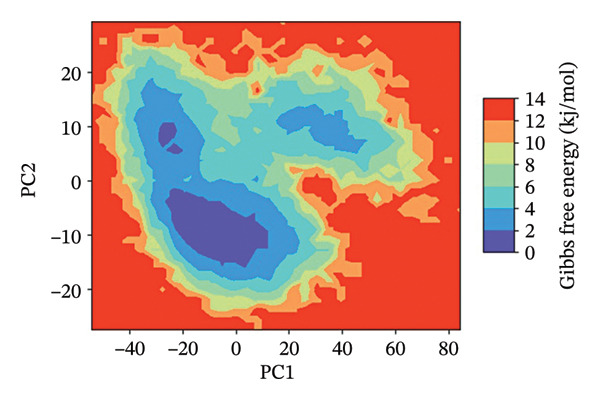
(a)

**Figure:**
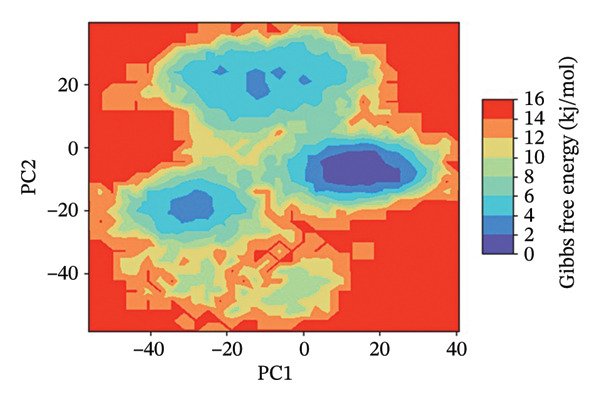
(b)

**Figure:**
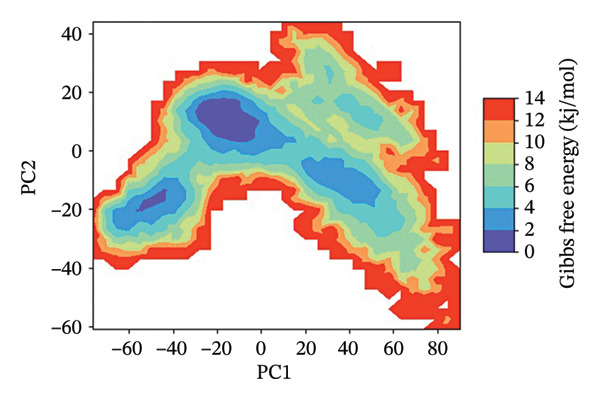
(c)

**Figure:**
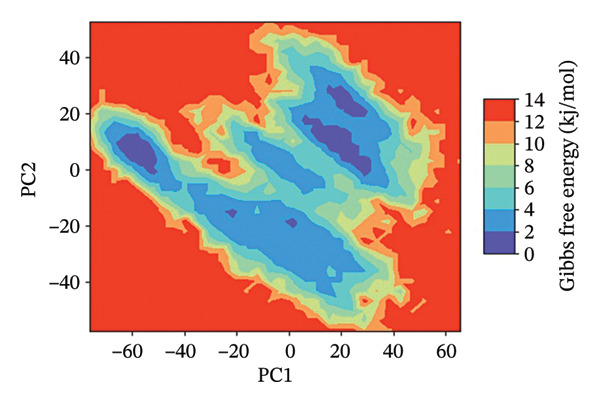
(d)

### 3.8. Structural Superimposition Analysis

**FIGURE 7:**
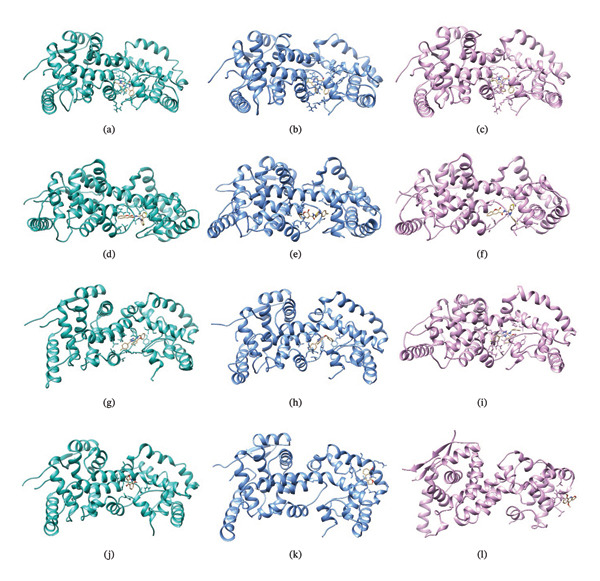
Top three low‐energy conformations extracted from the FEL for each ligand‐protein complex. Snapshots of (a–c) 24,330,502, (d–f) 24,292,974, (g–i) 17,515,455, and (j–l) Gamma‐fagarine display the diversity of stable binding poses observed during simulation. Consistent positioning within the binding pocket of selected inhibitors further confirms their strong, stable interactions with the HMPV nucleocapsid protein.

To investigate the stability of ligand–protein complexes in the simulation trajectory, three low‐energy conformations generated from each of the MD simulations (Figure 7) were superimposed on its initial docking poses. The RMSD values were estimated to calculate the extent of structural deviation that occurred between docked and simulated conformations (Figure 8). These results confirm that all of the complexes preserve their binding mode with very less structural deviation during simulation and, therefore, had stable interaction.

FIGURE 8Superimposition of initial docking poses and representative low‐energy structures extracted from MD simulations. Protein–ligand complexes for (a) 24,330,502, (b) 24,292,974, (c) 17,515,455, and (d) Gamma‐fagarine are superimposed to assess structural deviations.

**Figure:**
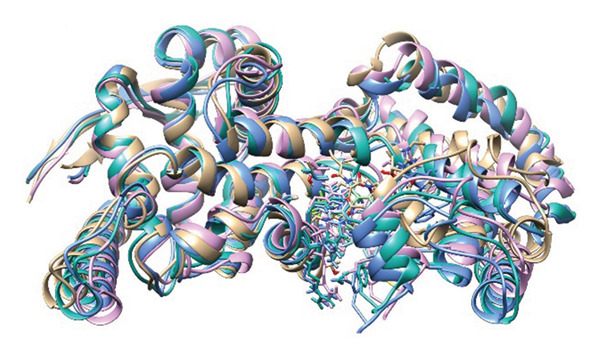
(a)

**Figure:**
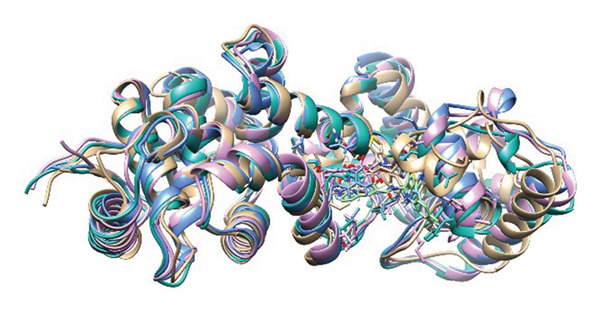
(b)

**Figure:**
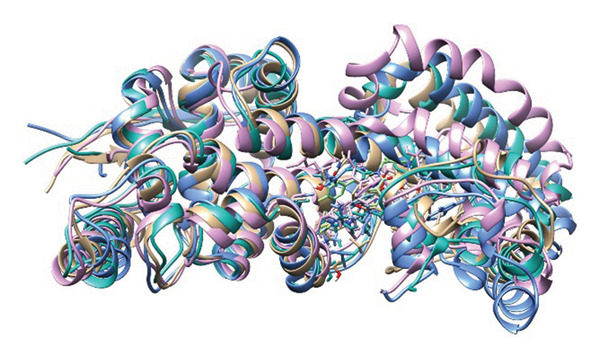
(c)

**Figure:**
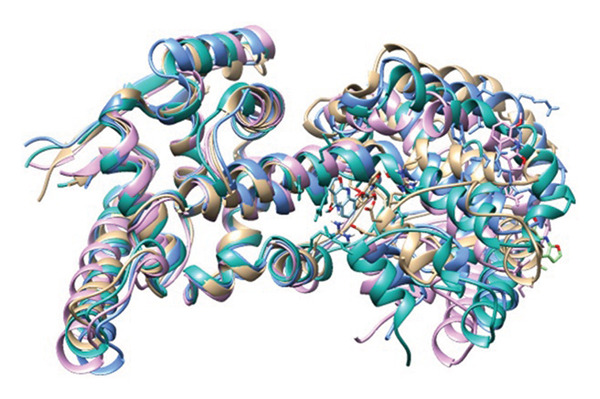
(d)

The RMSD of 1.928 Å obtained from 24,330,502 showed that it remained very stable. Due to the superimposition, one can see the low deviation of the docked structure with respect to the simulated one, indicating that the ligand kept a well‐preserved conformation during the binding process, which is supported by the low value of RMSD. This indicates that during the course of the simulation, the ligand remained tightly bound with the important interacting residues of the binding site, which enhances its potential to be a stable inhibitor.

An RMSD of 1.722 Å, 24,292,974 showed the least structural deviation, indicating that the ligand has undergone the least conformational drift from its initial docked pose. This indicates a highly stable interaction, in which the ligand remains largely bound within the protein’s active site. Superimposition supports that 24,292,974 maintained strong and consistent ligand–protein interactions, further validating its potential to be a reliable inhibitor.

The RMSD for 17,515,455 was 2.029 Å, reflecting a slightly larger deviation compared to the complexes 24,330,502 and 24,292,974. The superimposed structures reveal slight changes in the position of the ligands, suggesting that the ligand takes up different conformational states but stays in the binding pocket.

The Gamma‐Fagarine complex had an RMSD value of 1.855 Å, reflecting a moderate stability state. The ligand was found to be in the binding pocket, but the slight increase in the deviation compared to 24,292,974 suggests that the ligand was flexible and took up different conformations. The reference compound had a similar stability state but was not able to surpass the highest‐ranking inhibitors in binding strength.

### 3.9. Energetic Validation of Ligand Binding: MM/GBSA Analysis

The MM/GBSA binding free energy analysis provided insights into the thermodynamic stability of the chosen ligand–protein complexes. Among the ligands, the binding free energy of ligand 17,515,455 was the most favorable, at −74.57 ± 7.81 kcal/mol, indicating a stable binding mode with the HMPV nucleocapsid protein. The second‐best binding affinity was observed for ligand 24,330,502, with a binding free energy of −68.83 ± 4.47 kcal/mol. Ligand 24,292,974 showed a moderate binding profile, with a binding free energy of −45.75 ± 3.61 kcal/mol. In contrast, the reference compound Gamma‐Fagarine showed the lowest binding affinity (−24.89 ± 2.28 kcal/mol), thereby confirming the high binding affinities of the identified ligands.

The van der Waals interactions were significant in the binding energy of the ligands under study. Among the ligands, ligands 17,515,455 and 24,330,502 showed high van der Waals binding energy, at −54.52 and −53.73 kcal/mol, respectively. In addition, the ligands showed high lipophilic interactions, especially ligands 17,515,455 and 24,330,502, with ΔGBind Lipo values of −26.79 ± 1.56 and −21.01 ± 1.09 kcal/mol, respectively, thereby confirming the high hydrophobic complementarity of the ligands.

The electrostatic interactions showed a favorable binding profile for ligands 24,330,502 and 17,515,455, with ΔGBind Coulomb values of −5.57 ± 2.94 and −6.16 ± 2.70 kcal/mol, respectively. In contrast, ligand 24,292,974 showed a slightly unfavorable electrostatic binding profile, with a value of 3.21 ± 3.39 kcal/mol. In addition, the ligand strain and solvation penalties were found to be within the acceptable range, thereby confirming the high stability of the ligand‐protein complexes.

### 3.10. Swiss ADME Analysis

The ADME profiles of the selected compounds indicate favorable pharmacokinetic parameters, in addition to acceptable drug‐likeness. The compounds have shown excellent GI absorption and good results in drug‐likeness assessments, meeting the requirements of Lipinski, Veber, Ghose, and many others, in accordance with the established guidelines. Moreover, the compounds do not have PAINS or structural alerts, indicating a low likelihood of interfering with the assays. The compounds 24,330,502 and 24,292,974 have zero Lipinski violations, indicating they have the potential to be used in the development of lead‐like compounds.

Although the ADME profile of the selected compounds shows a good pharmacokinetic profile, some parameters deviate from the optimal ranges required for optimization (Table 2). For example, predictions of inhibition of CYP P450 enzymes, including CYP1A2, CYP2C19, and CYP3A4, indicate that further profiling would be required to determine the compound’s drug–drug interaction liabilities in preclinical development. Instead of being a limitation, this indicates the direction in which optimization is required. The blood–brain barrier (BBB) permeability of the compound, as indicated for BBB and for the compounds 24,330,502 and 17,515,455, would be a source of further optimization targets; however, this would require further investigation to determine the compound’s specificity for the brain. The moderate to low solubility of the compound 17,515,455 indicates that a strategy would be required to increase the aqueous solubility of the compound.

**TABLE 2:** The ADME analysis of the selected compounds.

Molecule	24,330,502	24,292,974	17,515,455	Reference
Canonical SMILES	O = C(c1cnn2c1nc(cc2C(F) (F)F)c1ccccc1)N1CCc2c(C1)cccc2	Fc1ccc(cc1)n1nc2c(c1NC(= O)c1cc3ccccc3oc1 = O)CSC2	Fc1ccc(cc1)c1nnc2n1c1ccccc1c(= O)n2Cc1ccccc1	COc1cccc2c1nc1occc1c2OC
Formula	C23H17F3N4O	C21H14FN3O3S	C22H15FN4O	C13H11NO3
MW	422.4	407.42	370.38	229.23
#Heavy atoms	31	29	28	17
#Aromatic heavy atoms	21	21	25	13
Fraction Csp3	0.17	0.1	0.05	0.15
#Rotatable bonds	4	4	3	2
#H‐bond acceptors	6	5	4	4
#H‐bond donors	0	1	0	0
MR	112.58	109.19	105.9	64.5
TPSA	50.5	102.43	52.19	44.49
iLOGP	3.28	3.16	3.19	2.52
XLOGP3	3.9	3.41	3.77	2.87
WLOGP	5.23	4.04	4.32	3
MLOGP	4.12	3.68	5.01	1.65
Silicos‐IT Log P	4.16	4.02	3.72	2.88
Consensus Log P	4.14	3.66	4	2.59
ESOL Log S	−5.15	−4.79	−4.97	−3.5
ESOL solubility (mg/mL)	2.97E − 03	6.67e − 03	3.93e − 03	7.20e − 02
ESOL solubility (mol/L)	7.03E − 06	1.64e − 05	1.06e − 05	3.14e − 04
ESOL class	Moderately soluble	Moderately soluble	Moderately soluble	Soluble
Ali Log S	−4.66	−5.24	−4.56	−3.46
Ali solubility (mg/mL)	9.27E − 03	2.34e − 03	1.02e − 02	7.88e − 02
Ali solubility (mol/L)	2.19E − 05	5.74e − 06	2.76e − 05	3.44e − 04
Ali class	Moderately soluble	Moderately soluble	Moderately soluble	Soluble
Silicos‐IT LogSw	−7.65	−7.51	−7.76	−4.85
Silicos‐IT solubility (mg/mL)	9.46E − 06	1.24e − 05	6.41e − 06	3.22e − 03
Silicos‐IT solubility (mol/L)	2.24E − 08	3.05e − 08	1.73e − 08	1.40e − 05
Silicos‐IT class	Poorly soluble	Poorly soluble	Poorly soluble	Moderately soluble
GI absorption	High	High	High	High
BBB permeant	Yes	No	Yes	Yes
Pgp substrate	Yes	No	No	No
CYP1A2 inhibitor	Yes	Yes	Yes	Yes
CYP2C19 inhibitor	Yes	Yes	Yes	Yes
CYP2C9 inhibitor	Yes	Yes	Yes	No
CYP2D6 inhibitor	No	No	No	Yes
CYP3A4 inhibitor	Yes	Yes	No	Yes
log Kp (cm/s)	−6.11	−6.36	−5.88	−5.66
Lipinski #violations	0	0	1	0
Ghose #violations	0	0	0	0
Veber #violations	0	0	0	0
Egan #violations	0	0	0	0
Muegge #violations	0	0	0	0
Bioavailability Score	0.55	0.55	0.55	0.55
PAINS #alerts	0	0	0	0
Brenk #alerts	0	1	0	0
Leadlikeness #violations	2	1	2	1
Synthetic accessibility	3.05	3.57	2.9	2.77

Overall, the ADME profile of the selected compound is favorable. The results would inform the future optimization of the lead compound based on the parameters identified.

## 4. Discussion

HMPV is a major respiratory pathogen with significant impact on infants, elderly, and immunocompromised patients. Despite its clinical significance, no antiviral drugs or vaccines are available; thus, there is a need to develop new therapeutic strategies against this infection. In this study, a computational drug discovery approach was employed to identify potential inhibitors targeting the HMPV nucleocapsid (N) protein, a crucial component of viral replication [51]. Identification of new inhibitors that specifically target the nucleocapsid protein of HMPV could provide therapeutic benefit. The nucleocapsid protein is a major structural component that encases viral RNA and has a crucial role in viral replication and protein synthesis through its interaction with phosphoproteins and RNA‐dependent RNA polymerase. Disrupting these proteins may inhibit ribonucleoprotein complex formation, thereby reducing viral replication. The nucleocapsid protein is highly stable and has a conserved sequence compared with the variable F and G glycoproteins. Therefore, there is significant potential in developing a broad‐spectrum antiviral. As there are currently no approved therapeutic agents for HMPV infection, recent small‐molecule inhibitors of nucleocapsid proteins provide a strong basis for developing therapeutic interventions to control viral load and disease severity. Using virtual screening, molecular docking, MD simulations, and binding free energy calculations [52], three promising compounds—24,330,502, 24,292,974, and 17,515,455—were identified. These compounds showed significant binding affinities ranging from −10.7 to −10.3 kcal/mol, compared with the reference compound Gamma‐Fagarine (−6.3 kcal/mol). The molecular interaction analysis revealed that these inhibitors formed significant hydrogen bonds, hydrophobic interactions, and *π*‐*π* stacking interactions with critical amino acids such as Leu167, Ile176, Val178, Ala254, Trp261, Ile334, and Met337, which play a significant role in nucleocapsid function. Previous studies conducted on nucleocapsid inhibitors targeting RSV and influenza had shown that these interactions play a significant role in their stability [53–55].

MD simulations were carried out to confirm the stability of the compounds under physiological conditions. The root mean square deviation (RMSD) analysis revealed that compounds 24,330,502 and 24,292,974 showed minimal fluctuations, around 2–4 Å, whereas compound 17,515,455 showed moderate deviations, around 4–6 Å, and the reference compound showed substantial instability, around 12–15 Å. The RMSF analysis also revealed that the binding site residues were stable in the presence of these compounds, which is consistent with earlier studies that showed that compounds having low RMSF values are likely to be potent inhibitors [18].

The PCA showed that compounds 24,330,502 and 24,292,974 clustered more tightly, suggesting they are more stable, whereas compound 17,515,455 and the reference compound clustered more loosely, indicating they are unstable.

The FEL analysis showed that compound 24,330,502 has deep energy basins, indicating stability, whereas the reference compound has a shallow energy distribution, indicating instability. Further superimposition analysis confirmed that 24,330,502 and 24,292,974 retained their docking conformations with very low RMSDs, approximately 1.7–1.9 Å, thus justifying high binding retention.

The MM/GBSA calculation revealed that compounds 17,515,455 (−74.57 kcal/mol), 24,330,502 (−68.83 kcal/mol), and 24,292,974 (−45.75 kcal/mol) have much stronger binding free energies than that of Gamma‐Fagarine (−24.89 kcal/mol). These findings indicate the stability of the interactions, which are mainly due to the van der Waals and lipophilic interactions, similar to the previous findings on viral nucleocapsid inhibitors [56].

Moreover, these compounds showed acceptable pharmacokinetic properties during the ADME analysis. Given their strong binding affinity, stability, and bioenergetic properties, these compounds will likely act as effective HMPV nucleocapsid inhibitors. These will need in vitro and in vivo studies to confirm their antiviral action and optimize their pharmacokinetic properties for possible clinical application. Although the pocket itself has a favorable structural and energetic profile for small‐molecule binding, it is also essential to consider that Nucleocapsid proteins tend to have large RNA‐binding interfaces, making them inherently difficult to target. Although the chosen pocket is expected to be easily accessible and manipulable, it may not fully capture the complexity of RNA encapsidation in a cell. Additional experiments will be required to determine whether a ligand binding to the pocket can modulate nucleocapsid–RNA interaction in a biological system.

## 5. Conclusion

This study used an integrative computational strategy to identify and characterize potential inhibitors of the HMPV nucleocapsid protein, which is crucial for viral replication and genomic encapsidation. Using computational techniques such as structure‐based virtual screening, molecular docking, and MD simulations, three compounds, namely, 24,330,502, 24,292,974, and 17,515,455, were identified as potential inhibitors of the HMPV nucleocapsid protein due to their strong binding affinities, structural stability, and consistency of interactions with the HMPV nucleocapsid protein in comparison to the reference compound Gamma‐Fagarine.

Although the study showed promising results for potential inhibitors of the HMPV nucleocapsid protein, it also had some limitations. First, the study considered only in silico simulation results, which, although effective, cannot fully represent real biological systems because factors such as compound solubility, permeability, stability, and interactions were not accounted for. Second, the role of the nucleocapsid protein in viral replication has not yet been validated in HMPV infection.

To further validate the study’s results, some experimental validation is recommended. Some methods, such as in vitro tests using surface plasmon resonance, microscale thermophoresis, or thermal shift assays, can be used to validate the study results and assess the antiviral potential of the identified compounds in HMPV‐infected cell cultures. Additionally, cytotoxicity and pharmacokinetic tests will be essential for validating the compounds’ potential as antiviral agents.

## Funding

This study was funded by the Northern Border University, through project number (NBU‐CRP‐2026‐2042).

## Conflicts of Interest

The authors declare no conflicts of interest.

## Supporting Information

Additional supporting information can be found online in the Supporting Information section.

## Supporting information


**Supporting Information** Supporting Table S1: Supporting Table S1 presents the virtual screening results of potential inhibitors against the HMPV nucleocapsid protein. Each entry includes the compound ID, docking energy, number of rotatable bonds (nRot), lead‐likeness status, number of hydrogen bond acceptors (HBA) and donors (HBD), LogP values, molecular weight (MW), and topological polar surface area (TPSA). Supporting Figure S1: Supporting Figure S1 shows the 3D free energy landscape (FEL) plots of the HMPV nucleocapsid protein complexed with four compounds: (a) 24,330,502, (b) 24,292,974, (c) 17,515,455, and (d) Gamma‐Fagarine, highlighting the conformational stability and dynamics of the protein–ligand complexes.

## Data Availability

The data that support the findings of this study are available in the supporting information of this article.
