# Supplementary material for: Human Metapneumovirus Nucleocapsid Inhibitors Discovery for Targeting Viral Replication and Genome Encapsidation: An In Silico Approach
Source: J Trop Med. 2026 May 20;2026:3511825. doi: 10.1155/jotm/3511825 (PMC13191828; doi:10.1155/jotm/3511825)
Supplement: Supplementary file 1 — Supporting Information Supporting Table S1: Supporting Table S1 presents the virtual screening results of potential inhibitors against the HMPV nucleocapsid protein. Each entry includes the compound ID, docking energy, number of rotatable bonds (nRot), lead‐likeness status, number of hydrogen bond acceptors (HBA) and donors (HBD), LogP values, molecular weight (MW), and topological polar surface area (TPSA). Supporting Figure S1: Supporting Figure S1 shows the 3D free energy landscape (FEL) plots of the HMPV nucleocapsid protein complexed with four compounds: (a) 24,330,502, (b) 24,292,974, (c) 17,515,455, and (d) Gamma‐Fagarine, highlighting the conformational stability and dynamics of the protein–ligand complexes. [file JOTM-2026-3511825-s001.docx]

**Table S1.** Virtual screening results of potential inhibitors against the HMPV nucleocapsid protein, including compound ID, docking energy, number of rotatable bonds (nRot), lead-likeness status, hydrogen bond acceptors (HBA), hydrogen bond donors (HBD), LogP, molecular weight (MW), and topological polar surface area (TPSA).

| **Compound_ID** | | **Energy** | **nRot** | **isLeadLike** | **HBA** | **HBD** | **LogP** | **MW** | **TPSA** |
| --- | --- | --- | --- | --- | --- | --- | --- | --- | --- |
| 24330502 | -10.7 | | 3 | Y | 5 | 0 | 3.9 | 422.4024896 | 50.5 |
| 24292974 | -10.3 | | 3 | Y | 6 | 1 | 3.41 | 407.4175632 | 102.43 |
| 17515455 | -10.3 | | 3 | Y | 5 | 0 | 3.77 | 370.3791032 | 52.19 |
| 124955471 | -10.3 | | 2 | Y | 6 | 2 | 2.39 | 369.41916 | 86.69 |
| 50123103 | -10.2 | | 1 | Y | 5 | 0 | 3.93 | 351.46854 | 84.73 |
| 17467814 | -10.2 | | 1 | Y | 6 | 2 | 2.16 | 357.3621 | 84.29 |
| 26615592 | -10.2 | | 4 | Y | 7 | 0 | 2.45 | 403.4769 | 75.36 |
| 24799563 | -10.1 | | 5 | Y | 6 | 1 | 3.94 | 432.55788 | 63.45 |
| 24303420 | -10 | | 4 | Y | 5 | 1 | 3.75 | 405.51262 | 64.79 |
| 846125 | -10 | | 6 | Y | 7 | 1 | 3.8 | 403.4769 | 73.85 |
| 7965742 | -10 | | 2 | Y | 6 | 0 | 2.91 | 373.40456 | 68.34 |
| 4256901 | -10 | | 4 | Y | 7 | 1 | 2.92 | 387.3153896 | 88.89 |
| 26649374 | -10 | | 1 | Y | 7 | 2 | 2.43 | 366.41368 | 87.32 |
| 26614353 | -10 | | 3 | Y | 6 | 0 | 3.64 | 377.4148032 | 63.91 |
| 24792652 | -9.9 | | 3 | Y | 6 | 1 | 2.32 | 353.3767 | 72.18 |
| 26539518 | -9.9 | | 3 | Y | 6 | 1 | 2.5 | 370.35756 | 79.62 |
| 7968389 | -9.9 | | 2 | Y | 5 | 0 | 3.49 | 345.3729432 | 46.32 |
| 7977853 | -9.9 | | 5 | Y | 7 | 0 | 3.84 | 414.41012 | 81.75 |
| 137276007 | -9.9 | | 3 | Y | 7 | 0 | 2.38 | 359.40772 | 99.09 |
| 26615264 | -9.9 | | 4 | Y | 7 | 1 | 2.81 | 428.4103696 | 79.18 |
| 49719963 | -9.9 | | 4 | Y | 7 | 0 | 3.22 | 400.4482 | 90.25 |
| 3713616 | -9.8 | | 4 | Y | 7 | 0 | 2.82 | 397.4012 | 111.92 |
| 49822060 | -9.8 | | 4 | Y | 6 | 2 | 3.35 | 412.52188 | 85.15 |
| 81066370 | -9.8 | | 2 | Y | 4 | 0 | 3.54 | 343.37528 | 48.42 |
| 865942 | -9.8 | | 1 | Y | 7 | 2 | 3.07 | 375.42374 | 92.15 |
| 26641090 | -9.8 | | 5 | Y | 7 | 3 | 3.81 | 429.89666 | 88.69 |
| 49820828 | -9.8 | | 5 | Y | 7 | 1 | 3.57 | 449.49568 | 99.88 |
| 17437878 | -9.8 | | 0 | Y | 7 | 3 | 2.35 | 436.2151 | 118.35 |
| 26616001 | -9.8 | | 4 | Y | 7 | 0 | 2.1 | 383.48726 | 75.36 |
| 57267624 | -9.8 | | 3 | Y | 6 | 0 | 3.33 | 409.50132 | 78.96 |
| 51087141 | -9.7 | | 4 | Y | 7 | 0 | 1.99 | 368.456 | 101.58 |
| 29216802 | -9.7 | | 1 | Y | 4 | 0 | 3.66 | 331.3430632 | 39.42 |
| 49824952 | -9.7 | | 5 | Y | 7 | 1 | 2.91 | 407.41594 | 99.88 |
| 24782861 | -9.7 | | 4 | Y | 5 | 1 | 3.74 | 373.3797432 | 63.99 |
| 26651313 | -9.7 | | 3 | Y | 6 | 0 | 3.28 | 407.459 | 65.07 |
| 24364317 | -9.7 | | 4 | Y | 6 | 2 | 3.57 | 391.44298 | 100.3 |
| 134958875 | -9.7 | | 3 | Y | 7 | 1 | 2.98 | 425.50072 | 98.93 |
| 87339068 | -9.7 | | 4 | Y | 7 | 0 | 3.34 | 383.40268 | 76.53 |
| 49718577 | -9.7 | | 3 | Y | 6 | 1 | 2.76 | 376.45156 | 66.71 |
| 26647296 | -9.7 | | 4 | Y | 5 | 2 | 2.81 | 406.5173 | 59.06 |
| 121285583 | -9.7 | | 5 | Y | 6 | 0 | 3.74 | 424.49436 | 60.25 |
| 24371081 | -9.7 | | 1 | Y | 5 | 0 | 3.37 | 389.83434 | 54.68 |
| 124949859 | -9.7 | | 4 | Y | 5 | 2 | 2.9 | 385.45832 | 62.58 |
| 26729838 | -9.7 | | 3 | Y | 6 | 0 | 2.4 | 359.33162 | 78.51 |
| 24819698 | -9.7 | | 7 | Y | 6 | 1 | 3.37 | 416.4955 | 93.22 |
| 17409822 | -9.7 | | 4 | Y | 5 | 0 | 3.35 | 396.50256 | 67.76 |
| 26651069 | -9.7 | | 5 | Y | 7 | 0 | 2.41 | 378.42438 | 81.35 |
| 24414966 | -9.6 | | 3 | Y | 5 | 1 | 2.92 | 395.4035632 | 96.72 |
| 24372207 | -9.6 | | 4 | Y | 6 | 1 | 3.48 | 387.51572 | 61.88 |
| 24337068 | -9.6 | | 3 | Y | 7 | 0 | 1.8 | 403.4952 | 92.1 |
| 17458391 | -9.6 | | 3 | Y | 6 | 0 | 2.74 | 384.4305 | 59.73 |
| 24408901 | -9.6 | | 5 | Y | 6 | 1 | 3.2 | 397.4691032 | 90.42 |
| 24831296 | -9.6 | | 2 | Y | 5 | 0 | 1.95 | 319.35718 | 55.2 |
| 26613648 | -9.6 | | 4 | Y | 5 | 1 | 3.09 | 396.4546032 | 58.64 |
| 4250141 | -9.6 | | 6 | Y | 7 | 1 | 3.62 | 401.50738 | 62.79 |
| 24308358 | -9.6 | | 4 | Y | 7 | 0 | 2.38 | 371.39198 | 85.61 |
| 26648583 | -9.6 | | 3 | Y | 5 | 0 | 3.37 | 391.4380832 | 55.2 |
| 24828508 | -9.6 | | 2 | Y | 6 | 1 | 2.38 | 389.3743296 | 81.96 |
| 99356498 | -9.6 | | 2 | Y | 4 | 0 | 3.91 | 371.4748 | 47.34 |
| 17476177 | -9.5 | | 3 | Y | 7 | 1 | 3.09 | 347.32422 | 97.04 |
| 26648699 | -9.5 | | 4 | Y | 7 | 1 | 2.07 | 395.3837232 | 92.36 |
| 7971098 | -9.5 | | 5 | Y | 7 | 0 | 3.37 | 430.52538 | 91.93 |
| 17442950 | -9.5 | | 1 | Y | 7 | 1 | 2.8 | 332.74172 | 96.5 |
| 26615198 | -9.5 | | 5 | Y | 6 | 0 | 3.75 | 397.49062 | 96.58 |
| 49828307 | -9.5 | | 4 | Y | 7 | 2 | 1.47 | 408.49342 | 83.44 |
| 124948833 | -9.5 | | 3 | Y | 6 | 2 | 4 | 409.52124 | 78.45 |
| 49723687 | -9.5 | | 4 | Y | 7 | 1 | 3.48 | 438.95308 | 75.94 |
| 3716046 | -9.5 | | 2 | Y | 4 | 0 | 3.23 | 352.46992 | 40.62 |
| 24403263 | -9.5 | | 5 | Y | 6 | 0 | 3.87 | 398.4538632 | 87.77 |
| 26614671 | -9.5 | | 3 | Y | 6 | 2 | 3.75 | 403.4736 | 84.08 |
| 49673431 | -9.5 | | 5 | Y | 6 | 2 | 3.51 | 401.45772 | 82.08 |
| 24374311 | -9.5 | | 5 | Y | 6 | 0 | 2.42 | 431.5235432 | 69.31 |
| 26651038 | -9.5 | | 2 | Y | 4 | 1 | 3.64 | 371.85728 | 49.77 |
| 49823190 | -9.5 | | 4 | Y | 6 | 0 | 3.66 | 406.47424 | 62.99 |
| 847986 | -9.5 | | 1 | Y | 5 | 0 | 2.93 | 298.2982 | 60.15 |
| 24370709 | -9.5 | | 4 | Y | 6 | 1 | 2.46 | 411.5172 | 90.79 |
| 3716957 | -9.5 | | 3 | Y | 5 | 0 | 3.35 | 349.37986 | 59.75 |
| 26616690 | -9.5 | | 3 | Y | 6 | 1 | 1.46 | 361.44022 | 65.12 |
| 26755511 | -9.5 | | 3 | Y | 6 | 1 | 2.59 | 400.3539096 | 76.18 |
| 57259006 | -9.5 | | 5 | Y | 7 | 1 | 3.25 | 401.41466 | 96.27 |
| 103158923 | -9.5 | | 4 | Y | 6 | 0 | 3.86 | 395.83892 | 74.92 |
| 85239749 | -9.5 | | 4 | Y | 7 | 1 | 2.92 | 359.38128 | 74.09 |
| 104233102 | -9.5 | | 1 | Y | 4 | 0 | 3.14 | 338.37706 | 76.91 |
| 24813706 | -9.5 | | 4 | Y | 7 | 1 | 3 | 416.44918 | 71.75 |
| 24297017 | -9.5 | | 5 | Y | 5 | 2 | 3.34 | 345.39446 | 75.04 |
| 26648717 | -9.5 | | 3 | Y | 6 | 1 | 3.35 | 403.4736 | 73.22 |
| 124896714 | -9.4 | | 4 | Y | 6 | 1 | 3.7 | 349.38316 | 65.5 |
| 17504626 | -9.4 | | 6 | Y | 7 | 2 | 3.57 | 441.47852 | 95.58 |
| 24401642 | -9.4 | | 5 | Y | 6 | 1 | 3.87 | 350.3464032 | 73.07 |
| 24798694 | -9.4 | | 4 | Y | 6 | 1 | 3.25 | 426.5269432 | 66.74 |
| 24304623 | -9.4 | | 6 | Y | 6 | 1 | 3.51 | 413.46842 | 73.1 |
| 26535038 | -9.4 | | 2 | Y | 4 | 0 | 3.02 | 315.38678 | 54.99 |
| 26535572 | -9.4 | | 2 | Y | 6 | 1 | 2.27 | 364.43924 | 112.33 |
| 49823247 | -9.4 | | 4 | Y | 6 | 0 | 3.73 | 392.44766 | 62.99 |
| 14743867 | -9.4 | | 4 | Y | 7 | 1 | 1.52 | 375.42374 | 71.76 |
| 124949191 | -9.4 | | 5 | Y | 7 | 0 | 3.59 | 448.55728 | 60.51 |
| 4251799 | -9.4 | | 5 | Y | 6 | 1 | 2.37 | 389.44702 | 79.37 |
| 89855298 | -9.4 | | 6 | Y | 5 | 0 | 3.06 | 389.44372 | 63.68 |
| 24327285 | -9.4 | | 2 | Y | 5 | 2 | 2.76 | 373.85654 | 100.15 |
| 24798666 | -9.4 | | 5 | Y | 6 | 0 | 3.74 | 403.4736 | 57.79 |
| 24317930 | -9.4 | | 3 | Y | 6 | 0 | 3.44 | 355.41088 | 108.95 |
| 26724544 | -9.4 | | 6 | Y | 7 | 1 | 2.45 | 448.5077032 | 93.32 |
| 26615228 | -9.4 | | 5 | Y | 7 | 1 | 2.49 | 411.5205 | 112.12 |
| 47194007 | -9.4 | | 2 | Y | 6 | 1 | 1.09 | 353.2958696 | 73.1 |
| 26539601 | -9.4 | | 2 | Y | 6 | 1 | 2.47 | 290.2762 | 84.67 |
| 24832596 | -9.4 | | 3 | Y | 7 | 0 | 2.03 | 361.3508 | 83.19 |
| 99361075 | -9.4 | | 1 | Y | 7 | 1 | 1.52 | 312.30332 | 113.86 |
| 866113 | -9.4 | | 3 | Y | 6 | 2 | 3.03 | 359.42434 | 71.31 |
| 26614060 | -9.4 | | 3 | Y | 7 | 0 | -0.13 | 369.3928032 | 71.33 |
| 26532431 | -9.4 | | 4 | Y | 6 | 0 | 2.78 | 361.39386 | 66.65 |
| 26615604 | -9.4 | | 4 | Y | 7 | 2 | 1.46 | 362.42828 | 86.8 |
| 24836875 | -9.3 | | 4 | Y | 6 | 1 | 3.88 | 413.53308 | 78.1 |
| 7967938 | -9.3 | | 4 | Y | 6 | 1 | 2.5 | 400.49126 | 84.09 |
| 26659235 | -9.3 | | 4 | Y | 5 | 0 | 3.97 | 329.352 | 57.13 |
| 26732296 | -9.3 | | 6 | Y | 5 | 0 | 3.88 | 420.4760032 | 45.92 |
| 14729669 | -9.3 | | 6 | Y | 6 | 0 | 3.67 | 434.5274 | 52.93 |
| 49647816 | -9.3 | | 4 | Y | 4 | 1 | 3.98 | 312.36784 | 50.7 |
| 49674091 | -9.3 | | 3 | Y | 4 | 2 | 3.84 | 334.41158 | 50.86 |
| 24400906 | -9.3 | | 3 | Y | 7 | 1 | 2.75 | 383.42098 | 102.58 |
| 14722456 | -9.3 | | 5 | Y | 7 | 2 | 1.56 | 373.42616 | 111.74 |
| 26651394 | -9.3 | | 5 | Y | 4 | 1 | 3.79 | 416.4361296 | 45.47 |
| 24405036 | -9.3 | | 3 | Y | 7 | 0 | 2.37 | 363.36668 | 77.69 |
| 56320127 | -9.3 | | 5 | Y | 7 | 2 | 1.51 | 368.3616832 | 95.16 |
| 26659026 | -9.3 | | 0 | Y | 5 | 0 | 2.99 | 306.36172 | 52.19 |
| 144098297 | -9.3 | | 4 | Y | 6 | 0 | 2.94 | 414.51784 | 75.3 |
| 7997204 | -9.3 | | 4 | Y | 7 | 2 | 3.31 | 435.51546 | 105.65 |
| 3711669 | -9.3 | | 3 | Y | 5 | 1 | 2.7 | 365.44876 | 87.07 |
| 49649103 | -9.3 | | 5 | Y | 5 | 0 | 3.95 | 410.4100896 | 78.78 |
| 26671289 | -9.3 | | 0 | Y | 4 | 2 | 3.82 | 355.18544 | 66.08 |
| 24781665 | -9.3 | | 4 | Y | 6 | 1 | 3.78 | 411.4013664 | 75.02 |
| 24370847 | -9.3 | | 6 | Y | 7 | 1 | 3.74 | 437.51142 | 107.75 |
| 14726080 | -9.3 | | 6 | Y | 6 | 1 | 4 | 345.39776 | 64.34 |
| 49820660 | -9.3 | | 3 | Y | 7 | 2 | 1.54 | 384.42566 | 102.84 |
| 24815980 | -9.3 | | 4 | Y | 5 | 0 | 2.77 | 337.3740832 | 81.27 |
| 103163258 | -9.3 | | 6 | Y | 7 | 1 | 2.8 | 432.51482 | 88.33 |
| 24323142 | -9.3 | | 4 | Y | 7 | 2 | 2.91 | 427.5166 | 118.61 |
| 26650965 | -9.3 | | 5 | Y | 7 | 0 | 3.04 | 394.3989632 | 80.82 |
| 4252352 | -9.3 | | 2 | Y | 7 | 1 | 1.68 | 363.36668 | 82.33 |
| 85269551 | -9.3 | | 3 | Y | 6 | 0 | 1.85 | 390.4285632 | 75.3 |
| 26613772 | -9.3 | | 3 | Y | 7 | 2 | 1.41 | 352.3655832 | 86.8 |
| 124949961 | -9.3 | | 5 | Y | 6 | 1 | 3.74 | 402.87132 | 60.03 |
| 26641954 | -9.3 | | 4 | Y | 5 | 1 | 3.96 | 361.4154032 | 55.11 |
| 56316296 | -9.3 | | 7 | Y | 6 | 0 | 3.42 | 385.41526 | 74.08 |
| 124949875 | -9.3 | | 5 | Y | 7 | 1 | 2.4 | 436.52666 | 116.57 |
| 134465100 | -9.3 | | 6 | Y | 7 | 1 | 2.61 | 421.48888 | 74.87 |
| 26724519 | -9.3 | | 5 | Y | 7 | 1 | 2.42 | 436.50022 | 84.94 |
| 24792453 | -9.3 | | 3 | Y | 7 | 0 | 1.93 | 430.90786 | 91.85 |
| 24816325 | -9.3 | | 5 | Y | 6 | 0 | 2.59 | 347.39372 | 98.34 |
| 56315995 | -9.3 | | 3 | Y | 6 | 1 | 2.27 | 354.3318032 | 79.28 |
| 49720215 | -9.3 | | 3 | Y | 6 | 0 | 3.61 | 384.4488 | 81.02 |
| 49715100 | -9.3 | | 5 | Y | 6 | 2 | 2.38 | 435.94752 | 121.31 |
| 99361023 | -9.3 | | 3 | Y | 6 | 4 | 2.52 | 368.3336496 | 121.07 |
| 14745330 | -9.2 | | 5 | Y | 6 | 2 | 3.43 | 399.4882 | 75.6 |
| 81080219 | -9.2 | | 5 | Y | 7 | 1 | 1.62 | 407.48874 | 104.28 |
| 24798599 | -9.2 | | 5 | Y | 7 | 0 | 2.94 | 417.45712 | 73.66 |
| 865038 | -9.2 | | 3 | Y | 6 | 3 | 3.97 | 317.3446 | 89.86 |
| 3716809 | -9.2 | | 4 | Y | 7 | 2 | 2.55 | 345.3547 | 90.98 |
| 14746053 | -9.2 | | 0 | Y | 6 | 3 | 3.37 | 413.46842 | 99.58 |
| 26650872 | -9.2 | | 5 | Y | 4 | 1 | 3.81 | 388.54838 | 46.83 |
| 24820099 | -9.2 | | 2 | Y | 5 | 0 | 3.56 | 331.32152 | 68.87 |
| 57257021 | -9.2 | | 3 | Y | 5 | 0 | 2.59 | 335.39964 | 49.58 |
| 26617008 | -9.2 | | 4 | Y | 7 | 0 | 2.63 | 393.48208 | 62.97 |
| 49821019 | -9.2 | | 3 | Y | 5 | 1 | 2.87 | 333.38376 | 58.22 |
| 121283359 | -9.2 | | 4 | Y | 5 | 0 | 2.32 | 386.48944 | 43.73 |
| 24781276 | -9.2 | | 7 | Y | 6 | 1 | 3.93 | 420.50082 | 71.78 |
| 26647141 | -9.2 | | 5 | Y | 5 | 1 | 3.08 | 410.4596664 | 51.36 |
| 49728809 | -9.2 | | 6 | Y | 7 | 1 | 2.47 | 442.50964 | 83.77 |
| 3712274 | -9.2 | | 3 | Y | 6 | 0 | 3.67 | 442.28588 | 84.68 |
| 124948481 | -9.2 | | 5 | Y | 7 | 1 | 2.36 | 417.50348 | 80.12 |
| 26642263 | -9.2 | | 4 | Y | 6 | 0 | 3.04 | 393.4556432 | 112.92 |
| 57268692 | -9.2 | | 6 | Y | 6 | 1 | 2.54 | 380.43696 | 67.87 |
| 26614438 | -9.2 | | 3 | Y | 7 | 2 | 0.84 | 377.43962 | 85.09 |
| 857966 | -9.2 | | 1 | Y | 6 | 1 | 1.96 | 324.33064 | 79.61 |
| 26637496 | -9.2 | | 6 | Y | 7 | 1 | 1.91 | 438.4945832 | 83.77 |
| 24366537 | -9.2 | | 2 | Y | 7 | 2 | 3.34 | 364.3978 | 100.5 |
| 24841355 | -9.2 | | 2 | Y | 6 | 0 | 3.39 | 350.75854 | 80.45 |
| 24787544 | -9.2 | | 1 | Y | 4 | 0 | 3.13 | 368.4461 | 76.66 |
| 14728557 | -9.2 | | 3 | Y | 4 | 0 | 3.88 | 318.32276 | 52.58 |
| 14722701 | -9.2 | | 9 | Y | 6 | 1 | 3.71 | 437.50812 | 88.36 |
| 26535162 | -9.2 | | 3 | Y | 7 | 0 | 3.83 | 392.45748 | 144.02 |
| 24346926 | -9.2 | | 4 | Y | 7 | 1 | 3.57 | 400.83882 | 121.27 |
| 7965488 | -9.2 | | 2 | Y | 4 | 1 | 2.43 | 296.3636 | 45.48 |
| 99303153 | -9.2 | | 6 | Y | 7 | 0 | 3.6 | 407.4623 | 77.57 |
| 24302409 | -9.2 | | 1 | Y | 6 | 0 | 2.09 | 359.37798 | 63.91 |
| 4263584 | -9.2 | | 6 | Y | 7 | 3 | 2.31 | 379.40914 | 92.45 |
| 103060609 | -9.2 | | 6 | Y | 6 | 1 | 4 | 422.5167 | 67.87 |
| 7973436 | -9.2 | | 3 | Y | 5 | 0 | 3.53 | 385.45832 | 45.67 |
| 26729757 | -9.2 | | 4 | Y | 6 | 0 | 3.75 | 368.3153496 | 69.38 |
| 26650220 | -9.2 | | 5 | Y | 6 | 2 | 3.21 | 408.51656 | 109.46 |
| 85268729 | -9.2 | | 5 | Y | 6 | 1 | 2.4 | 393.45886 | 87.75 |
| 49717583 | -9.2 | | 4 | Y | 7 | 0 | 2.19 | 417.4539032 | 96.03 |
| 24329802 | -9.2 | | 5 | Y | 7 | 0 | 2.79 | 378.42438 | 63.86 |
| 103050604 | -9.2 | | 3 | Y | 4 | 1 | 3.62 | 325.3633 | 54.88 |
| 24789719 | -9.2 | | 2 | Y | 7 | 1 | 3.55 | 348.30898 | 105.12 |
| 24839324 | -9.2 | | 5 | Y | 7 | 1 | 2.56 | 444.31386 | 83.36 |
| 17458027 | -9.2 | | 4 | Y | 6 | 0 | 2.86 | 394.46354 | 60.36 |
| 26614556 | -9.2 | | 2 | Y | 7 | 0 | 1.96 | 334.32876 | 70.65 |
| 85268996 | -9.2 | | 7 | Y | 7 | 2 | 3.13 | 429.46782 | 91.31 |
| 144095829 | -9.2 | | 1 | Y | 5 | 0 | 2.05 | 299.32932 | 48.53 |
| 85199142 | -9.2 | | 4 | Y | 6 | 2 | 2.66 | 343.4201 | 82.08 |
| 24799140 | -9.2 | | 5 | Y | 5 | 0 | 3.27 | 387.3766664 | 63.68 |
| 24826645 | -9.2 | | 5 | Y | 7 | 1 | 3.33 | 373.3351696 | 84.98 |
| 862844 | -9.2 | | 3 | Y | 5 | 1 | 3.88 | 357.45152 | 49.25 |
| 104223069 | -9.1 | | 3 | Y | 5 | 3 | 2.29 | 317.3413 | 77.12 |
| 14739966 | -9.1 | | 0 | Y | 6 | 2 | 2.9 | 429.51088 | 96.42 |
| 3716351 | -9.1 | | 5 | Y | 7 | 0 | 2.63 | 348.30898 | 95.43 |
| 24320355 | -9.1 | | 3 | Y | 6 | 1 | 2.91 | 364.3945 | 73.99 |
| 24799561 | -9.1 | | 6 | Y | 6 | 1 | 3.62 | 416.8300232 | 73.59 |
| 14741401 | -9.1 | | 4 | Y | 6 | 1 | 3.4 | 343.38188 | 72.18 |
| 7966851 | -9.1 | | 4 | Y | 6 | 1 | 3.46 | 415.54896 | 87.75 |
| 24823819 | -9.1 | | 6 | Y | 7 | 1 | 3.16 | 362.35874 | 99.36 |
| 49665171 | -9.1 | | 6 | Y | 6 | 2 | 3.02 | 429.5076632 | 90.65 |
| 859636 | -9.1 | | 4 | Y | 7 | 2 | 2.3 | 351.35598 | 95.58 |
| 24791669 | -9.1 | | 2 | Y | 4 | 1 | 3.56 | 314.33736 | 59.06 |
| 24385470 | -9.1 | | 4 | Y | 6 | 0 | 3.48 | 377.43632 | 68.46 |
| 49819821 | -9.1 | | 7 | Y | 7 | 2 | 3.21 | 407.41594 | 108.67 |
| 26543076 | -9.1 | | 5 | Y | 7 | 1 | 3.03 | 360.36604 | 94.05 |
| 24332821 | -9.1 | | 4 | Y | 5 | 2 | 3.04 | 337.36916 | 82.53 |
| 24797974 | -9.1 | | 4 | Y | 7 | 1 | 2.07 | 360.3612264 | 74.08 |
| 17474959 | -9.1 | | 4 | Y | 4 | 3 | 3.19 | 369.4557032 | 92.78 |
| 24826178 | -9.1 | | 4 | Y | 5 | 0 | 3.69 | 348.3951 | 55.57 |
| 49672457 | -9.1 | | 4 | Y | 5 | 1 | 2.65 | 398.3993896 | 74.86 |
| 24345972 | -9.1 | | 3 | Y | 6 | 0 | 1.78 | 412.50196 | 84.83 |
| 89854998 | -9.1 | | 2 | Y | 5 | 0 | 3.09 | 328.36724 | 44.87 |
| 57261250 | -9.1 | | 5 | Y | 6 | 1 | 3.63 | 438.7843696 | 79.62 |
| 24367925 | -9.1 | | 4 | Y | 6 | 3 | 3.06 | 340.41946 | 86.88 |
| 26646194 | -9.1 | | 2 | Y | 6 | 0 | 2.77 | 375.4668 | 53.74 |
| 7966095 | -9.1 | | 4 | Y | 6 | 2 | 2.63 | 352.43016 | 83.98 |
| 24782867 | -9.1 | | 3 | Y | 7 | 2 | 3.06 | 431.4837 | 89.55 |
| 26614559 | -9.1 | | 4 | Y | 6 | 1 | 3.14 | 404.45836 | 69.56 |
| 57261201 | -9.1 | | 5 | Y | 6 | 1 | 2.72 | 411.5172 | 81.86 |
| 4258277 | -9.1 | | 5 | Y | 5 | 0 | 3.66 | 375.4635 | 49.58 |
| 26648623 | -9.1 | | 3 | Y | 6 | 1 | 1.35 | 380.4634 | 107.19 |
| 49672465 | -9.1 | | 6 | Y | 6 | 2 | 2.87 | 411.5172 | 90.65 |
| 26669376 | -9.1 | | 3 | Y | 6 | 0 | 2.98 | 378.46744 | 51.39 |
| 17460783 | -9.1 | | 3 | Y | 6 | 0 | 3.43 | 363.38982 | 101.79 |
| 57266109 | -9.1 | | 7 | Y | 7 | 2 | 2.63 | 449.95096 | 103.96 |
| 17480568 | -9.1 | | 3 | Y | 5 | 1 | 3.08 | 359.3714632 | 84.76 |
| 49677576 | -9.1 | | 4 | Y | 7 | 2 | 0.94 | 366.41368 | 88.64 |
| 3711493 | -9.1 | | 3 | Y | 5 | 1 | 3.45 | 329.352 | 68.02 |
| 26670238 | -9.1 | | 4 | Y | 6 | 1 | 2.45 | 344.2691064 | 92.09 |
| 24429208 | -9.1 | | 1 | Y | 5 | 1 | 2.99 | 290.31926 | 62.86 |
| 17506049 | -9.1 | | 4 | Y | 5 | 0 | 3.06 | 372.4165 | 59.38 |
| 24411705 | -9.1 | | 6 | Y | 7 | 1 | 2.93 | 421.4474432 | 114.55 |
| 17432513 | -9.1 | | 6 | Y | 7 | 0 | 2.95 | 438.56084 | 100.75 |
| 24355125 | -9.1 | | 2 | Y | 5 | 1 | 3.05 | 350.3431032 | 62.55 |
| 26649867 | -9.1 | | 4 | Y | 5 | 1 | 3.76 | 350.41098 | 64.24 |
| 49643148 | -9.1 | | 3 | Y | 5 | 1 | 3.57 | 327.4207 | 63.99 |
| 14731146 | -9.1 | | 3 | Y | 6 | 1 | 2.33 | 393.43242 | 85.94 |
| 99356049 | -9.1 | | 2 | Y | 5 | 1 | 3.32 | 289.28814 | 71.59 |
| 14727391 | -9.1 | | 1 | Y | 4 | 0 | 3.46 | 329.1834 | 43.07 |
| 124947980 | -9.1 | | 5 | Y | 5 | 2 | 3.92 | 383.52702 | 61.38 |
| 14746016 | -9.1 | | 3 | Y | 6 | 0 | 2.5 | 362.42498 | 58.44 |
| 26756439 | -9.1 | | 2 | Y | 7 | 1 | 1.18 | 354.3351032 | 99.94 |
| 14744508 | -9.1 | | 5 | Y | 7 | 1 | 3.41 | 415.46768 | 114.08 |
| 4244923 | -9.1 | | 4 | Y | 7 | 2 | 2.27 | 369.3464432 | 85.35 |
| 57257019 | -9.1 | | 5 | Y | 7 | 1 | 2.67 | 416.49066 | 93.32 |
| 24799867 | -9.1 | | 5 | Y | 6 | 1 | 2.9 | 386.4630832 | 61.46 |
| 49719520 | -9.1 | | 4 | Y | 7 | 0 | 1.56 | 412.4573032 | 69.36 |
| 4244418 | -9.1 | | 4 | Y | 5 | 0 | 3.26 | 410.4811832 | 49.85 |
| 99358243 | -9.1 | | 1 | Y | 5 | 0 | 2.1 | 351.39574 | 65.37 |
| 17512238 | -9.1 | | 8 | Y | 7 | 0 | 3.74 | 445.46392 | 93.89 |
| 850637 | -9.1 | | 4 | Y | 7 | 0 | 3.36 | 425.54708 | 97.8 |
| 22413290 | -9.1 | | 3 | Y | 6 | 0 | 2.6 | 365.37926 | 68.98 |
| 17402568 | -9.1 | | 4 | Y | 6 | 1 | 1.69 | 375.4668 | 54.6 |
| 49674814 | -9.1 | | 5 | Y | 7 | 0 | 2.93 | 325.27234 | 102.33 |
| 24307983 | -9.1 | | 6 | Y | 6 | 2 | 3.32 | 401.45772 | 87.05 |
| 26648830 | -9 | | 5 | Y | 7 | 1 | 2.75 | 419.473 | 82.45 |
| 24332919 | -9 | | 6 | Y | 6 | 1 | 3.3 | 367.42158 | 112.79 |
| 56319845 | -9 | | 6 | Y | 4 | 1 | 3.62 | 368.49246 | 74.71 |
| 14731765 | -9 | | 5 | Y | 7 | 0 | 3.34 | 440.54012 | 66.63 |
| 50102600 | -9 | | 4 | Y | 6 | 0 | 3.23 | 365.4057 | 101.6 |
| 56463773 | -9 | | 5 | Y | 4 | 0 | 3.86 | 394.4817832 | 40.62 |
| 17460213 | -9 | | 2 | Y | 4 | 1 | 3.69 | 369.25838 | 45.63 |
| 26658121 | -9 | | 2 | Y | 5 | 2 | 3.74 | 353.75588 | 86.39 |
| 17446154 | -9 | | 5 | Y | 6 | 1 | 3.53 | 385.39208 | 83.75 |
| 4251394 | -9 | | 7 | Y | 7 | 0 | 1.59 | 383.4409 | 81.5 |
| 26533963 | -9 | | 3 | Y | 7 | 1 | 3.47 | 388.20754 | 85.57 |
| 104223076 | -9 | | 8 | Y | 6 | 1 | 2.89 | 401.45772 | 81.06 |
| 24361855 | -9 | | 4 | Y | 6 | 0 | 3.66 | 412.4373 | 70.08 |
| 47198102 | -9 | | 3 | Y | 4 | 0 | 3.91 | 447.36208 | 68.28 |
| 49731663 | -9 | | 5 | Y | 5 | 1 | 2.76 | 384.85604 | 58.64 |
| 24799765 | -9 | | 5 | Y | 5 | 0 | 3.24 | 352.4053432 | 55.05 |
| 17507113 | -9 | | 4 | Y | 6 | 0 | 3.91 | 380.39874 | 73.93 |
| 124948823 | -9 | | 4 | Y | 5 | 2 | 2.94 | 386.3919064 | 67.43 |
| 26648557 | -9 | | 3 | Y | 6 | 0 | 1.58 | 348.3984 | 68.09 |
| 85267933 | -9 | | 1 | Y | 4 | 0 | 2.64 | 274.2735 | 50.27 |
| 26635752 | -9 | | 4 | Y | 6 | 1 | 3.94 | 430.60666 | 97.54 |
| 4240945 | -9 | | 2 | Y | 6 | 3 | 2.83 | 351.42548 | 114.4 |
| 3711386 | -9 | | 1 | Y | 4 | 0 | 3.16 | 305.3273 | 50.52 |
| 57257148 | -9 | | 4 | Y | 6 | 0 | 2.92 | 378.42108 | 62.99 |
| 51090542 | -9 | | 7 | Y | 6 | 0 | 2.88 | 407.48544 | 99.38 |
| 22413404 | -9 | | 4 | Y | 6 | 3 | 2.22 | 357.44668 | 87.3 |
| 49823806 | -9 | | 5 | Y | 6 | 1 | 2.34 | 335.35658 | 81.18 |
| 3714443 | -9 | | 4 | Y | 6 | 0 | 2.86 | 381.44816 | 80.65 |
| 862807 | -9 | | 4 | Y | 6 | 0 | 2.87 | 391.41654 | 68.98 |
| 26618425 | -9 | | 5 | Y | 6 | 1 | 3.7 | 391.4629 | 77.25 |
| 24351727 | -9 | | 1 | Y | 4 | 0 | 3.86 | 305.37366 | 47.78 |
| 24794431 | -9 | | 5 | Y | 7 | 1 | 1.97 | 378.42438 | 76.46 |
| 121285760 | -9 | | 5 | Y | 6 | 2 | 3.56 | 389.3279696 | 76.05 |
| 26648432 | -9 | | 4 | Y | 6 | 0 | 2.57 | 376.45156 | 60.25 |
| 7967802 | -9 | | 5 | Y | 7 | 0 | 3.62 | 428.4367 | 86.72 |
| 26696844 | -9 | | 4 | Y | 7 | 0 | 3.47 | 373.4343 | 118.47 |
| 24827864 | -9 | | 4 | Y | 5 | 1 | 3.69 | 340.80678 | 59.81 |
| 4255939 | -9 | | 4 | Y | 6 | 1 | 2.45 | 387.4958 | 78.1 |
| 26724238 | -9 | | 5 | Y | 7 | 3 | 2.44 | 360.3628232 | 115.9 |
| 26616899 | -9 | | 4 | Y | 7 | 0 | 2.3 | 381.42502 | 73.66 |
| 24361358 | -9 | | 4 | Y | 6 | 0 | 1.75 | 365.42562 | 62.62 |
| 17480798 | -9 | | 6 | Y | 7 | 1 | 2.81 | 402.39942 | 94.47 |
| 49718414 | -9 | | 3 | Y | 6 | 1 | 3.08 | 349.42952 | 62.53 |
| 17433165 | -9 | | 4 | Y | 6 | 0 | 3.47 | 381.3786864 | 65.08 |
| 4251079 | -9 | | 5 | Y | 7 | 1 | 1.18 | 380.45856 | 106.2 |
| 26643029 | -9 | | 7 | Y | 6 | 2 | 3.65 | 446.58446 | 69.64 |
| 26617143 | -9 | | 7 | Y | 7 | 1 | 3.47 | 413.53638 | 110.05 |
| 24322397 | -9 | | 2 | Y | 5 | 3 | 3.04 | 342.43204 | 82 |
| 24308701 | -9 | | 1 | Y | 5 | 0 | 3.27 | 355.45394 | 82.92 |
| 22409257 | -9 | | 5 | Y | 7 | 1 | 3.37 | 449.52212 | 109 |
| 26642425 | -9 | | 3 | Y | 5 | 2 | 3.52 | 391.50596 | 61.44 |
| 7970408 | -9 | | 5 | Y | 7 | 0 | 2.23 | 369.44076 | 97.92 |
| 50110359 | -9 | | 7 | Y | 7 | 1 | 3.78 | 409.43182 | 79.16 |
| 24780261 | -9 | | 7 | Y | 6 | 2 | 3.21 | 375.4668 | 72.34 |
| 17508602 | -9 | | 6 | Y | 7 | 1 | 3 | 424.83378 | 86.63 |
| 4257606 | -9 | | 5 | Y | 6 | 1 | 2.34 | 379.40584 | 83.91 |
| 26647196 | -9 | | 7 | Y | 7 | 1 | 1.19 | 398.4307232 | 72.64 |
| 26616657 | -9 | | 2 | Y | 6 | 0 | 2.85 | 365.45206 | 93.32 |
| 24406217 | -9 | | 4 | Y | 6 | 0 | 2.98 | 385.47992 | 75.03 |
| 17461528 | -9 | | 3 | Y | 7 | 1 | 2.7 | 361.37724 | 117.14 |
| 24816637 | -9 | | 3 | Y | 5 | 0 | 2.9 | 384.49186 | 66.07 |
| 22402839 | -9 | | 8 | Y | 7 | 1 | 3.25 | 435.4691 | 86.99 |
| 850035 | -9 | | 3 | Y | 7 | 2 | 2.24 | 408.49342 | 73.91 |
| 17482982 | -9 | | 7 | Y | 6 | 3 | 3.76 | 434.50748 | 106.87 |
| 24785340 | -9 | | 5 | Y | 6 | 0 | 3.16 | 389.44702 | 64.43 |
| 24298834 | -9 | | 2 | Y | 5 | 1 | 3.32 | 346.37922 | 62.55 |
| 26756438 | -9 | | 4 | Y | 6 | 1 | 2.49 | 371.38868 | 82.53 |
| 24324180 | -9 | | 4 | Y | 6 | 1 | 1.78 | 341.3843 | 98.11 |
| 17472505 | -9 | | 5 | Y | 5 | 0 | 2.61 | 330.38312 | 52.71 |
| 4262627 | -9 | | 3 | Y | 6 | 0 | 2.53 | 353.36856 | 68.98 |
| 49723513 | -9 | | 6 | Y | 7 | 1 | 2.99 | 401.49924 | 81.41 |
| 24815625 | -9 | | 5 | Y | 7 | 1 | 2.54 | 373.40786 | 81.41 |
| 124949835 | -9 | | 6 | Y | 6 | 1 | 3.45 | 422.4951832 | 59.39 |
| 24404863 | -9 | | 5 | Y | 6 | 1 | 2.46 | 412.7934496 | 68.92 |
| 49820496 | -9 | | 3 | Y | 6 | 1 | 3.06 | 381.4002032 | 57.8 |
| 56432199 | -9 | | 3 | Y | 7 | 2 | 1.72 | 327.3130632 | 92.67 |
| 49731692 | -9 | | 4 | Y | 6 | 0 | 3.11 | 443.4878832 | 93.04 |
| 49737376 | -9 | | 5 | Y | 6 | 1 | 3.21 | 428.52612 | 67.23 |
| 17437008 | -9 | | 3 | Y | 6 | 0 | 2.53 | 309.3193 | 78.49 |
| 14737318 | -9 | | 6 | Y | 7 | 1 | 3.34 | 441.49682 | 103.22 |
| 57266738 | -9 | | 2 | Y | 7 | 0 | 0.89 | 334.39342 | 83.89 |
| 26533752 | -9 | | 4 | Y | 7 | 2 | 2.35 | 341.3876 | 121.18 |
| 26625211 | -9 | | 5 | Y | 6 | 1 | 3.56 | 424.49274 | 117.23 |
| 26731264 | -9 | | 5 | Y | 7 | 1 | 1.99 | 428.4286696 | 90.99 |
| 26529251 | -9 | | 3 | Y | 7 | 1 | 0.95 | 326.34982 | 78.03 |
| 144095566 | -9 | | 4 | Y | 4 | 1 | 3.3 | 358.3818064 | 49.41 |
| 22404906 | -9 | | 6 | Y | 6 | 0 | 2.4 | 407.4639232 | 90.38 |
| 24783671 | -8.9 | | 5 | Y | 5 | 1 | 3.98 | 442.5278832 | 55.38 |
| 99454979 | -8.9 | | 4 | Y | 5 | 3 | 3.55 | 367.85526 | 108.19 |
| 3713489 | -8.9 | | 6 | Y | 7 | 0 | 3.67 | 431.52844 | 126.08 |
| 24343563 | -8.9 | | 2 | Y | 6 | 1 | 1.81 | 330.35836 | 91.76 |
| 24392640 | -8.9 | | 7 | Y | 7 | 1 | 3.86 | 419.473 | 90.29 |
| 14747128 | -8.9 | | 3 | Y | 6 | 0 | 3.14 | 352.41024 | 98.59 |
| 857605 | -8.9 | | 3 | Y | 6 | 2 | 2.49 | 317.3446 | 83.56 |
| 26528531 | -8.9 | | 5 | Y | 6 | 1 | 3.95 | 385.7762232 | 88.15 |
| 17485615 | -8.9 | | 4 | Y | 6 | 0 | 4 | 346.79144 | 87.36 |
| 26645406 | -8.9 | | 1 | Y | 5 | 3 | 3.62 | 352.42686 | 70.59 |
| 24827010 | -8.9 | | 4 | Y | 5 | 1 | 3.99 | 367.46464 | 92.78 |
| 49667825 | -8.9 | | 7 | Y | 7 | 2 | 2.59 | 395.40524 | 108.67 |
| 26539445 | -8.9 | | 5 | Y | 6 | 1 | 2.12 | 366.2913296 | 79.62 |
| 24411866 | -8.9 | | 4 | Y | 5 | 1 | 3.25 | 348.42154 | 88.35 |
| 103074695 | -8.9 | | 6 | Y | 7 | 0 | 3.52 | 407.4623 | 75.88 |
| 26541744 | -8.9 | | 3 | Y | 4 | 2 | 3.8 | 291.34708 | 54.02 |
| 24836159 | -8.9 | | 3 | Y | 6 | 1 | 3.08 | 338.40358 | 72.68 |
| 17515660 | -8.9 | | 5 | Y | 7 | 0 | 3.79 | 415.39488 | 91.67 |
| 24837004 | -8.9 | | 7 | Y | 7 | 2 | 1.59 | 405.49116 | 121.46 |
| 85272461 | -8.9 | | 7 | Y | 7 | 1 | 2.79 | 373.40786 | 81.93 |
| 85148402 | -8.9 | | 2 | Y | 2 | 1 | 3.69 | 305.3455432 | 32.86 |
| 4242996 | -8.9 | | 1 | Y | 6 | 0 | 2.74 | 348.3984 | 61.3 |
| 26647174 | -8.9 | | 5 | Y | 7 | 1 | 2.95 | 412.52518 | 70.95 |
| 3713162 | -8.9 | | 6 | Y | 7 | 0 | 2.87 | 392.46926 | 98.09 |
| 49732704 | -8.9 | | 4 | Y | 7 | 0 | 2.95 | 420.93296 | 59.31 |
| 144095453 | -8.9 | | 2 | Y | 5 | 3 | 3.13 | 288.30338 | 85.39 |
| 47196891 | -8.9 | | 4 | Y | 6 | 0 | 3.35 | 396.4115432 | 62.99 |
| 850419 | -8.9 | | 3 | Y | 6 | 1 | 2.14 | 379.4026232 | 84.45 |
| 24314569 | -8.9 | | 4 | Y | 7 | 0 | 3.06 | 441.4058296 | 66.88 |
| 24783609 | -8.9 | | 7 | Y | 7 | 1 | 3.51 | 398.3628464 | 81.66 |
| 85240364 | -8.9 | | 3 | Y | 7 | 0 | 3.64 | 419.4514832 | 66.88 |
| 124948206 | -8.9 | | 4 | Y | 5 | 1 | 3.42 | 443.9215632 | 90.54 |
| 858860 | -8.9 | | 1 | Y | 6 | 0 | 1.8 | 337.35254 | 101.68 |
| 124949211 | -8.9 | | 6 | Y | 7 | 1 | 1.73 | 383.4193832 | 77.39 |
| 26613658 | -8.9 | | 4 | Y | 6 | 1 | 1.78 | 374.45398 | 88.11 |
| 24800267 | -8.9 | | 6 | Y | 5 | 0 | 3.48 | 389.4685632 | 38.88 |
| 17447165 | -8.9 | | 5 | Y | 5 | 1 | 3.91 | 380.48002 | 59.59 |
| 24818559 | -8.9 | | 5 | Y | 5 | 0 | 3.74 | 420.4544864 | 42.66 |
| 26533870 | -8.9 | | 3 | Y | 4 | 1 | 3.79 | 329.3487 | 63.33 |
| 26614770 | -8.9 | | 3 | Y | 7 | 1 | 2.16 | 382.45614 | 76.46 |
| 17474468 | -8.9 | | 3 | Y | 5 | 2 | 3.48 | 348.80402 | 90.98 |
| 850616 | -8.9 | | 3 | Y | 5 | 1 | 3.63 | 287.31862 | 55.11 |
| 50085483 | -8.9 | | 6 | Y | 6 | 1 | 3.2 | 357.3621 | 84.94 |
| 24346215 | -8.9 | | 6 | Y | 7 | 2 | 2.87 | 431.89262 | 120.53 |
| 24322149 | -8.9 | | 3 | Y | 5 | 0 | 3.72 | 362.46804 | 50.5 |
| 49736537 | -8.9 | | 5 | Y | 5 | 1 | 3.15 | 342.3641632 | 58.64 |
| 22416599 | -8.9 | | 7 | Y | 6 | 1 | 3.18 | 386.44638 | 69.04 |
| 56432202 | -8.9 | | 5 | Y | 7 | 2 | 1.85 | 337.37576 | 92.67 |
| 26532594 | -8.9 | | 2 | Y | 5 | 2 | 2.7 | 288.30338 | 74.43 |
| 24343866 | -8.9 | | 7 | Y | 7 | 1 | 2.74 | 445.9192 | 111.04 |
| 17458828 | -8.9 | | 2 | Y | 4 | 0 | 3.28 | 291.34708 | 38.25 |
| 26632223 | -8.9 | | 3 | Y | 6 | 3 | 3.13 | 317.38282 | 94.82 |
| 49721261 | -8.9 | | 3 | Y | 7 | 1 | 2.45 | 389.51498 | 107.84 |
| 24279787 | -8.9 | | 3 | Y | 7 | 0 | 2.66 | 380.37398 | 133.14 |
| 125306931 | -8.9 | | 4 | Y | 6 | 2 | 2.84 | 369.3383296 | 78.51 |
| 51089749 | -8.9 | | 5 | Y | 6 | 3 | 1.32 | 400.49126 | 115.22 |
| 24409324 | -8.9 | | 3 | Y | 5 | 2 | 3.72 | 391.25906 | 59.59 |
| 14745619 | -8.9 | | 4 | Y | 7 | 1 | 1.54 | 337.3294 | 82.45 |
| 137275924 | -8.9 | | 4 | Y | 7 | 3 | 2.69 | 386.44968 | 98.72 |
| 24269353 | -8.9 | | 6 | Y | 7 | 1 | 3.96 | 449.52212 | 110.57 |
| 24836894 | -8.9 | | 5 | Y | 7 | 2 | 1.82 | 374.41092 | 115.15 |
| 26649507 | -8.9 | | 4 | Y | 7 | 0 | 2.99 | 380.40204 | 78.09 |
| 17509384 | -8.9 | | 6 | Y | 7 | 2 | 3.12 | 437.51142 | 128.37 |
| 3715732 | -8.9 | | 3 | Y | 5 | 1 | 2.67 | 335.2805896 | 67.75 |
| 56373850 | -8.9 | | 7 | Y | 6 | 0 | 3.93 | 418.4170632 | 72.64 |
| 24826757 | -8.9 | | 3 | Y | 7 | 3 | 2.38 | 358.39652 | 98.72 |
| 49825067 | -8.9 | | 5 | Y | 6 | 1 | 2.95 | 378.42108 | 81.43 |
| 49723997 | -8.9 | | 4 | Y | 4 | 0 | 3.76 | 371.4931 | 62.83 |
| 92763756 | -8.9 | | 1 | Y | 4 | 1 | 3.87 | 360.48708 | 47.92 |
| 124948428 | -8.9 | | 6 | Y | 5 | 2 | 3.48 | 384.85604 | 63.35 |
| 24799273 | -8.9 | | 3 | Y | 5 | 0 | 3.11 | 347.41034 | 55.2 |
| 24300412 | -8.9 | | 5 | Y | 6 | 3 | 2.7 | 365.42562 | 82.95 |
| 49821781 | -8.9 | | 3 | Y | 7 | 0 | 2.1 | 382.40978 | 68.31 |
| 121285821 | -8.9 | | 4 | Y | 7 | 1 | 3.01 | 426.3944896 | 88.72 |
| 49730811 | -8.9 | | 2 | Y | 6 | 0 | 1.89 | 352.44846 | 75.3 |
| 24794757 | -8.9 | | 4 | Y | 6 | 1 | 3.39 | 399.4882 | 72.7 |
| 24384347 | -8.9 | | 4 | Y | 4 | 1 | 3.65 | 360.3809832 | 49.41 |
| 92764346 | -8.9 | | 3 | Y | 5 | 0 | 3.55 | 334.36852 | 68.09 |
| 24327306 | -8.9 | | 7 | Y | 7 | 1 | 2.85 | 449.54204 | 80.76 |
| 22404882 | -8.9 | | 4 | Y | 6 | 0 | 3.6 | 364.42094 | 96.89 |
| 85267932 | -8.9 | | 1 | Y | 4 | 0 | 2.64 | 274.2735 | 50.27 |
| 24399504 | -8.9 | | 4 | Y | 6 | 1 | 2.63 | 391.4629 | 69.72 |
| 3711682 | -8.9 | | 6 | Y | 7 | 1 | 2.94 | 424.51596 | 100.64 |
| 17410693 | -8.9 | | 4 | Y | 4 | 0 | 3.68 | 352.3821232 | 78.51 |
| 56318698 | -8.9 | | 6 | Y | 6 | 2 | 2.84 | 446.31764 | 96.54 |
| 4256263 | -8.9 | | 7 | Y | 7 | 0 | 2.22 | 370.42552 | 104.43 |
| 24794805 | -8.9 | | 1 | Y | 3 | 0 | 3.81 | 291.2759032 | 39.07 |
| 85146758 | -8.9 | | 5 | Y | 6 | 0 | 3.54 | 338.31086 | 74.97 |
| 24800115 | -8.9 | | 6 | Y | 5 | 0 | 3.64 | 407.4805432 | 48.56 |
| 26647205 | -8.9 | | 8 | Y | 7 | 1 | 2.51 | 416.47236 | 78.27 |
| 24355144 | -8.9 | | 5 | Y | 7 | 1 | 1.77 | 397.44756 | 101.66 |
| 125258641 | -8.9 | | 4 | Y | 4 | 0 | 3.95 | 365.42716 | 58.68 |
| 17438727 | -8.9 | | 3 | Y | 4 | 1 | 3.09 | 317.338 | 57.61 |
| 124948047 | -8.9 | | 6 | Y | 7 | 2 | 2.37 | 432.94692 | 87.97 |
| 3713282 | -8.9 | | 2 | Y | 6 | 1 | 2.5 | 354.39968 | 83.19 |
| 49732264 | -8.9 | | 6 | Y | 5 | 1 | 3.63 | 380.48002 | 58.64 |
| 57268955 | -8.9 | | 7 | Y | 7 | 1 | 2.63 | 396.4628 | 111.41 |
| 14726647 | -8.9 | | 5 | Y | 6 | 2 | 3.34 | 377.43632 | 74.58 |
| 124949758 | -8.9 | | 6 | Y | 7 | 1 | 3.17 | 416.4508432 | 85.59 |
| 26646842 | -8.9 | | 5 | Y | 6 | 1 | 3.64 | 390.4102632 | 72.95 |
| 26669796 | -8.8 | | 5 | Y | 6 | 0 | 2.73 | 358.34686 | 76.57 |
| 57266734 | -8.8 | | 7 | Y | 7 | 1 | 3.79 | 400.81238 | 90.66 |
| 14727043 | -8.8 | | 2 | Y | 5 | 1 | 3.67 | 337.41552 | 63.99 |
| 26649856 | -8.8 | | 4 | Y | 6 | 1 | 3.94 | 380.43696 | 67.87 |
| 26647207 | -8.8 | | 4 | Y | 7 | 1 | 1.99 | 380.44026 | 84.39 |
| 14747072 | -8.8 | | 6 | Y | 5 | 1 | 3.75 | 392.3981096 | 85.11 |
| 859064 | -8.8 | | 4 | Y | 5 | 0 | 3.78 | 319.38362 | 81.79 |
| 56315809 | -8.8 | | 6 | Y | 7 | 0 | 3.01 | 436.89084 | 79.54 |
| 24799110 | -8.8 | | 7 | Y | 7 | 1 | 3.08 | 402.49214 | 72.04 |
| 49827226 | -8.8 | | 5 | Y | 6 | 1 | 2.09 | 371.38868 | 79.37 |
| 47203291 | -8.8 | | 3 | Y | 5 | 1 | 2.53 | 308.33124 | 66.48 |
| 26652781 | -8.8 | | 4 | Y | 6 | 1 | 0.98 | 339.36842 | 103 |
| 24387519 | -8.8 | | 5 | Y | 6 | 1 | 3.75 | 412.8446232 | 68.4 |
| 24360339 | -8.8 | | 4 | Y | 5 | 1 | 3.22 | 383.44244 | 63.99 |
| 24284989 | -8.8 | | 1 | Y | 3 | 0 | 3.59 | 279.33308 | 37.38 |
| 4263947 | -8.8 | | 5 | Y | 6 | 1 | 3.43 | 389.53812 | 126.24 |
| 7967963 | -8.8 | | 6 | Y | 7 | 2 | 2.4 | 441.54318 | 103.96 |
| 49720216 | -8.8 | | 6 | Y | 7 | 1 | 2.58 | 391.46458 | 128.98 |
| 4265184 | -8.8 | | 2 | Y | 6 | 0 | 3.2 | 336.36778 | 89.78 |
| 845411 | -8.8 | | 5 | Y | 7 | 1 | 2.15 | 402.46408 | 93.32 |
| 24286142 | -8.8 | | 6 | Y | 7 | 1 | 2.81 | 403.4273232 | 118.53 |
| 89855466 | -8.8 | | 1 | Y | 4 | 0 | 1.48 | 298.37948 | 41.9 |
| 89851280 | -8.8 | | 4 | Y | 6 | 1 | 3.08 | 387.43114 | 71.53 |
| 49648268 | -8.8 | | 2 | Y | 5 | 0 | 2.38 | 295.28942 | 55.84 |
| 99431270 | -8.8 | | 3 | Y | 6 | 2 | 2.7 | 358.39322 | 79.52 |
| 57266498 | -8.8 | | 5 | Y | 7 | 1 | 3.34 | 372.39504 | 106.87 |
| 24786695 | -8.8 | | 3 | Y | 7 | 3 | 1.52 | 317.31984 | 123.84 |
| 26657691 | -8.8 | | 4 | Y | 7 | 1 | 1.51 | 350.32486 | 97.97 |
| 14744570 | -8.8 | | 7 | Y | 7 | 1 | 3.16 | 366.36732 | 90.66 |
| 26616127 | -8.8 | | 5 | Y | 6 | 0 | 3.14 | 428.52612 | 68.21 |
| 26650210 | -8.8 | | 3 | Y | 6 | 2 | 2.22 | 346.44718 | 109.46 |
| 24373635 | -8.8 | | 3 | Y | 5 | 1 | 3.3 | 363.43288 | 91.71 |
| 853379 | -8.8 | | 4 | Y | 5 | 0 | 3.06 | 378.3914496 | 41.37 |
| 24342848 | -8.8 | | 2 | Y | 4 | 2 | 3.6 | 326.41272 | 90.2 |
| 22410697 | -8.8 | | 3 | Y | 5 | 1 | 3.76 | 350.75524 | 85.92 |
| 46501346 | -8.8 | | 6 | Y | 7 | 1 | 3.37 | 393.3166696 | 96.51 |
| 49667507 | -8.8 | | 8 | Y | 7 | 3 | 2.95 | 410.5276 | 123.78 |
| 56321007 | -8.8 | | 3 | Y | 5 | 2 | 3.85 | 404.50142 | 71.34 |
| 17505556 | -8.8 | | 4 | Y | 5 | 3 | 3.44 | 354.40452 | 80.9 |
| 22407971 | -8.8 | | 7 | Y | 6 | 0 | 3.05 | 378.42108 | 68.2 |
| 3715559 | -8.8 | | 5 | Y | 7 | 0 | 2.51 | 338.31416 | 83.68 |
| 49827558 | -8.8 | | 5 | Y | 6 | 1 | 3.48 | 385.47992 | 107.44 |
| 17407535 | -8.8 | | 6 | Y | 6 | 0 | 3.75 | 409.45496 | 85.47 |
| 22410150 | -8.8 | | 5 | Y | 5 | 1 | 3.99 | 414.54098 | 103.1 |
| 22400961 | -8.8 | | 5 | Y | 6 | 1 | 3.35 | 427.55966 | 78.1 |
| 56422732 | -8.8 | | 5 | Y | 5 | 1 | 3.92 | 375.50986 | 61.23 |
| 864728 | -8.8 | | 1 | Y | 6 | 1 | 1.89 | 315.36694 | 69.72 |
| 24804383 | -8.8 | | 3 | Y | 5 | 2 | 2.91 | 329.43658 | 61.44 |
| 17483943 | -8.8 | | 4 | Y | 7 | 0 | 3.75 | 416.279 | 94.82 |
| 124752957 | -8.8 | | 4 | Y | 7 | 1 | 1.51 | 384.47202 | 79.61 |
| 7969807 | -8.8 | | 4 | Y | 5 | 0 | 3.44 | 401.54552 | 105.8 |
| 24403124 | -8.8 | | 5 | Y | 7 | 0 | 2.74 | 400.49456 | 85.7 |
| 49675438 | -8.8 | | 2 | Y | 6 | 2 | 2.05 | 295.29272 | 93.84 |
| 47201190 | -8.8 | | 3 | Y | 6 | 3 | 1.19 | 317.3629 | 100.88 |
| 124949902 | -8.8 | | 3 | Y | 6 | 1 | 3.47 | 406.50068 | 91.65 |
| 49718292 | -8.8 | | 5 | Y | 7 | 1 | 2.07 | 378.47074 | 69.18 |
| 26533223 | -8.8 | | 2 | Y | 4 | 1 | 3.49 | 291.30072 | 55.23 |
| 17459470 | -8.8 | | 3 | Y | 6 | 1 | 2.55 | 366.43356 | 72.3 |
| 24358949 | -8.8 | | 4 | Y | 5 | 1 | 4 | 388.3862696 | 59.29 |
| 24403603 | -8.8 | | 3 | Y | 5 | 0 | 3.23 | 334.43318 | 71.79 |
| 24383997 | -8.8 | | 5 | Y | 6 | 1 | 2.91 | 434.50748 | 94.92 |
| 14744389 | -8.8 | | 3 | Y | 7 | 3 | 0.62 | 328.3657 | 109.15 |
| 26651585 | -8.8 | | 6 | Y | 4 | 1 | 3.71 | 388.50202 | 46.79 |
| 26527820 | -8.8 | | 3 | Y | 5 | 1 | 3.3 | 339.38504 | 56.79 |
| 124949290 | -8.8 | | 5 | Y | 6 | 1 | 2.33 | 414.53776 | 70.08 |
| 49674491 | -8.8 | | 6 | Y | 5 | 1 | 3.71 | 334.36852 | 68.29 |
| 26749333 | -8.8 | | 2 | Y | 7 | 2 | 2.52 | 342.29956 | 101.19 |
| 17486404 | -8.8 | | 4 | Y | 4 | 0 | 3.87 | 322.35452 | 56.51 |
| 24798843 | -8.8 | | 5 | Y | 7 | 0 | 2.05 | 380.48662 | 65.46 |
| 49817670 | -8.8 | | 4 | Y | 5 | 0 | 3.64 | 368.4494 | 83.81 |
| 24783691 | -8.8 | | 6 | Y | 5 | 1 | 3.74 | 401.88326 | 66.84 |
| 24384950 | -8.8 | | 4 | Y | 7 | 0 | 2.18 | 364.44416 | 66.63 |
| 24782278 | -8.8 | | 6 | Y | 4 | 1 | 3.65 | 404.4254296 | 50.61 |
| 24373964 | -8.8 | | 6 | Y | 7 | 1 | 3.94 | 448.4382296 | 77.75 |
| 104222587 | -8.8 | | 4 | Y | 5 | 1 | 3.23 | 332.35264 | 70.24 |
| 49818495 | -8.8 | | 5 | Y | 7 | 0 | 3.12 | 414.4053064 | 71.7 |
| 85199391 | -8.8 | | 7 | Y | 6 | 1 | 3.17 | 427.4917832 | 98.52 |
| 26648543 | -8.8 | | 3 | Y | 7 | 1 | 0.99 | 377.43962 | 83.88 |
| 24376488 | -8.8 | | 6 | Y | 7 | 3 | 3.59 | 400.4299 | 96.37 |
| 26726618 | -8.8 | | 5 | Y | 6 | 1 | 3.53 | 372.82872 | 111.56 |
| 24385666 | -8.8 | | 6 | Y | 7 | 1 | 2.7 | 389.45032 | 81.41 |
| 14740849 | -8.8 | | 3 | Y | 5 | 0 | 3.96 | 373.44762 | 55.2 |
| 14720497 | -8.8 | | 5 | Y | 7 | 2 | 3.22 | 426.53184 | 113 |
| 17413860 | -8.8 | | 5 | Y | 5 | 1 | 3.93 | 375.44358 | 96.26 |
| 24356101 | -8.8 | | 5 | Y | 7 | 1 | 3.06 | 369.3944 | 115.42 |
| 17431512 | -8.8 | | 2 | Y | 5 | 0 | 3.52 | 330.33676 | 60.66 |
| 26532009 | -8.8 | | 3 | Y | 5 | 0 | 3.01 | 371.45004 | 64.22 |
| 26614122 | -8.8 | | 2 | Y | 6 | 1 | 1.05 | 322.36112 | 70.73 |
| 24827615 | -8.8 | | 4 | Y | 6 | 0 | 3.1 | 349.2641096 | 80.71 |
| 124349974 | -8.8 | | 0 | Y | 3 | 2 | 2.46 | 263.29062 | 49.33 |
| 17479001 | -8.8 | | 9 | Y | 6 | 2 | 3.61 | 418.48494 | 76.66 |
| 24385362 | -8.8 | | 4 | Y | 6 | 1 | 3.79 | 393.8694 | 72.18 |
| 24394898 | -8.8 | | 4 | Y | 5 | 1 | 3.89 | 395.4499232 | 92.23 |
| 57263236 | -8.8 | | 6 | Y | 5 | 1 | 2.8 | 391.50596 | 53.85 |
| 24794758 | -8.8 | | 3 | Y | 6 | 1 | 3.18 | 416.26852 | 67.6 |
| 49714598 | -8.8 | | 4 | Y | 7 | 0 | 3.09 | 445.57494 | 88.07 |
| 87341082 | -8.8 | | 6 | Y | 7 | 2 | 2.56 | 413.3709696 | 105.24 |
| 24329371 | -8.8 | | 2 | Y | 5 | 0 | 3.44 | 353.80224 | 55.2 |
| 26662811 | -8.8 | | 7 | Y | 5 | 0 | 3.31 | 411.58018 | 45.06 |
| 3715941 | -8.8 | | 3 | Y | 7 | 1 | 2.4 | 340.3764 | 91.9 |
| 24831778 | -8.8 | | 4 | Y | 4 | 2 | 3.98 | 338.42342 | 90.51 |
| 26662345 | -8.8 | | 3 | Y | 7 | 2 | 1.47 | 346.33946 | 110.48 |
| 49673836 | -8.8 | | 6 | Y | 7 | 2 | 2.95 | 407.3432496 | 94.06 |
| 24341743 | -8.8 | | 4 | Y | 7 | 0 | 2.87 | 297.26554 | 93.97 |
| 865707 | -8.8 | | 0 | Y | 5 | 0 | 3.25 | 315.36364 | 55.84 |
| 14745246 | -8.8 | | 7 | Y | 5 | 2 | 3.25 | 398.4505632 | 83.65 |
| 17514736 | -8.8 | | 5 | Y | 5 | 0 | 3.47 | 390.50128 | 74.63 |
| 17385613 | -8.8 | | 6 | Y | 5 | 1 | 3.04 | 352.42686 | 58.64 |
| 24397568 | -8.8 | | 5 | Y | 7 | 1 | 2.84 | 419.3986896 | 129.96 |
| 103076612 | -8.8 | | 3 | Y | 4 | 0 | 3.24 | 394.89392 | 40.62 |
| 24359450 | -8.8 | | 5 | Y | 5 | 1 | 3.84 | 397.46902 | 61.77 |
| 865741 | -8.8 | | 3 | Y | 7 | 1 | 1.8 | 298.29666 | 93.71 |
| 4265144 | -8.8 | | 2 | Y | 4 | 0 | 3.3 | 332.3957 | 42.43 |
| 124948608 | -8.8 | | 5 | Y | 6 | 0 | 3.24 | 409.54768 | 83.7 |
| 17408286 | -8.8 | | 5 | Y | 5 | 0 | 3.12 | 407.4094496 | 91.52 |
| 24828920 | -8.8 | | 3 | Y | 5 | 0 | 2.86 | 360.1589 | 69.4 |
| 17387185 | -8.8 | | 7 | Y | 6 | 1 | 3.96 | 437.55448 | 117.38 |
| 17513446 | -8.8 | | 5 | Y | 6 | 1 | 3.55 | 440.3832992 | 68.39 |
| 89855567 | -8.8 | | 3 | Y | 6 | 1 | 3.9 | 380.78122 | 86.36 |
| 26646476 | -8.8 | | 4 | Y | 7 | 1 | 3.17 | 375.37738 | 98.23 |
| 14743127 | -8.8 | | 4 | Y | 7 | 2 | 3.24 | 361.39716 | 81.4 |
| 4256132 | -8.8 | | 6 | Y | 7 | 1 | 3.28 | 337.37576 | 81.93 |
| 17447316 | -8.8 | | 3 | Y | 5 | 1 | 3.95 | 322.35782 | 74.92 |
| 24366405 | -8.8 | | 2 | Y | 6 | 3 | 4 | 385.4417 | 125.79 |
| 24290975 | -8.8 | | 5 | Y | 6 | 1 | 3.63 | 376.43164 | 109.97 |
| 49645725 | -8.8 | | 5 | Y | 5 | 1 | 3.18 | 394.4387232 | 58.64 |
| 24797872 | -8.8 | | 5 | Y | 7 | 1 | 2.19 | 354.40298 | 74.6 |
| 17505078 | -8.8 | | 2 | Y | 5 | 0 | 2.83 | 318.37242 | 60.67 |
| 49734389 | -8.8 | | 7 | Y | 7 | 2 | 2.35 | 435.58012 | 103.96 |
| 17471806 | -8.8 | | 4 | Y | 5 | 4 | 2.94 | 313.3262432 | 84.22 |
| 17474140 | -8.8 | | 4 | Y | 4 | 0 | 3.81 | 304.34254 | 52.08 |
| 24800360 | -8.8 | | 8 | Y | 6 | 0 | 3.36 | 428.59078 | 94.64 |
| 17474334 | -8.8 | | 4 | Y | 5 | 0 | 3.42 | 361.3178696 | 56.49 |
| 862350 | -8.7 | | 2 | Y | 7 | 0 | 3.12 | 327.34272 | 73.79 |
| 26637629 | -8.7 | | 2 | Y | 4 | 0 | 3.38 | 352.45 | 70.67 |
| 22405065 | -8.7 | | 6 | Y | 6 | 1 | 3.11 | 409.50132 | 92.89 |
| 3711741 | -8.7 | | 2 | Y | 7 | 0 | 0.35 | 347.36896 | 125.44 |
| 24823704 | -8.7 | | 2 | Y | 6 | 0 | 2.11 | 338.40358 | 60.13 |
| 4246252 | -8.7 | | 4 | Y | 7 | 2 | 2.63 | 351.35598 | 85.35 |
| 17408581 | -8.7 | | 2 | Y | 7 | 4 | 3.41 | 369.3728832 | 148.29 |
| 3715001 | -8.7 | | 3 | Y | 7 | 0 | 3.28 | 384.1869 | 86.7 |
| 24304858 | -8.7 | | 4 | Y | 5 | 1 | 3.66 | 332.35264 | 64.24 |
| 24827268 | -8.7 | | 5 | Y | 7 | 1 | 2.79 | 365.38256 | 82.45 |
| 24819795 | -8.7 | | 4 | Y | 4 | 1 | 3.23 | 331.36458 | 60.44 |
| 124948138 | -8.7 | | 4 | Y | 7 | 1 | 1.51 | 400.85874 | 84.42 |
| 26616268 | -8.7 | | 4 | Y | 7 | 0 | 2.17 | 390.48144 | 76.8 |
| 26624910 | -8.7 | | 4 | Y | 6 | 3 | 4 | 396.4379832 | 125.19 |
| 22411151 | -8.7 | | 7 | Y | 7 | 1 | 3.27 | 434.4811232 | 104.73 |
| 99359814 | -8.7 | | 0 | Y | 4 | 0 | 2.1 | 292.33184 | 40.62 |
| 26650682 | -8.7 | | 7 | Y | 6 | 1 | 3.95 | 407.50536 | 60.95 |
| 17410015 | -8.7 | | 5 | Y | 4 | 0 | 3.14 | 386.4829232 | 50.69 |
| 3712175 | -8.7 | | 1 | Y | 6 | 0 | 1.59 | 248.24282 | 68.86 |
| 49825647 | -8.7 | | 3 | Y | 6 | 1 | 3.28 | 371.38868 | 79.37 |
| 24411853 | -8.7 | | 4 | Y | 6 | 1 | 2.27 | 350.36792 | 67.87 |
| 14725100 | -8.7 | | 4 | Y | 6 | 0 | 2.7 | 391.4629 | 60.93 |
| 26613863 | -8.7 | | 2 | Y | 4 | 1 | 2.78 | 356.48176 | 81.67 |
| 844137 | -8.7 | | 4 | Y | 7 | 2 | 3.7 | 382.48258 | 114.71 |
| 24362486 | -8.7 | | 5 | Y | 6 | 0 | 2.59 | 325.3154 | 78.63 |
| 24800100 | -8.7 | | 4 | Y | 5 | 1 | 3.14 | 374.47874 | 51.36 |
| 7965659 | -8.7 | | 5 | Y | 7 | 0 | 1.11 | 359.4195 | 70.16 |
| 17468721 | -8.7 | | 3 | Y | 6 | 0 | 3.12 | 335.3732832 | 69.37 |
| 24345308 | -8.7 | | 5 | Y | 5 | 1 | 3.65 | 350.41098 | 74.92 |
| 4263021 | -8.7 | | 3 | Y | 6 | 1 | 3.63 | 313.31284 | 90.86 |
| 24372084 | -8.7 | | 3 | Y | 5 | 0 | 2.62 | 325.40482 | 55.2 |
| 24392280 | -8.7 | | 3 | Y | 5 | 0 | 3.5 | 424.29052 | 48.93 |
| 74373760 | -8.7 | | 6 | Y | 6 | 1 | 2.31 | 322.31476 | 89.12 |
| 26660336 | -8.7 | | 5 | Y | 5 | 1 | 2.96 | 323.36902 | 104.1 |
| 24805558 | -8.7 | | 2 | Y | 4 | 1 | 3.98 | 377.23732 | 49.97 |
| 49820346 | -8.7 | | 5 | Y | 6 | 1 | 2.34 | 352.3838 | 81.43 |
| 14744348 | -8.7 | | 7 | Y | 5 | 1 | 3.54 | 403.49674 | 86.6 |
| 4242892 | -8.7 | | 3 | Y | 5 | 1 | 2.73 | 337.3476432 | 58.1 |
| 49669376 | -8.7 | | 3 | Y | 5 | 0 | 3.16 | 360.45054 | 100.03 |
| 26616238 | -8.7 | | 3 | Y | 5 | 0 | 3.68 | 354.42282 | 91.65 |
| 26648175 | -8.7 | | 3 | Y | 4 | 1 | 2.89 | 352.4020432 | 49.41 |
| 26660380 | -8.7 | | 0 | Y | 3 | 0 | 2.75 | 283.73228 | 25.88 |
| 17481612 | -8.7 | | 4 | Y | 4 | 0 | 3.98 | 322.35452 | 56.51 |
| 49666326 | -8.7 | | 4 | Y | 5 | 1 | 2.26 | 306.31536 | 84.23 |
| 7965294 | -8.7 | | 6 | Y | 5 | 1 | 3.04 | 418.50808 | 85.69 |
| 16952870 | -8.7 | | 2 | Y | 5 | 1 | 2.76 | 353.41162 | 64.63 |
| 49673053 | -8.7 | | 5 | Y | 5 | 0 | 3.83 | 321.3267 | 69.4 |
| 17481680 | -8.7 | | 2 | Y | 4 | 0 | 2.77 | 320.385 | 40.62 |
| 26532719 | -8.7 | | 6 | Y | 7 | 1 | 3.65 | 415.51242 | 175.58 |
| 26731517 | -8.7 | | 4 | Y | 7 | 2 | 2.18 | 372.39504 | 113.86 |
| 26621054 | -8.7 | | 5 | Y | 7 | 2 | 2.28 | 417.45712 | 105.66 |
| 24797018 | -8.7 | | 3 | Y | 7 | 1 | 2.76 | 300.26618 | 93.38 |
| 26648540 | -8.7 | | 3 | Y | 7 | 1 | -0.17 | 337.37576 | 83.88 |
| 56322652 | -8.7 | | 3 | Y | 6 | 0 | 2.07 | 308.28818 | 69.9 |
| 22401102 | -8.7 | | 4 | Y | 7 | 2 | 2.55 | 351.35598 | 96.32 |
| 49820145 | -8.7 | | 9 | Y | 6 | 1 | 3.16 | 419.51606 | 69.72 |
| 103074946 | -8.7 | | 2 | Y | 5 | 0 | 2.7 | 320.3883 | 40.85 |
| 49721022 | -8.7 | | 6 | Y | 6 | 1 | 1.46 | 394.3908496 | 69.3 |
| 103050207 | -8.7 | | 6 | Y | 7 | 1 | 2.38 | 365.38256 | 92.36 |
| 85271706 | -8.7 | | 6 | Y | 7 | 2 | 2.01 | 359.4195 | 91.31 |
| 24369659 | -8.7 | | 3 | Y | 7 | 1 | 3.2 | 371.41028 | 112.69 |
| 24794149 | -8.7 | | 2 | Y | 6 | 1 | 2.76 | 333.3407 | 83.98 |
| 26645342 | -8.7 | | 7 | Y | 7 | 1 | 2.28 | 434.5307 | 83.77 |
| 49731079 | -8.7 | | 6 | Y | 7 | 1 | 2.83 | 415.55226 | 101.08 |
| 50102180 | -8.7 | | 4 | Y | 7 | 1 | 3.26 | 335.35988 | 74.42 |
| 24792133 | -8.7 | | 6 | Y | 6 | 0 | 2.63 | 337.3261 | 80.75 |
| 7972667 | -8.7 | | 3 | Y | 6 | 3 | 3.95 | 344.36664 | 100.86 |
| 49818460 | -8.7 | | 5 | Y | 6 | 0 | 3.17 | 383.48396 | 60.01 |
| 124948285 | -8.7 | | 6 | Y | 7 | 2 | 2.51 | 447.5046432 | 84.03 |
| 17437606 | -8.7 | | 3 | Y | 6 | 1 | 2.26 | 323.39224 | 66.81 |
| 26541161 | -8.7 | | 6 | Y | 5 | 0 | 3.79 | 412.8165896 | 85.97 |
| 858358 | -8.7 | | 3 | Y | 3 | 1 | 3.73 | 304.3856 | 36.1 |
| 17504224 | -8.7 | | 1 | Y | 4 | 3 | 3.34 | 354.46588 | 85.61 |
| 24295278 | -8.7 | | 0 | Y | 3 | 0 | 3.8 | 250.2488 | 43.35 |
| 144089286 | -8.7 | | 3 | Y | 4 | 2 | 3.95 | 316.3699432 | 54.12 |
| 26726003 | -8.7 | | 4 | Y | 7 | 1 | 3.26 | 336.29828 | 105.99 |
| 26646724 | -8.7 | | 5 | Y | 7 | 1 | 2.98 | 393.43572 | 98.23 |
| 26651332 | -8.7 | | 5 | Y | 6 | 0 | 2.78 | 410.53244 | 66.82 |
| 845013 | -8.7 | | 5 | Y | 6 | 0 | 3.26 | 401.4545032 | 84.68 |
| 14723320 | -8.7 | | 4 | Y | 7 | 0 | 1.29 | 383.48564 | 94.76 |
| 3716387 | -8.7 | | 4 | Y | 5 | 1 | 2.64 | 322.35782 | 64.36 |
| 853269 | -8.7 | | 3 | Y | 6 | 0 | 2.66 | 325.3882 | 92.15 |
| 26727504 | -8.7 | | 5 | Y | 7 | 1 | 2.53 | 427.47024 | 123.41 |
| 847042 | -8.7 | | 4 | Y | 7 | 0 | 3.95 | 427.92714 | 77.05 |
| 49722262 | -8.7 | | 7 | Y | 7 | 1 | 2.03 | 436.90914 | 101.16 |
| 24799579 | -8.7 | | 7 | Y | 6 | 1 | 3.41 | 413.4172464 | 68.8 |
| 846875 | -8.7 | | 7 | Y | 7 | 0 | 2.99 | 385.43686 | 104.68 |
| 24805043 | -8.7 | | 4 | Y | 7 | 1 | 3.89 | 447.90864 | 114.1 |
| 3714331 | -8.7 | | 3 | Y | 5 | 0 | 3.69 | 402.3664896 | 53.76 |
| 7974316 | -8.7 | | 3 | Y | 6 | 1 | 3.39 | 323.3243632 | 65.19 |
| 56321307 | -8.7 | | 6 | Y | 6 | 0 | 2.8 | 448.59872 | 111.38 |
| 24331969 | -8.7 | | 4 | Y | 7 | 2 | 2.36 | 346.72194 | 112.22 |
| 57260932 | -8.7 | | 5 | Y | 6 | 2 | 3.1 | 391.4629 | 82.08 |
| 49677036 | -8.7 | | 2 | Y | 5 | 1 | 2.56 | 302.3019 | 92.96 |
| 17433960 | -8.7 | | 5 | Y | 5 | 1 | 3.76 | 381.4713 | 125.49 |
| 24829054 | -8.7 | | 4 | Y | 6 | 1 | 3.24 | 375.4668 | 53.4 |
| 57266035 | -8.7 | | 4 | Y | 7 | 1 | 1.27 | 322.3148432 | 113.63 |
| 57255615 | -8.7 | | 5 | Y | 7 | 1 | 1.79 | 432.4620264 | 109.54 |
| 26649715 | -8.7 | | 3 | Y | 7 | 1 | 0.9 | 367.4448 | 72.54 |
| 22407979 | -8.7 | | 6 | Y | 6 | 1 | 3.83 | 393.43242 | 85.61 |
| 4248217 | -8.7 | | 1 | Y | 4 | 0 | 2.99 | 266.2482 | 48.67 |
| 49713969 | -8.7 | | 5 | Y | 6 | 0 | 3.66 | 348.2793496 | 76.53 |
| 131500240 | -8.7 | | 5 | Y | 7 | 1 | 2.19 | 407.44238 | 108.39 |
| 49731385 | -8.7 | | 5 | Y | 6 | 0 | 3.02 | 429.9198 | 85.86 |
| 7976218 | -8.7 | | 3 | Y | 5 | 2 | 3.84 | 390.1552496 | 69.98 |
| 24787915 | -8.7 | | 4 | Y | 7 | 1 | 2.91 | 314.29276 | 93.38 |
| 49671043 | -8.7 | | 2 | Y | 7 | 0 | 1.7 | 357.3357432 | 88.13 |
| 24354745 | -8.7 | | 3 | Y | 4 | 2 | 3.86 | 360.51352 | 71.16 |
| 26730586 | -8.7 | | 5 | Y | 7 | 1 | 2.25 | 375.37738 | 92.51 |
| 3713452 | -8.7 | | 2 | Y | 5 | 0 | 1.96 | 335.3269496 | 59.43 |
| 7968967 | -8.7 | | 5 | Y | 6 | 0 | 3.09 | 410.48608 | 88.86 |
| 4250233 | -8.7 | | 4 | Y | 7 | 1 | 2.68 | 411.47414 | 104.46 |
| 57261321 | -8.7 | | 5 | Y | 7 | 2 | 2.34 | 371.4302 | 99.15 |
| 24708026 | -8.7 | | 6 | Y | 6 | 1 | 2.54 | 365.42562 | 71.53 |
| 24799739 | -8.7 | | 7 | Y | 7 | 1 | 2.69 | 402.49214 | 67.46 |
| 57261327 | -8.7 | | 7 | Y | 5 | 1 | 3.85 | 392.4228432 | 58.64 |
| 17508117 | -8.7 | | 5 | Y | 6 | 1 | 3.32 | 422.52322 | 110.56 |
| 866070 | -8.7 | | 4 | Y | 7 | 1 | 3.54 | 403.4769 | 85.96 |
| 24362655 | -8.7 | | 2 | Y | 5 | 0 | 2.74 | 366.45344 | 57.69 |
| 847024 | -8.7 | | 6 | Y | 6 | 2 | 3.95 | 435.4394696 | 74.58 |
| 26658304 | -8.7 | | 5 | Y | 7 | 1 | 3.36 | 366.39376 | 119.2 |
| 144098240 | -8.7 | | 4 | Y | 6 | 0 | 1.82 | 380.4154432 | 58.44 |
| 49649376 | -8.7 | | 4 | Y | 4 | 1 | 3.83 | 326.7769 | 55.13 |
| 17457303 | -8.7 | | 2 | Y | 7 | 3 | 2.99 | 367.39844 | 104.05 |
| 7968635 | -8.7 | | 5 | Y | 6 | 0 | 3.05 | 411.47084 | 85.2 |
| 24340797 | -8.7 | | 4 | Y | 5 | 3 | 3.01 | 325.40482 | 78.01 |
| 124948339 | -8.7 | | 4 | Y | 5 | 1 | 3.22 | 383.8034032 | 64.11 |
| 56323404 | -8.7 | | 6 | Y | 6 | 1 | 3.58 | 402.87132 | 67.87 |
| 24799625 | -8.7 | | 4 | Y | 4 | 0 | 3.19 | 376.4202264 | 75.57 |
| 26614842 | -8.7 | | 3 | Y | 7 | 0 | 2.87 | 378.37802 | 70.12 |
| 49719169 | -8.7 | | 7 | Y | 4 | 0 | 3.44 | 382.53896 | 40.62 |
| 24812561 | -8.7 | | 2 | Y | 5 | 1 | 3.19 | 308.33124 | 74.92 |
| 856357 | -8.7 | | 1 | Y | 6 | 0 | 3.61 | 343.38188 | 65.08 |
| 124753229 | -8.7 | | 8 | Y | 7 | 1 | 2.18 | 447.61074 | 78.95 |
| 26615270 | -8.7 | | 4 | Y | 6 | 0 | 1.36 | 359.42434 | 71.87 |
| 26730314 | -8.7 | | 3 | Y | 5 | 0 | 3.98 | 330.33676 | 64.37 |
| 26661255 | -8.7 | | 3 | Y | 7 | 2 | 3.16 | 394.76474 | 111.8 |
| 24316123 | -8.7 | | 3 | Y | 7 | 1 | 3.69 | 337.3078832 | 88.56 |
| 26731541 | -8.7 | | 2 | Y | 5 | 2 | 2.84 | 283.27872 | 75.63 |
| 124949700 | -8.7 | | 5 | Y | 7 | 1 | 3.62 | 439.509 | 81.93 |
| 24818174 | -8.7 | | 3 | Y | 5 | 0 | 3.19 | 319.35718 | 48.53 |
| 49666921 | -8.7 | | 1 | Y | 3 | 0 | 3.94 | 262.2595 | 43.35 |
| 49718462 | -8.7 | | 6 | Y | 6 | 0 | 3.26 | 378.84992 | 64.8 |
| 24308998 | -8.7 | | 4 | Y | 6 | 1 | 2.76 | 377.86516 | 75.17 |
| 24357057 | -8.7 | | 3 | Y | 6 | 1 | 3.77 | 346.33616 | 98.84 |
| 49671678 | -8.7 | | 3 | Y | 3 | 0 | 3.95 | 321.41282 | 37.38 |
| 24787620 | -8.7 | | 2 | Y | 5 | 2 | 2.35 | 314.35896 | 88.85 |
| 49713933 | -8.7 | | 7 | Y | 4 | 1 | 3.98 | 398.49684 | 49.41 |
| 81066170 | -8.7 | | 2 | Y | 5 | 1 | 3.58 | 400.3522064 | 99.72 |
| 26730892 | -8.7 | | 4 | Y | 6 | 1 | 3.49 | 346.38252 | 72.68 |
| 24366290 | -8.7 | | 6 | Y | 7 | 1 | 2.97 | 438.92502 | 93.32 |
| 26649891 | -8.7 | | 4 | Y | 5 | 1 | 3.38 | 367.46464 | 92.23 |
| 17402806 | -8.7 | | 4 | Y | 5 | 0 | 3.7 | 403.3776896 | 72.71 |
| 26530481 | -8.7 | | 3 | Y | 6 | 0 | 2.48 | 382.3801496 | 66.4 |
| 49822668 | -8.7 | | 4 | Y | 6 | 2 | 2.99 | 372.45802 | 85.15 |
| 4248161 | -8.7 | | 2 | Y | 5 | 1 | 3.96 | 336.3844 | 72.2 |
| 14742896 | -8.7 | | 4 | Y | 6 | 1 | 3.72 | 352.43016 | 69.04 |
| 26617419 | -8.7 | | 6 | Y | 6 | 1 | 3.54 | 354.3781632 | 76.88 |
| 124894489 | -8.7 | | 5 | Y | 6 | 2 | 3.41 | 406.8384632 | 95.01 |
| 24349558 | -8.7 | | 6 | Y | 5 | 1 | 3.92 | 393.50192 | 92.23 |
| 26535564 | -8.7 | | 3 | Y | 5 | 0 | 3.06 | 376.4450432 | 58.23 |
| 49671239 | -8.7 | | 5 | Y | 4 | 0 | 3.06 | 346.3711064 | 32.78 |
| 99360885 | -8.6 | | 2 | Y | 4 | 0 | 3.96 | 290.31596 | 53.33 |
| 26537660 | -8.6 | | 3 | Y | 6 | 0 | 2.64 | 356.37572 | 125.36 |
| 24331480 | -8.6 | | 2 | Y | 5 | 0 | 3.86 | 348.35688 | 82.47 |
| 3714742 | -8.6 | | 3 | Y | 6 | 1 | 3.2 | 430.99728 | 76.92 |
| 137276044 | -8.6 | | 4 | Y | 7 | 4 | 3.49 | 389.23866 | 105.82 |
| 99456145 | -8.6 | | 6 | Y | 7 | 1 | 2.43 | 430.3981896 | 108.64 |
| 4241680 | -8.6 | | 4 | Y | 6 | 1 | 3.02 | 372.45802 | 67.87 |
| 26540828 | -8.6 | | 3 | Y | 5 | 0 | 3.13 | 326.36966 | 69.57 |
| 24409111 | -8.6 | | 5 | Y | 5 | 1 | 3.33 | 342.45848 | 88.87 |
| 863511 | -8.6 | | 3 | Y | 6 | 0 | 3.12 | 356.442 | 75.95 |
| 56316297 | -8.6 | | 6 | Y | 7 | 0 | 3.41 | 447.3328 | 98.09 |
| 85198962 | -8.6 | | 4 | Y | 5 | 0 | 3.62 | 326.38958 | 59.5 |
| 124948501 | -8.6 | | 5 | Y | 7 | 0 | 0.7 | 420.50412 | 73.82 |
| 57263990 | -8.6 | | 8 | Y | 7 | 1 | 3 | 430.88142 | 84.94 |
| 26651438 | -8.6 | | 5 | Y | 5 | 0 | 3.35 | 379.4273832 | 47.36 |
| 24345745 | -8.6 | | 4 | Y | 6 | 4 | 1.11 | 394.59448 | 93.66 |
| 49665819 | -8.6 | | 3 | Y | 5 | 0 | 1.99 | 345.8233 | 45.55 |
| 99357644 | -8.6 | | 2 | Y | 4 | 0 | 2.73 | 298.35956 | 67.24 |
| 3717476 | -8.6 | | 6 | Y | 6 | 0 | 3.71 | 379.40584 | 68.98 |
| 22404240 | -8.6 | | 3 | Y | 4 | 1 | 3.72 | 292.28548 | 70.34 |
| 46371155 | -8.6 | | 8 | Y | 7 | 0 | 3.79 | 418.44188 | 83.44 |
| 174006469 | -8.6 | | 2 | Y | 5 | 1 | 3.02 | 333.36384 | 97.25 |
| 99360405 | -8.6 | | 4 | Y | 5 | 1 | 2.98 | 363.3849232 | 67.87 |
| 144206751 | -8.6 | | 2 | Y | 4 | 1 | 3.17 | 293.36296 | 50.68 |
| 92092845 | -8.6 | | 2 | Y | 5 | 1 | 2.94 | 311.37824 | 58.1 |
| 24333149 | -8.6 | | 4 | Y | 7 | 1 | 2.79 | 413.4701 | 167.73 |
| 144096586 | -8.6 | | 5 | Y | 6 | 0 | 1.07 | 374.4739 | 66.92 |
| 24831103 | -8.6 | | 4 | Y | 5 | 1 | 1.77 | 338.40028 | 66.48 |
| 124896557 | -8.6 | | 4 | Y | 5 | 4 | 3.19 | 334.2958296 | 89.52 |
| 17450476 | -8.6 | | 5 | Y | 5 | 3 | 1.88 | 322.35782 | 82.19 |
| 26644574 | -8.6 | | 4 | Y | 6 | 1 | 1.75 | 360.4274 | 84.09 |
| 24339393 | -8.6 | | 1 | Y | 4 | 1 | 2.73 | 307.34318 | 60.44 |
| 864349 | -8.6 | | 2 | Y | 4 | 0 | 3.83 | 304.3889 | 41.91 |
| 49672532 | -8.6 | | 6 | Y | 6 | 0 | 3.48 | 368.3799 | 74.97 |
| 24391613 | -8.6 | | 6 | Y | 6 | 2 | 2.72 | 340.3515832 | 80.04 |
| 26657827 | -8.6 | | 5 | Y | 6 | 1 | 2.94 | 419.54088 | 150.94 |
| 49826761 | -8.6 | | 3 | Y | 7 | 1 | 1.08 | 383.48726 | 78.43 |
| 7966415 | -8.6 | | 3 | Y | 7 | 0 | 1.67 | 356.41886 | 71.58 |
| 7972863 | -8.6 | | 6 | Y | 5 | 2 | 3.69 | 387.42784 | 77.84 |
| 49818052 | -8.6 | | 6 | Y | 4 | 1 | 3.8 | 402.8383896 | 49.41 |
| 4261238 | -8.6 | | 4 | Y | 5 | 1 | 3.57 | 336.33804 | 79.57 |
| 22416115 | -8.6 | | 1 | Y | 4 | 0 | 3.23 | 313.39082 | 46.61 |
| 49817331 | -8.6 | | 7 | Y | 7 | 2 | 3.1 | 395.40524 | 108.67 |
| 861197 | -8.6 | | 3 | Y | 5 | 0 | 3.92 | 322.38426 | 80.55 |
| 49648589 | -8.6 | | 4 | Y | 5 | 0 | 3.29 | 296.27418 | 65.74 |
| 50085772 | -8.6 | | 2 | Y | 5 | 2 | 0.97 | 310.39348 | 65.6 |
| 57268044 | -8.6 | | 9 | Y | 6 | 2 | 3.99 | 438.53924 | 92.88 |
| 4242876 | -8.6 | | 2 | Y | 3 | 1 | 3.49 | 275.30132 | 42.24 |
| 842529 | -8.6 | | 5 | Y | 7 | 1 | 2.77 | 364.3978 | 78.27 |
| 856421 | -8.6 | | 4 | Y | 6 | 0 | 3.26 | 385.41526 | 83.17 |
| 50112960 | -8.6 | | 5 | Y | 6 | 1 | 3.29 | 417.92902 | 63.57 |
| 49736019 | -8.6 | | 5 | Y | 6 | 1 | 3.38 | 357.42676 | 97.04 |
| 24305009 | -8.6 | | 5 | Y | 7 | 0 | 3.54 | 411.83832 | 77.57 |
| 24296661 | -8.6 | | 4 | Y | 5 | 1 | 2.81 | 345.3481 | 76.37 |
| 26636086 | -8.6 | | 5 | Y | 6 | 1 | 2.78 | 378.42108 | 71.63 |
| 26614860 | -8.6 | | 4 | Y | 6 | 1 | 2.5 | 313.2831832 | 77.25 |
| 24780724 | -8.6 | | 2 | Y | 4 | 0 | 3.35 | 304.34254 | 46.34 |
| 4256104 | -8.6 | | 5 | Y | 6 | 1 | 2.44 | 350.36792 | 69.56 |
| 49735418 | -8.6 | | 2 | Y | 6 | 0 | 2.1 | 324.377 | 59.61 |
| 56436737 | -8.6 | | 5 | Y | 6 | 0 | 3.21 | 381.8091232 | 84.68 |
| 144098907 | -8.6 | | 9 | Y | 7 | 1 | 1.97 | 440.53198 | 84.94 |
| 51090740 | -8.6 | | 3 | Y | 7 | 1 | 1.52 | 345.373 | 104.82 |
| 24790624 | -8.6 | | 1 | Y | 4 | 2 | 3.56 | 299.32602 | 71.93 |
| 24786325 | -8.6 | | 6 | Y | 6 | 0 | 3.24 | 396.50586 | 100.36 |
| 3714726 | -8.6 | | 4 | Y | 7 | 1 | 2.19 | 354.35662 | 92.42 |
| 852669 | -8.6 | | 8 | Y | 7 | 0 | 2.76 | 399.46344 | 104.68 |
| 11114454 | -8.6 | | 5 | Y | 7 | 0 | 3.01 | 406.5239 | 60.51 |
| 26659066 | -8.6 | | 2 | Y | 6 | 0 | 1.99 | 307.30342 | 71.86 |
| 3716172 | -8.6 | | 7 | Y | 7 | 1 | 2.82 | 394.48514 | 101.16 |
| 24779126 | -8.6 | | 5 | Y | 6 | 1 | 3.55 | 414.51784 | 84.09 |
| 861209 | -8.6 | | 6 | Y | 6 | 0 | 3.76 | 352.41024 | 89.78 |
| 14737501 | -8.6 | | 6 | Y | 7 | 1 | 2.41 | 405.51108 | 100.89 |
| 17475843 | -8.6 | | 3 | Y | 5 | 1 | 2.53 | 308.33124 | 66.48 |
| 24353556 | -8.6 | | 2 | Y | 6 | 1 | 2.02 | 342.38898 | 75.19 |
| 17387799 | -8.6 | | 3 | Y | 5 | 1 | 2.87 | 328.45182 | 59.81 |
| 7974050 | -8.6 | | 6 | Y | 6 | 1 | 3.12 | 350.41428 | 69.04 |
| 49730781 | -8.6 | | 5 | Y | 5 | 0 | 3.77 | 371.45004 | 73.75 |
| 4242517 | -8.6 | | 5 | Y | 7 | 0 | 2.36 | 423.89538 | 71.33 |
| 49680611 | -8.6 | | 4 | Y | 6 | 1 | 3.06 | 363.38982 | 116.15 |
| 14728350 | -8.6 | | 3 | Y | 7 | 2 | 3.27 | 377.3205696 | 92.28 |
| 49648636 | -8.6 | | 2 | Y | 6 | 0 | 2.38 | 356.39564 | 83.14 |
| 99360735 | -8.6 | | 3 | Y | 3 | 0 | 3.77 | 334.40828 | 35.53 |
| 124948647 | -8.6 | | 5 | Y | 6 | 1 | 3.8 | 381.49452 | 99.25 |
| 24364042 | -8.6 | | 4 | Y | 6 | 1 | 3.71 | 399.48658 | 126.88 |
| 49731025 | -8.6 | | 4 | Y | 7 | 2 | 2.13 | 396.2159 | 117.01 |
| 24817125 | -8.6 | | 5 | Y | 7 | 0 | 3.06 | 408.85598 | 108.65 |
| 26540118 | -8.6 | | 3 | Y | 5 | 1 | 2.74 | 358.38992 | 74.92 |
| 49723377 | -8.6 | | 4 | Y | 5 | 1 | 3.72 | 358.81876 | 62.55 |
| 87336541 | -8.6 | | 2 | Y | 5 | 0 | 2.53 | 341.79154 | 61.42 |
| 26649563 | -8.6 | | 4 | Y | 7 | 1 | 3.28 | 352.3871 | 90.02 |
| 24786926 | -8.6 | | 6 | Y | 6 | 1 | 3.63 | 340.32674 | 85.97 |
| 17504596 | -8.6 | | 6 | Y | 6 | 1 | 3.74 | 298.29336 | 84.15 |
| 56317004 | -8.6 | | 6 | Y | 5 | 2 | 3.79 | 393.8661 | 71.09 |
| 17439645 | -8.6 | | 7 | Y | 7 | 2 | 3.85 | 436.52666 | 113 |
| 49718495 | -8.6 | | 4 | Y | 5 | 0 | 3.08 | 361.43692 | 55.2 |
| 24406344 | -8.6 | | 6 | Y | 7 | 0 | 3.25 | 447.59082 | 80.23 |
| 24405414 | -8.6 | | 4 | Y | 6 | 1 | 2.61 | 402.4209632 | 76.88 |
| 49818734 | -8.6 | | 4 | Y | 6 | 0 | 2.76 | 355.4308 | 60.01 |
| 14739318 | -8.6 | | 3 | Y | 5 | 2 | 3.65 | 369.4606 | 115.62 |
| 4241624 | -8.6 | | 7 | Y | 7 | 2 | 2.51 | 443.4944 | 87.57 |
| 74373959 | -8.6 | | 5 | Y | 6 | 3 | 2.96 | 354.7903 | 86.69 |
| 47202498 | -8.6 | | 2 | Y | 3 | 0 | 2.8 | 282.3122032 | 32.67 |
| 7970309 | -8.6 | | 5 | Y | 6 | 1 | 2.35 | 397.49062 | 92.43 |
| 26542249 | -8.6 | | 4 | Y | 3 | 1 | 3.42 | 276.33244 | 41.99 |
| 862310 | -8.6 | | 4 | Y | 7 | 1 | 3.9 | 407.44238 | 111.78 |
| 85736039 | -8.6 | | 1 | Y | 7 | 1 | 1.65 | 283.28532 | 85.57 |
| 49727910 | -8.6 | | 5 | Y | 7 | 0 | 2.11 | 418.5529 | 83.89 |
| 24358005 | -8.6 | | 4 | Y | 4 | 1 | 3.65 | 338.4019032 | 71.8 |
| 24372737 | -8.6 | | 3 | Y | 5 | 1 | 3.5 | 348.37518 | 100.44 |
| 24370237 | -8.6 | | 5 | Y | 7 | 1 | 2.29 | 363.36668 | 86.36 |
| 26624876 | -8.6 | | 4 | Y | 4 | 0 | 3.31 | 346.42228 | 42.31 |
| 856913 | -8.6 | | 2 | Y | 6 | 0 | 2.78 | 321.37636 | 54.26 |
| 47202415 | -8.6 | | 6 | Y | 7 | 1 | 3.54 | 346.38582 | 77.75 |
| 24785163 | -8.6 | | 4 | Y | 5 | 0 | 3.2 | 424.5541232 | 40.88 |
| 57259444 | -8.6 | | 4 | Y | 6 | 0 | 2.72 | 431.5235432 | 69.31 |
| 847095 | -8.6 | | 4 | Y | 7 | 1 | 3.66 | 337.37576 | 85.84 |
| 3713902 | -8.6 | | 4 | Y | 6 | 1 | 2.35 | 338.35722 | 92.32 |
| 49821532 | -8.6 | | 6 | Y | 7 | 1 | 3.54 | 389.40396 | 95.23 |
| 26649115 | -8.6 | | 3 | Y | 6 | 1 | 1.83 | 392.49402 | 62.66 |
| 49649727 | -8.6 | | 4 | Y | 6 | 0 | 2.42 | 353.3653432 | 73.45 |
| 124391216 | -8.6 | | 5 | Y | 7 | 2 | 3.8 | 433.3294 | 98.5 |
| 7977293 | -8.6 | | 2 | Y | 5 | 0 | 2.88 | 352.81748 | 63.61 |
| 103162866 | -8.6 | | 4 | Y | 7 | 1 | 2.87 | 337.3078832 | 85.84 |
| 24283180 | -8.6 | | 2 | Y | 7 | 0 | 0.73 | 352.38548 | 129.01 |
| 47195158 | -8.6 | | 6 | Y | 6 | 0 | 3.24 | 449.38456 | 48.3 |
| 17431693 | -8.6 | | 6 | Y | 6 | 0 | 3.85 | 404.3904832 | 66.92 |
| 49828001 | -8.6 | | 4 | Y | 7 | 2 | 3.38 | 423.5312 | 115.46 |
| 57268522 | -8.6 | | 6 | Y | 7 | 1 | 1.74 | 401.47932 | 95.17 |
| 26637797 | -8.6 | | 8 | Y | 7 | 1 | 3.39 | 435.49554 | 110.69 |
| 17516110 | -8.6 | | 8 | Y | 6 | 1 | 3.29 | 399.46014 | 101.68 |
| 26532259 | -8.6 | | 3 | Y | 6 | 2 | 3.14 | 334.80066 | 70.4 |
| 17458297 | -8.6 | | 4 | Y | 6 | 0 | 3.82 | 320.29888 | 84.48 |
| 24346232 | -8.6 | | 1 | Y | 7 | 2 | 0.09 | 350.37122 | 97.77 |
| 26638652 | -8.6 | | 5 | Y | 5 | 1 | 3.49 | 388.4557432 | 86.88 |
| 24353832 | -8.6 | | 6 | Y | 7 | 1 | 3.23 | 378.37802 | 86.75 |
| 24409882 | -8.6 | | 5 | Y | 5 | 1 | 3.81 | 412.5251 | 103.1 |
| 24409121 | -8.6 | | 5 | Y | 7 | 1 | 2.36 | 423.51128 | 146.28 |
| 17461324 | -8.6 | | 6 | Y | 5 | 1 | 2.99 | 341.381 | 102.81 |
| 49641310 | -8.6 | | 2 | Y | 7 | 0 | 0.71 | 367.4631 | 78.54 |
| 103051035 | -8.6 | | 6 | Y | 7 | 1 | 3.64 | 428.48306 | 83.89 |
| 17387980 | -8.6 | | 3 | Y | 7 | 0 | 2.58 | 334.7576 | 83.95 |
| 26541254 | -8.6 | | 4 | Y | 5 | 2 | 3.93 | 381.51766 | 123.16 |
| 843008 | -8.6 | | 2 | Y | 6 | 0 | 0.86 | 394.263 | 60.93 |
| 103073545 | -8.6 | | 2 | Y | 4 | 0 | 3.39 | 366.4286232 | 40.62 |
| 7967412 | -8.6 | | 3 | Y | 7 | 1 | 2.52 | 297.3119 | 73.57 |
| 24400174 | -8.6 | | 4 | Y | 6 | 0 | 2.94 | 366.45674 | 60.13 |
| 26635243 | -8.6 | | 4 | Y | 7 | 1 | 3.93 | 368.40964 | 129.16 |
| 24283859 | -8.6 | | 4 | Y | 6 | 0 | 2.18 | 446.33594 | 97.51 |
| 17462706 | -8.6 | | 6 | Y | 6 | 2 | 2.57 | 352.41024 | 112.85 |
| 57268731 | -8.6 | | 4 | Y | 7 | 0 | 1.69 | 405.51108 | 86.38 |
| 26756361 | -8.6 | | 6 | Y | 7 | 0 | 3.71 | 407.41594 | 74.3 |
| 144097752 | -8.6 | | 6 | Y | 7 | 2 | 3.12 | 423.8458032 | 129.39 |
| 49678183 | -8.6 | | 5 | Y | 4 | 1 | 3.99 | 379.47204 | 71.62 |
| 26613643 | -8.6 | | 3 | Y | 7 | 3 | 1.73 | 345.3547 | 106.66 |
| 24832040 | -8.6 | | 5 | Y | 5 | 1 | 3.59 | 350.41098 | 70.05 |
| 17437978 | -8.6 | | 6 | Y | 7 | 3 | 3.38 | 382.40978 | 99.88 |
| 17444557 | -8.6 | | 2 | Y | 5 | 1 | 3.76 | 336.41084 | 85.23 |
| 7966704 | -8.6 | | 3 | Y | 7 | 0 | 1.68 | 329.35046 | 68.04 |
| 49675505 | -8.6 | | 5 | Y | 7 | 1 | 2.03 | 416.49066 | 106.2 |
| 24779439 | -8.6 | | 2 | Y | 7 | 3 | 3.16 | 365.77318 | 106.66 |
| 864810 | -8.6 | | 4 | Y | 7 | 1 | 2.49 | 421.48888 | 82.33 |
| 24295795 | -8.6 | | 3 | Y | 6 | 1 | 2.3 | 342.41542 | 96.29 |
| 24407248 | -8.6 | | 5 | Y | 6 | 1 | 2.45 | 340.41946 | 67.23 |
| 99356270 | -8.6 | | 4 | Y | 6 | 1 | 2.57 | 330.35836 | 91.93 |
| 49823167 | -8.6 | | 3 | Y | 5 | 1 | 3.2 | 314.35896 | 100.22 |
| 26641054 | -8.6 | | 4 | Y | 6 | 1 | 2.21 | 373.48914 | 55.24 |
| 49737306 | -8.6 | | 4 | Y | 7 | 1 | 1.41 | 386.4200232 | 76.46 |
| 49820259 | -8.6 | | 5 | Y | 7 | 1 | 2.24 | 359.37314 | 99.88 |
| 49827821 | -8.6 | | 3 | Y | 7 | 0 | 1.29 | 374.8662 | 112.13 |
| 49735898 | -8.6 | | 3 | Y | 6 | 1 | 1.81 | 367.42158 | 89.44 |
| 24354982 | -8.6 | | 5 | Y | 6 | 2 | 3.19 | 442.3058 | 80.2 |
| 49827567 | -8.6 | | 6 | Y | 6 | 2 | 2.98 | 380.43696 | 84.5 |
| 103060612 | -8.6 | | 4 | Y | 7 | 1 | 2.7 | 421.3698296 | 80.76 |
| 49723201 | -8.6 | | 5 | Y | 7 | 2 | 1.12 | 391.46458 | 138.91 |
| 56462830 | -8.5 | | 7 | Y | 6 | 1 | 3.85 | 433.54264 | 71 |
| 49722671 | -8.5 | | 3 | Y | 5 | 0 | 2.11 | 330.42134 | 49.85 |
| 49668088 | -8.5 | | 8 | Y | 6 | 1 | 2.24 | 398.4953 | 62.24 |
| 92763275 | -8.5 | | 5 | Y | 7 | 3 | 1.01 | 326.34982 | 99.75 |
| 24828692 | -8.5 | | 4 | Y | 6 | 0 | 2.48 | 406.45432 | 92.79 |
| 24334952 | -8.5 | | 1 | Y | 4 | 2 | 3.48 | 307.38954 | 58.11 |
| 26615356 | -8.5 | | 2 | Y | 6 | 0 | 3.29 | 378.44752 | 87.85 |
| 144097705 | -8.5 | | 4 | Y | 7 | 2 | 1.35 | 377.43962 | 81.33 |
| 24780312 | -8.5 | | 8 | Y | 7 | 0 | 3.26 | 429.51418 | 58.54 |
| 4240933 | -8.5 | | 4 | Y | 6 | 0 | 3.15 | 327.35772 | 91.02 |
| 26613528 | -8.5 | | 5 | Y | 6 | 1 | 1.15 | 345.3681032 | 78.26 |
| 24314674 | -8.5 | | 4 | Y | 7 | 2 | 1.66 | 327.36102 | 134.32 |
| 24784509 | -8.5 | | 6 | Y | 6 | 1 | 3.38 | 390.47814 | 61.46 |
| 17511891 | -8.5 | | 4 | Y | 5 | 0 | 2.65 | 339.79884 | 51.66 |
| 49818741 | -8.5 | | 7 | Y | 7 | 1 | 2.15 | 392.3319096 | 90.14 |
| 26658174 | -8.5 | | 7 | Y | 7 | 1 | 3.21 | 436.90914 | 101.16 |
| 99356938 | -8.5 | | 7 | Y | 7 | 1 | 2.23 | 441.5631 | 78.95 |
| 49732361 | -8.5 | | 6 | Y | 5 | 1 | 3.27 | 396.4546032 | 58.64 |
| 26649014 | -8.5 | | 3 | Y | 5 | 1 | 1.26 | 328.3839432 | 52.23 |
| 124948038 | -8.5 | | 6 | Y | 6 | 1 | 3.62 | 429.55562 | 120.17 |
| 24839050 | -8.5 | | 5 | Y | 7 | 2 | 2.65 | 440.8730232 | 114.99 |
| 49679100 | -8.5 | | 7 | Y | 5 | 3 | 2.27 | 366.3343896 | 78.43 |
| 24782025 | -8.5 | | 6 | Y | 5 | 0 | 2.23 | 391.3869096 | 63.16 |
| 17462948 | -8.5 | | 3 | Y | 6 | 0 | 3.2 | 418.89386 | 98.54 |
| 26640187 | -8.5 | | 6 | Y | 6 | 2 | 1.68 | 370.3742632 | 82.63 |
| 57259785 | -8.5 | | 7 | Y | 7 | 2 | 2.9 | 415.5059 | 103.96 |
| 14721086 | -8.5 | | 8 | Y | 6 | 1 | 2.68 | 357.42676 | 107.61 |
| 24779649 | -8.5 | | 4 | Y | 5 | 0 | 2.44 | 348.42154 | 84.28 |
| 57262028 | -8.5 | | 3 | Y | 7 | 0 | 1.63 | 427.94544 | 86.38 |
| 24323741 | -8.5 | | 3 | Y | 6 | 2 | 3.4 | 406.45432 | 108.66 |
| 24313771 | -8.5 | | 3 | Y | 4 | 2 | 3.33 | 374.22854 | 66.4 |
| 57265069 | -8.5 | | 5 | Y | 7 | 2 | 1.93 | 399.46344 | 119.55 |
| 24369502 | -8.5 | | 5 | Y | 7 | 1 | 3.42 | 380.80282 | 117.44 |
| 26670498 | -8.5 | | 3 | Y | 6 | 0 | 1.6 | 310.3305 | 97.62 |
| 24800127 | -8.5 | | 8 | Y | 7 | 1 | 2.44 | 402.44578 | 90.14 |
| 103050752 | -8.5 | | 6 | Y | 7 | 1 | 2.14 | 391.4845 | 100.89 |
| 3711558 | -8.5 | | 6 | Y | 6 | 0 | 3.09 | 435.58334 | 135.44 |
| 144097755 | -8.5 | | 6 | Y | 6 | 1 | 2.87 | 407.4822232 | 115.99 |
| 57267940 | -8.5 | | 4 | Y | 7 | 2 | 3.06 | 381.42502 | 79.9 |
| 85268305 | -8.5 | | 5 | Y | 7 | 0 | 3.66 | 360.40426 | 92.43 |
| 24800850 | -8.5 | | 8 | Y | 6 | 1 | 3.47 | 429.5275832 | 68.65 |
| 103060516 | -8.5 | | 4 | Y | 3 | 1 | 3.66 | 344.3783664 | 70.23 |
| 26647870 | -8.5 | | 3 | Y | 5 | 0 | 3.52 | 336.3844 | 55.57 |
| 104224799 | -8.5 | | 4 | Y | 6 | 1 | 3.3 | 384.5216 | 117.51 |
| 24373200 | -8.5 | | 4 | Y | 4 | 2 | 2.89 | 304.34254 | 62.22 |
| 847044 | -8.5 | | 5 | Y | 5 | 1 | 3.28 | 342.4784 | 58.12 |
| 24296151 | -8.5 | | 3 | Y | 5 | 0 | 3.3 | 340.39624 | 78.65 |
| 853249 | -8.5 | | 5 | Y | 6 | 2 | 3.76 | 339.38834 | 68.02 |
| 3716629 | -8.5 | | 5 | Y | 5 | 0 | 2.7 | 378.50888 | 94.31 |
| 26614781 | -8.5 | | 4 | Y | 7 | 1 | 2.31 | 354.40298 | 77.63 |
| 24309909 | -8.5 | | 6 | Y | 7 | 0 | 2.74 | 422.88256 | 92.37 |
| 51086975 | -8.5 | | 1 | Y | 5 | 0 | 2.04 | 305.3306 | 55.2 |
| 4265168 | -8.5 | | 6 | Y | 6 | 1 | 2.48 | 394.50828 | 100.31 |
| 26646992 | -8.5 | | 5 | Y | 7 | 1 | 2.5 | 393.43572 | 84.78 |
| 99360829 | -8.5 | | 4 | Y | 4 | 1 | 3.17 | 312.33978 | 95.64 |
| 17513968 | -8.5 | | 3 | Y | 6 | 1 | 3.54 | 297.26224 | 96.26 |
| 24286279 | -8.5 | | 1 | Y | 5 | 0 | 2.36 | 328.38554 | 82 |
| 16953797 | -8.5 | | 7 | Y | 7 | 2 | 3.15 | 447.955 | 87.74 |
| 49723623 | -8.5 | | 6 | Y | 6 | 1 | 3.79 | 419.90668 | 72.7 |
| 26647311 | -8.5 | | 7 | Y | 5 | 0 | 3.38 | 414.5129432 | 54.96 |
| 49676959 | -8.5 | | 5 | Y | 6 | 0 | 3.56 | 363.3122064 | 65.07 |
| 4260534 | -8.5 | | 3 | Y | 6 | 2 | 2.3 | 309.2466096 | 78.33 |
| 110167687 | -8.5 | | 6 | Y | 7 | 2 | 2.59 | 401.49924 | 87.74 |
| 51086373 | -8.5 | | 3 | Y | 6 | 1 | 2.24 | 371.4501232 | 112.52 |
| 24289291 | -8.5 | | 6 | Y | 6 | 2 | 3.12 | 374.43568 | 83.98 |
| 24794708 | -8.5 | | 4 | Y | 5 | 0 | 2.43 | 404.50142 | 57.69 |
| 26652403 | -8.5 | | 0 | Y | 3 | 1 | 3.96 | 249.26404 | 46 |
| 24307072 | -8.5 | | 2 | Y | 6 | 0 | 3.95 | 341.31964 | 80.71 |
| 87334141 | -8.5 | | 0 | Y | 3 | 0 | 3.3 | 247.24816 | 43.1 |
| 26536369 | -8.5 | | 3 | Y | 5 | 0 | 3.25 | 355.81812 | 47.36 |
| 14745985 | -8.5 | | 6 | Y | 6 | 0 | 3.26 | 442.55108 | 105.23 |
| 24832705 | -8.5 | | 3 | Y | 4 | 1 | 2.68 | 281.3059 | 55.4 |
| 4250356 | -8.5 | | 6 | Y | 6 | 1 | 2.7 | 358.3635632 | 69.15 |
| 144089560 | -8.5 | | 3 | Y | 5 | 0 | 3.45 | 372.48116 | 58.23 |
| 24794553 | -8.5 | | 2 | Y | 6 | 1 | 2.39 | 319.31412 | 83.98 |
| 26647756 | -8.5 | | 6 | Y | 6 | 1 | 2.52 | 410.55236 | 58.78 |
| 26618000 | -8.5 | | 3 | Y | 7 | 1 | 3.12 | 411.5205 | 112.12 |
| 24815810 | -8.5 | | 3 | Y | 6 | 1 | 2.6 | 329.3553 | 72.18 |
| 863336 | -8.5 | | 5 | Y | 7 | 1 | 2.9 | 354.40298 | 80.34 |
| 843517 | -8.5 | | 4 | Y | 6 | 1 | 2.76 | 342.41542 | 100.32 |
| 99358245 | -8.5 | | 1 | Y | 3 | 0 | 3.14 | 265.3065 | 29.54 |
| 858213 | -8.5 | | 3 | Y | 4 | 1 | 3.77 | 278.30526 | 55.24 |
| 49818431 | -8.5 | | 3 | Y | 5 | 1 | 3.59 | 327.4207 | 58.37 |
| 24798801 | -8.5 | | 8 | Y | 7 | 0 | 3.22 | 445.8712432 | 77.69 |
| 11536786 | -8.5 | | 1 | Y | 5 | 1 | 1.65 | 287.2889632 | 66.06 |
| 4245564 | -8.5 | | 2 | Y | 6 | 0 | 2.93 | 327.33128 | 57.23 |
| 26532015 | -8.5 | | 2 | Y | 6 | 1 | 1.74 | 315.34702 | 92.59 |
| 47199419 | -8.5 | | 4 | Y | 4 | 1 | 3.95 | 332.3957 | 49.41 |
| 26642147 | -8.5 | | 2 | Y | 7 | 0 | 1.58 | 306.32196 | 75.75 |
| 24386805 | -8.5 | | 3 | Y | 6 | 2 | 2.46 | 309.3193 | 86.53 |
| 49824868 | -8.5 | | 6 | Y | 7 | 1 | 2.68 | 387.4263 | 99.88 |
| 24798475 | -8.5 | | 7 | Y | 7 | 1 | 2.96 | 379.4555 | 85.84 |
| 96021148 | -8.5 | | 5 | Y | 6 | 2 | 3.15 | 377.3172696 | 79.37 |
| 49675053 | -8.5 | | 4 | Y | 6 | 2 | 2.17 | 296.27748 | 95.42 |
| 49673235 | -8.5 | | 4 | Y | 5 | 1 | 2.13 | 324.3737 | 58.64 |
| 14739137 | -8.5 | | 5 | Y | 7 | 1 | 2.29 | 364.42424 | 114.65 |
| 24832318 | -8.5 | | 5 | Y | 7 | 2 | 1.86 | 356.41886 | 90.33 |
| 3715157 | -8.5 | | 5 | Y | 6 | 2 | 2.84 | 343.3522232 | 82.53 |
| 49717584 | -8.5 | | 6 | Y | 6 | 1 | 3.03 | 429.5076632 | 79.79 |
| 26632316 | -8.5 | | 3 | Y | 4 | 0 | 3.02 | 335.39634 | 42.68 |
| 17505891 | -8.5 | | 7 | Y | 7 | 1 | 3.08 | 449.50382 | 90.5 |
| 26620406 | -8.5 | | 1 | Y | 7 | 3 | 2.76 | 330.72584 | 106.18 |
| 51087186 | -8.5 | | 5 | Y | 6 | 1 | 2.78 | 388.48056 | 84.09 |
| 103051331 | -8.5 | | 3 | Y | 6 | 1 | 3.28 | 371.38868 | 82.6 |
| 24373004 | -8.5 | | 4 | Y | 5 | 0 | 3.72 | 350.39106 | 90.4 |
| 7999915 | -8.5 | | 2 | Y | 7 | 1 | 2.31 | 325.36506 | 82.26 |
| 103076311 | -8.5 | | 1 | Y | 4 | 0 | 2.98 | 246.2667 | 43.08 |
| 17513419 | -8.5 | | 6 | Y | 7 | 2 | 2.88 | 400.81238 | 115.39 |
| 24398889 | -8.5 | | 6 | Y | 6 | 1 | 2.62 | 420.90974 | 91.93 |
| 24832382 | -8.5 | | 5 | Y | 6 | 4 | 1.86 | 359.3235896 | 109.67 |
| 26647629 | -8.5 | | 6 | Y | 6 | 0 | 1.48 | 394.5099 | 42.15 |
| 24300451 | -8.5 | | 1 | Y | 4 | 0 | 2.51 | 296.3636 | 42.43 |
| 49818412 | -8.5 | | 4 | Y | 5 | 1 | 1.44 | 364.8664 | 60.85 |
| 24390474 | -8.5 | | 3 | Y | 4 | 1 | 3.62 | 321.36976 | 59.31 |
| 851113 | -8.5 | | 4 | Y | 5 | 1 | 3.67 | 318.37242 | 55.36 |
| 3712987 | -8.5 | | 7 | Y | 6 | 2 | 3.22 | 350.36792 | 80.57 |
| 843457 | -8.5 | | 5 | Y | 7 | 1 | 2.46 | 434.4563896 | 128.88 |
| 124948996 | -8.5 | | 5 | Y | 7 | 1 | 1.18 | 397.51384 | 73.48 |
| 26731123 | -8.5 | | 5 | Y | 6 | 0 | 2.75 | 408.89904 | 67.46 |
| 22414457 | -8.5 | | 3 | Y | 3 | 0 | 3.14 | 322.2570128 | 34.89 |
| 860947 | -8.5 | | 3 | Y | 5 | 1 | 1.28 | 347.3295096 | 66.84 |
| 49819423 | -8.5 | | 6 | Y | 5 | 2 | 3.51 | 335.35328 | 78.46 |
| 4247277 | -8.5 | | 3 | Y | 6 | 1 | 3.47 | 322.36112 | 72.95 |
| 26660504 | -8.5 | | 0 | Y | 3 | 0 | 2.73 | 268.69776 | 37.27 |
| 49817478 | -8.5 | | 7 | Y | 6 | 0 | 2.47 | 387.44944 | 89.13 |
| 24275642 | -8.5 | | 3 | Y | 5 | 1 | 3.73 | 401.3865432 | 70.75 |
| 24271114 | -8.5 | | 3 | Y | 6 | 1 | 3.23 | 365.42562 | 75.44 |
| 49680596 | -8.5 | | 3 | Y | 4 | 3 | 3.75 | 391.30212 | 72.19 |
| 24818310 | -8.5 | | 6 | Y | 7 | 1 | 3.79 | 392.45096 | 88.17 |
| 126700184 | -8.5 | | 5 | Y | 6 | 1 | 3.31 | 387.4958 | 87.75 |
| 49681604 | -8.5 | | 5 | Y | 4 | 1 | 0.84 | 337.41222 | 53.8 |
| 26736132 | -8.5 | | 5 | Y | 5 | 1 | 2.57 | 335.39964 | 60.73 |
| 124754159 | -8.5 | | 6 | Y | 7 | 1 | 3.1 | 396.43636 | 77.1 |
| 7970413 | -8.5 | | 4 | Y | 7 | 0 | 3.56 | 431.53006 | 65.06 |
| 17461139 | -8.5 | | 7 | Y | 7 | 1 | 3.19 | 403.81302 | 93.14 |
| 99455965 | -8.5 | | 4 | Y | 5 | 1 | 1.61 | 327.3528232 | 62.3 |
| 17506073 | -8.5 | | 2 | Y | 3 | 0 | 3.94 | 346.1505632 | 43.1 |
| 49733506 | -8.5 | | 6 | Y | 5 | 2 | 3.36 | 364.3696832 | 71.34 |
| 26530465 | -8.5 | | 4 | Y | 4 | 0 | 3.98 | 344.2873496 | 51.96 |
| 17510879 | -8.5 | | 5 | Y | 5 | 1 | 3.44 | 416.62314 | 70.91 |
| 124948103 | -8.5 | | 6 | Y | 7 | 1 | 2.13 | 411.88468 | 81.93 |
| 99358209 | -8.5 | | 3 | Y | 5 | 0 | 3.47 | 326.34322 | 53.99 |
| 4248025 | -8.5 | | 5 | Y | 5 | 0 | 3.68 | 388.2672192 | 56.74 |
| 99495293 | -8.5 | | 4 | Y | 6 | 1 | 2.16 | 401.4545032 | 81.86 |
| 24811051 | -8.5 | | 4 | Y | 6 | 1 | 3.19 | 321.33 | 87.81 |
| 103076751 | -8.5 | | 4 | Y | 6 | 1 | 3.61 | 344.36664 | 63.91 |
| 103162762 | -8.5 | | 4 | Y | 7 | 0 | 2.93 | 297.26554 | 93.85 |
| 26728751 | -8.5 | | 4 | Y | 6 | 1 | 3.03 | 351.2799896 | 81.16 |
| 103051193 | -8.5 | | 5 | Y | 6 | 2 | 3.93 | 359.39628 | 123.77 |
| 7965915 | -8.5 | | 4 | Y | 5 | 0 | 3.69 | 380.48002 | 57.69 |
| 104222937 | -8.5 | | 5 | Y | 6 | 2 | 2.54 | 339.38834 | 80.41 |
| 843244 | -8.5 | | 6 | Y | 7 | 1 | 3.01 | 386.42162 | 107.03 |
| 85270033 | -8.5 | | 5 | Y | 5 | 1 | 3.18 | 359.3714632 | 97.77 |
| 24800357 | -8.5 | | 5 | Y | 5 | 0 | 3.01 | 375.4635 | 55.2 |
| 7997942 | -8.5 | | 2 | Y | 6 | 2 | 3.28 | 420.3618496 | 112.68 |
| 24378128 | -8.5 | | 4 | Y | 6 | 0 | 2.25 | 404.89372 | 122.8 |
| 56373579 | -8.5 | | 5 | Y | 6 | 3 | 3.23 | 327.35772 | 124.4 |
| 14735155 | -8.5 | | 2 | Y | 4 | 1 | 3.13 | 300.37544 | 66.91 |
| 49672862 | -8.5 | | 4 | Y | 6 | 0 | 3.2 | 312.31994 | 75.36 |
| 124349858 | -8.5 | | 2 | Y | 3 | 0 | 2.92 | 297.3499832 | 56.01 |
| 57256637 | -8.5 | | 6 | Y | 7 | 2 | 3.08 | 420.2461 | 96.86 |
| 7972724 | -8.5 | | 5 | Y | 7 | 1 | 2.59 | 417.52178 | 102.12 |
| 865387 | -8.5 | | 1 | Y | 4 | 0 | 3.72 | 339.3602232 | 38.77 |
| 26646973 | -8.5 | | 4 | Y | 7 | 2 | 2.08 | 347.37058 | 96.7 |
| 17468736 | -8.5 | | 4 | Y | 4 | 0 | 3.45 | 407.2767032 | 32.78 |
| 24781531 | -8.5 | | 7 | Y | 7 | 1 | 2.87 | 385.45678 | 84.52 |
| 57268207 | -8.5 | | 4 | Y | 6 | 1 | 2.89 | 383.46404 | 87.22 |
| 4252361 | -8.5 | | 5 | Y | 7 | 3 | 3.94 | 381.48968 | 120.27 |
| 104223072 | -8.5 | | 9 | Y | 7 | 1 | 3.23 | 442.52794 | 118.08 |
| 4248523 | -8.5 | | 7 | Y | 6 | 1 | 2.82 | 382.4992 | 68.43 |
| 85145974 | -8.5 | | 2 | Y | 6 | 1 | 2.46 | 352.43016 | 69.3 |
| 846686 | -8.5 | | 2 | Y | 4 | 2 | 3.56 | 275.30462 | 75.84 |
| 17461476 | -8.5 | | 3 | Y | 6 | 1 | 3.71 | 356.75982 | 73.59 |
| 121285540 | -8.5 | | 2 | Y | 4 | 0 | 3.85 | 361.27944 | 33.95 |
| 3711676 | -8.5 | | 3 | Y | 6 | 1 | 2.13 | 313.35106 | 79.01 |
| 49676879 | -8.5 | | 6 | Y | 6 | 2 | 2.67 | 380.48332 | 65.88 |
| 24332456 | -8.5 | | 2 | Y | 3 | 0 | 3.62 | 277.3172 | 39.17 |
| 24793735 | -8.5 | | 3 | Y | 6 | 0 | 3.63 | 317.33646 | 89.19 |
| 17503868 | -8.5 | | 7 | Y | 6 | 1 | 4 | 435.5386 | 83.81 |
| 49828957 | -8.5 | | 5 | Y | 7 | 1 | 3.04 | 354.38306 | 126 |
| 26658417 | -8.5 | | 3 | Y | 6 | 1 | 3.28 | 323.39224 | 66.81 |
| 26531190 | -8.5 | | 3 | Y | 5 | 0 | 3.06 | 321.2540096 | 53.51 |
| 17517036 | -8.5 | | 8 | Y | 7 | 1 | 3.65 | 436.54496 | 132.97 |
| 859261 | -8.5 | | 5 | Y | 7 | 1 | 2.09 | 351.38242 | 130 |
| 56321898 | -8.5 | | 3 | Y | 6 | 1 | 2.13 | 391.87182 | 97.96 |
| 26541748 | -8.5 | | 3 | Y | 7 | 1 | 3.63 | 423.48484 | 110.16 |
| 24808230 | -8.5 | | 8 | Y | 6 | 1 | 3.16 | 431.50684 | 103.68 |
| 134216001 | -8.5 | | 3 | Y | 4 | 1 | 2.41 | 317.38436 | 51.62 |
| 24788276 | -8.5 | | 4 | Y | 6 | 2 | 2.29 | 368.471 | 114.3 |
| 17386253 | -8.5 | | 6 | Y | 7 | 2 | 2.84 | 432.55956 | 103.26 |
| 49736904 | -8.5 | | 6 | Y | 7 | 0 | 2.8 | 393.43572 | 77.69 |
| 49669443 | -8.5 | | 6 | Y | 6 | 0 | 3.23 | 377.45462 | 73.45 |
| 14737914 | -8.5 | | 7 | Y | 6 | 1 | 2.92 | 400.47296 | 67.91 |
| 14726366 | -8.5 | | 5 | Y | 5 | 0 | 3.89 | 363.40644 | 59.75 |
| 24365857 | -8.5 | | 5 | Y | 7 | 2 | 1.97 | 413.47172 | 92.25 |
| 22412053 | -8.5 | | 5 | Y | 6 | 1 | 2.96 | 363.4561 | 64.74 |
| 49816068 | -8.5 | | 2 | Y | 5 | 0 | 2.03 | 287.29056 | 79.9 |
| 26541378 | -8.5 | | 2 | Y | 6 | 1 | 1.43 | 300.3556 | 61.61 |
| 124894077 | -8.4 | | 6 | Y | 4 | 2 | 3.6 | 322.40088 | 58.7 |
| 49647867 | -8.4 | | 8 | Y | 7 | 3 | 2.87 | 381.42502 | 92.45 |
| 24838499 | -8.4 | | 6 | Y | 6 | 1 | 3.8 | 409.8655 | 63.69 |
| 26530761 | -8.4 | | 0 | Y | 4 | 1 | 2.79 | 300.39536 | 33.73 |
| 103060502 | -8.4 | | 4 | Y | 4 | 1 | 3.97 | 355.3827632 | 83.73 |
| 26536911 | -8.4 | | 4 | Y | 5 | 0 | 3.26 | 367.2464592 | 51.39 |
| 49720596 | -8.4 | | 5 | Y | 6 | 0 | 2.49 | 374.4739 | 59.08 |
| 49828510 | -8.4 | | 4 | Y | 7 | 2 | 2.09 | 368.42956 | 108.62 |
| 861924 | -8.4 | | 5 | Y | 5 | 0 | 3.09 | 349.42622 | 47.48 |
| 49725185 | -8.4 | | 6 | Y | 6 | 0 | 3.39 | 449.56518 | 69.31 |
| 24351860 | -8.4 | | 6 | Y | 7 | 1 | 3.4 | 446.49504 | 77.1 |
| 46500322 | -8.4 | | 5 | Y | 6 | 0 | 3.29 | 349.38316 | 69.74 |
| 17437583 | -8.4 | | 2 | Y | 4 | 0 | 2.34 | 290.31596 | 49.74 |
| 848308 | -8.4 | | 7 | Y | 7 | 1 | 2.11 | 389.45032 | 80.12 |
| 24377696 | -8.4 | | 5 | Y | 5 | 1 | 2.43 | 390.51958 | 100.16 |
| 56317325 | -8.4 | | 8 | Y | 7 | 1 | 3.48 | 444.54382 | 101.16 |
| 51085661 | -8.4 | | 5 | Y | 4 | 1 | 3.68 | 332.3957 | 45.48 |
| 49724057 | -8.4 | | 3 | Y | 6 | 1 | 1.57 | 324.33064 | 67.87 |
| 24387509 | -8.4 | | 4 | Y | 7 | 1 | 2.85 | 368.42956 | 77.63 |
| 24287092 | -8.4 | | 2 | Y | 7 | 2 | 2.66 | 309.27624 | 114.41 |
| 49736842 | -8.4 | | 6 | Y | 7 | 1 | 2.42 | 354.40298 | 82.18 |
| 24288953 | -8.4 | | 3 | Y | 7 | 1 | 3.58 | 380.42034 | 118.04 |
| 4245741 | -8.4 | | 6 | Y | 5 | 1 | 3.21 | 365.44876 | 89.29 |
| 50085633 | -8.4 | | 4 | Y | 6 | 2 | 2.07 | 389.8079832 | 78.51 |
| 17507000 | -8.4 | | 6 | Y | 7 | 2 | 3.13 | 360.38434 | 109.59 |
| 26538154 | -8.4 | | 5 | Y | 7 | 1 | 2.34 | 426.8944 | 130.09 |
| 11537496 | -8.4 | | 3 | Y | 5 | 1 | 2.67 | 298.29006 | 68.9 |
| 49818293 | -8.4 | | 7 | Y | 6 | 1 | 3.24 | 429.57554 | 78.1 |
| 24373321 | -8.4 | | 4 | Y | 4 | 1 | 3.2 | 307.34318 | 59.31 |
| 49737622 | -8.4 | | 6 | Y | 5 | 1 | 3.98 | 405.4646632 | 55.66 |
| 92764337 | -8.4 | | 1 | Y | 3 | 1 | 2.34 | 255.2438032 | 46.17 |
| 24305780 | -8.4 | | 7 | Y | 6 | 0 | 3.39 | 416.53372 | 75.3 |
| 49724673 | -8.4 | | 5 | Y | 6 | 1 | 3.2 | 368.42626 | 67.87 |
| 24289918 | -8.4 | | 3 | Y | 6 | 1 | 3.06 | 402.8514 | 98.78 |
| 49828580 | -8.4 | | 3 | Y | 7 | 0 | 1.82 | 388.4375 | 84.53 |
| 124948860 | -8.4 | | 6 | Y | 6 | 1 | 2.38 | 378.44752 | 102.3 |
| 103075075 | -8.4 | | 3 | Y | 4 | 1 | 3.13 | 324.41676 | 49.41 |
| 26614481 | -8.4 | | 4 | Y | 6 | 0 | 2.58 | 340.41946 | 52.97 |
| 14741196 | -8.4 | | 2 | Y | 5 | 0 | 3.29 | 351.4421 | 48.93 |
| 57257198 | -8.4 | | 7 | Y | 7 | 1 | 2.58 | 402.46408 | 101.16 |
| 49716415 | -8.4 | | 4 | Y | 6 | 1 | 2.55 | 363.45448 | 117.16 |
| 17449709 | -8.4 | | 3 | Y | 3 | 0 | 3.23 | 281.34896 | 29.54 |
| 3716324 | -8.4 | | 4 | Y | 4 | 0 | 3.69 | 319.38032 | 77.11 |
| 4258155 | -8.4 | | 2 | Y | 5 | 0 | 2.21 | 281.26284 | 55.84 |
| 26532479 | -8.4 | | 4 | Y | 6 | 0 | 3.81 | 337.76308 | 65.72 |
| 24279475 | -8.4 | | 4 | Y | 7 | 0 | 3.21 | 408.4735 | 102.74 |
| 125305683 | -8.4 | | 6 | Y | 6 | 1 | 3.47 | 428.54442 | 93.17 |
| 49720283 | -8.4 | | 7 | Y | 7 | 1 | 3.98 | 403.85938 | 90.39 |
| 26532137 | -8.4 | | 4 | Y | 4 | 0 | 3.21 | 344.3520096 | 69.55 |
| 24284178 | -8.4 | | 3 | Y | 6 | 0 | 3.62 | 390.43178 | 83.2 |
| 56322139 | -8.4 | | 5 | Y | 6 | 0 | 3.66 | 345.38962 | 68.98 |
| 4263689 | -8.4 | | 3 | Y | 7 | 3 | 1.56 | 369.46068 | 96.52 |
| 17514202 | -8.4 | | 4 | Y | 5 | 0 | 3.94 | 343.33222 | 65.49 |
| 11535298 | -8.4 | | 3 | Y | 5 | 1 | 2.09 | 271.31438 | 68.02 |
| 17468621 | -8.4 | | 5 | Y | 5 | 1 | 3.72 | 354.42282 | 99.15 |
| 26630255 | -8.4 | | 2 | Y | 5 | 1 | 3.7 | 362.3489896 | 60.14 |
| 49729235 | -8.4 | | 5 | Y | 6 | 0 | 1.91 | 352.43016 | 66.4 |
| 17406612 | -8.4 | | 3 | Y | 7 | 2 | 3.74 | 384.22214 | 95.4 |
| 4248840 | -8.4 | | 5 | Y | 6 | 0 | 3.27 | 357.42676 | 110.28 |
| 57259075 | -8.4 | | 5 | Y | 7 | 3 | 0.42 | 367.4631 | 117.95 |
| 7977086 | -8.4 | | 1 | Y | 3 | 0 | 3.31 | 275.34768 | 30.71 |
| 17401836 | -8.4 | | 3 | Y | 5 | 2 | 3.44 | 389.47016 | 109.28 |
| 14730291 | -8.4 | | 6 | Y | 5 | 2 | 3.97 | 366.3608296 | 91.26 |
| 26650570 | -8.4 | | 8 | Y | 5 | 0 | 3.59 | 407.89268 | 51.27 |
| 14729452 | -8.4 | | 3 | Y | 5 | 0 | 3.33 | 342.39382 | 62.04 |
| 24391090 | -8.4 | | 2 | Y | 5 | 0 | 3.61 | 367.4382 | 63.66 |
| 7968484 | -8.4 | | 5 | Y | 5 | 0 | 2.93 | 395.4698432 | 43.86 |
| 26641661 | -8.4 | | 4 | Y | 7 | 1 | 3.23 | 394.49328 | 112.14 |
| 3714511 | -8.4 | | 5 | Y | 5 | 1 | 2.51 | 358.38992 | 70.5 |
| 26616862 | -8.4 | | 7 | Y | 5 | 0 | 3.83 | 414.5593032 | 41.37 |
| 24785157 | -8.4 | | 4 | Y | 4 | 0 | 3.77 | 370.44368 | 50.27 |
| 47204751 | -8.4 | | 4 | Y | 5 | 2 | 3.32 | 328.3640232 | 95.15 |
| 26644419 | -8.4 | | 6 | Y | 5 | 1 | 3.56 | 363.43288 | 100.31 |
| 17410725 | -8.4 | | 5 | Y | 4 | 1 | 3.33 | 317.36118 | 53.54 |
| 103159365 | -8.4 | | 3 | Y | 7 | 1 | -0.5 | 337.37576 | 78 |
| 49719909 | -8.4 | | 5 | Y | 7 | 1 | 3.26 | 361.41546 | 110.18 |
| 24399633 | -8.4 | | 3 | Y | 6 | 1 | 2.97 | 309.36566 | 64.86 |
| 22409082 | -8.4 | | 6 | Y | 7 | 1 | 3.01 | 395.81416 | 107.15 |
| 26536138 | -8.4 | | 4 | Y | 5 | 2 | 3.5 | 367.2464592 | 67.16 |
| 4256806 | -8.4 | | 3 | Y | 6 | 0 | 3.97 | 404.3856696 | 62.47 |
| 3713704 | -8.4 | | 5 | Y | 6 | 0 | 3.98 | 383.85466 | 94.93 |
| 24818438 | -8.4 | | 4 | Y | 4 | 0 | 3.25 | 352.81418 | 42.31 |
| 24784841 | -8.4 | | 6 | Y | 5 | 0 | 3.49 | 353.3901032 | 51.39 |
| 859224 | -8.4 | | 2 | Y | 5 | 1 | 3.17 | 318.32606 | 71.09 |
| 22400545 | -8.4 | | 3 | Y | 5 | 1 | 3.36 | 333.4285 | 100.08 |
| 7968993 | -8.4 | | 5 | Y | 6 | 1 | 2.83 | 367.42158 | 105.37 |
| 26536009 | -8.4 | | 3 | Y | 5 | 0 | 3.36 | 306.2938432 | 52.31 |
| 7996935 | -8.4 | | 3 | Y | 5 | 0 | 3.05 | 344.2625896 | 91.04 |
| 17409218 | -8.4 | | 4 | Y | 7 | 2 | 2.03 | 339.34528 | 91.65 |
| 14725514 | -8.4 | | 3 | Y | 5 | 1 | 1.98 | 293.3199 | 63.99 |
| 864329 | -8.4 | | 4 | Y | 5 | 1 | 3.02 | 334.41488 | 52.23 |
| 17442540 | -8.4 | | 6 | Y | 7 | 2 | 2.61 | 437.51142 | 120.82 |
| 26619935 | -8.4 | | 2 | Y | 5 | 2 | 1.88 | 266.2515 | 85.88 |
| 85736243 | -8.4 | | 6 | Y | 7 | 2 | 2.86 | 416.3732728 | 95.76 |
| 24354071 | -8.4 | | 6 | Y | 5 | 0 | 2.92 | 384.3728096 | 88.36 |
| 104224407 | -8.4 | | 3 | Y | 7 | 3 | -0.49 | 377.39326 | 110.17 |
| 49647338 | -8.4 | | 4 | Y | 4 | 1 | 3.51 | 329.3654032 | 44.3 |
| 3713953 | -8.4 | | 6 | Y | 5 | 0 | 3.87 | 394.3875496 | 42.01 |
| 57265992 | -8.4 | | 4 | Y | 6 | 1 | 2.05 | 359.44264 | 87.75 |
| 26615064 | -8.4 | | 5 | Y | 7 | 2 | 1.14 | 370.44544 | 81.69 |
| 121283268 | -8.4 | | 2 | Y | 3 | 2 | 3.79 | 343.41834 | 49.33 |
| 24390253 | -8.4 | | 2 | Y | 6 | 1 | 3.77 | 416.2574 | 69.16 |
| 7965303 | -8.4 | | 4 | Y | 7 | 0 | 2.25 | 361.41546 | 102.99 |
| 14729668 | -8.4 | | 5 | Y | 5 | 1 | 3.45 | 356.4387 | 84.17 |
| 24814875 | -8.4 | | 6 | Y | 6 | 3 | 3.7 | 386.46468 | 126.57 |
| 144090268 | -8.4 | | 4 | Y | 6 | 1 | 3.28 | 424.448 | 92.09 |
| 22407939 | -8.4 | | 5 | Y | 5 | 0 | 3.71 | 369.4126432 | 82.31 |
| 24303752 | -8.4 | | 4 | Y | 5 | 1 | 3.26 | 331.36788 | 59.39 |
| 49825362 | -8.4 | | 4 | Y | 6 | 1 | 3.5 | 378.46744 | 72.95 |
| 124949646 | -8.4 | | 6 | Y | 7 | 1 | 1.84 | 399.5098 | 103.93 |
| 24338644 | -8.4 | | 1 | Y | 6 | 4 | 3.58 | 401.86664 | 139.92 |
| 49649250 | -8.4 | | 3 | Y | 4 | 1 | 3.94 | 330.80866 | 41.57 |
| 26615089 | -8.4 | | 2 | Y | 7 | 3 | 1.76 | 343.40348 | 134.9 |
| 51085221 | -8.4 | | 6 | Y | 7 | 1 | 2.23 | 370.39908 | 77.1 |
| 24812019 | -8.4 | | 1 | Y | 4 | 2 | 3.47 | 299.32602 | 71.93 |
| 26530228 | -8.4 | | 7 | Y | 6 | 1 | 2.64 | 402.50714 | 89.13 |
| 24323727 | -8.4 | | 6 | Y | 6 | 0 | 2.92 | 429.55562 | 103.73 |
| 24826333 | -8.4 | | 4 | Y | 7 | 0 | 3.82 | 439.57366 | 93.95 |
| 7967760 | -8.4 | | 5 | Y | 6 | 0 | 3.22 | 404.52302 | 88.86 |
| 49730607 | -8.4 | | 2 | Y | 4 | 1 | 3.4 | 327.44384 | 72.22 |
| 26649650 | -8.4 | | 6 | Y | 6 | 1 | 2.72 | 383.82822 | 77.13 |
| 49674295 | -8.4 | | 6 | Y | 7 | 2 | 2.68 | 430.3798896 | 92.91 |
| 49676185 | -8.4 | | 5 | Y | 7 | 1 | 2 | 403.4952 | 87.33 |
| 26529406 | -8.4 | | 4 | Y | 5 | 0 | 2.82 | 354.42282 | 78.78 |
| 24783424 | -8.4 | | 4 | Y | 5 | 0 | 2.95 | 369.3614696 | 91.4 |
| 47197452 | -8.4 | | 5 | Y | 7 | 2 | 1.75 | 402.46408 | 114.99 |
| 26615907 | -8.4 | | 4 | Y | 7 | 0 | 2.19 | 364.3978 | 81.35 |
| 124949645 | -8.4 | | 5 | Y | 5 | 0 | 3.63 | 363.4528 | 40.72 |
| 103913571 | -8.4 | | 3 | Y | 6 | 0 | 3.82 | 334.37182 | 60.25 |
| 49665859 | -8.4 | | 3 | Y | 4 | 0 | 2.72 | 266.29456 | 44.24 |
| 26637937 | -8.4 | | 5 | Y | 7 | 1 | 2.17 | 335.3383632 | 81.41 |
| 49726561 | -8.4 | | 3 | Y | 6 | 0 | 1.83 | 371.88218 | 69.31 |
| 24839646 | -8.4 | | 5 | Y | 7 | 1 | 1.52 | 367.42488 | 111.6 |
| 47197496 | -8.4 | | 7 | Y | 5 | 2 | 3.21 | 407.4805432 | 65.2 |
| 17442795 | -8.4 | | 2 | Y | 3 | 0 | 3.76 | 285.2449664 | 38.66 |
| 845762 | -8.4 | | 2 | Y | 5 | 0 | 1.83 | 336.81324 | 49.85 |
| 49679645 | -8.4 | | 7 | Y | 4 | 2 | 1.56 | 333.38046 | 65.97 |
| 24799303 | -8.4 | | 7 | Y | 5 | 1 | 3.52 | 386.4597832 | 52 |
| 89850904 | -8.4 | | 5 | Y | 7 | 1 | 2.77 | 434.51078 | 118.68 |
| 4248607 | -8.4 | | 2 | Y | 5 | 0 | 2.87 | 294.30466 | 59.48 |
| 124349799 | -8.4 | | 6 | Y | 7 | 1 | 2.16 | 352.43346 | 67.58 |
| 26534581 | -8.4 | | 5 | Y | 7 | 3 | 1.93 | 410.50768 | 132.32 |
| 24797731 | -8.4 | | 5 | Y | 7 | 0 | 2.3 | 352.3871 | 75.61 |
| 24339756 | -8.4 | | 5 | Y | 6 | 1 | 3.19 | 371.45334 | 101.46 |
| 57257918 | -8.4 | | 7 | Y | 7 | 1 | 3.3 | 406.47754 | 84.3 |
| 24294726 | -8.4 | | 4 | Y | 6 | 1 | 2.78 | 312.27358 | 88.8 |
| 26646846 | -8.4 | | 5 | Y | 7 | 1 | 1.47 | 313.35436 | 81.4 |
| 861443 | -8.4 | | 7 | Y | 7 | 1 | 3.45 | 439.91792 | 111.14 |
| 26540357 | -8.4 | | 3 | Y | 5 | 2 | 3.69 | 344.428 | 87.22 |
| 99495546 | -8.4 | | 4 | Y | 5 | 1 | 3.4 | 356.37404 | 72.2 |
| 3713996 | -8.4 | | 6 | Y | 6 | 2 | 3.15 | 390.47814 | 79.29 |
| 24813284 | -8.4 | | 6 | Y | 6 | 1 | 3.76 | 404.45836 | 69.56 |
| 49737270 | -8.4 | | 7 | Y | 6 | 0 | 2.53 | 383.48396 | 64.43 |
| 49820975 | -8.4 | | 3 | Y | 6 | 1 | 2.27 | 380.84588 | 89.13 |
| 26614929 | -8.4 | | 3 | Y | 5 | 0 | 3.42 | 348.3951 | 51.66 |
| 26617784 | -8.4 | | 5 | Y | 6 | 1 | 3.02 | 350.41428 | 72.95 |
| 49645683 | -8.4 | | 4 | Y | 6 | 1 | 2.96 | 296.27748 | 88.28 |
| 124948748 | -8.4 | | 6 | Y | 7 | 1 | 2.6 | 416.4988 | 113.83 |
| 22415042 | -8.4 | | 6 | Y | 5 | 2 | 3.37 | 354.44274 | 67.43 |
| 29215635 | -8.4 | | 6 | Y | 5 | 1 | 3.56 | 348.3951 | 60.45 |
| 26646677 | -8.4 | | 5 | Y | 5 | 2 | 3.2 | 377.47938 | 62.58 |
| 24376486 | -8.4 | | 3 | Y | 4 | 1 | 3.97 | 351.44532 | 112.18 |
| 49667986 | -8.4 | | 5 | Y | 6 | 1 | 1.68 | 324.33064 | 75.71 |
| 24347736 | -8.4 | | 4 | Y | 6 | 0 | 3.64 | 429.30872 | 116.88 |
| 49647384 | -8.4 | | 7 | Y | 7 | 2 | 3.43 | 351.40234 | 81.07 |
| 24360686 | -8.4 | | 3 | Y | 6 | 1 | 3.43 | 317.29824 | 87.28 |
| 24781633 | -8.4 | | 3 | Y | 6 | 0 | 3.74 | 401.48416 | 90.46 |
| 104233377 | -8.4 | | 7 | Y | 6 | 0 | 2.86 | 371.43504 | 65.72 |
| 49675193 | -8.4 | | 2 | Y | 2 | 0 | 3.86 | 277.2923832 | 22 |
| 24839337 | -8.4 | | 6 | Y | 7 | 1 | 2.42 | 417.52178 | 90.9 |
| 3714029 | -8.4 | | 3 | Y | 7 | 0 | 3.46 | 310.3338 | 103.27 |
| 24808068 | -8.4 | | 4 | Y | 5 | 0 | 2.91 | 322.35782 | 61.19 |
| 24805906 | -8.4 | | 7 | Y | 7 | 4 | 3.14 | 437.53786 | 123.55 |
| 863400 | -8.4 | | 2 | Y | 4 | 3 | 2.56 | 318.198896 | 64.35 |
| 4261850 | -8.4 | | 4 | Y | 5 | 2 | 1.88 | 330.40142 | 83.65 |
| 848265 | -8.4 | | 4 | Y | 7 | 0 | 3.02 | 416.5171 | 133.08 |
| 26648104 | -8.4 | | 4 | Y | 4 | 2 | 2.71 | 352.4020432 | 58.2 |
| 24280482 | -8.4 | | 5 | Y | 5 | 1 | 3.85 | 356.84924 | 59.29 |
| 24276508 | -8.4 | | 5 | Y | 6 | 0 | 3.03 | 393.43242 | 65.07 |
| 26613716 | -8.4 | | 2 | Y | 5 | 0 | 2.38 | 319.35718 | 55.32 |
| 24799119 | -8.4 | | 4 | Y | 5 | 2 | 3.34 | 412.5235032 | 93.59 |
| 49645549 | -8.4 | | 7 | Y | 7 | 3 | 3.31 | 368.3568696 | 86.2 |
| 49672887 | -8.4 | | 6 | Y | 6 | 1 | 3.77 | 413.46842 | 73.29 |
| 14735319 | -8.4 | | 5 | Y | 6 | 0 | 2.82 | 347.36728 | 66.12 |
| 26638405 | -8.4 | | 5 | Y | 6 | 0 | 3.35 | 379.47864 | 91.63 |
| 24828900 | -8.4 | | 4 | Y | 7 | 0 | 3.24 | 326.30346 | 98.15 |
| 24311131 | -8.4 | | 5 | Y | 6 | 1 | 2.27 | 312.2984232 | 80.91 |
| 49673001 | -8.4 | | 5 | Y | 4 | 2 | 2.59 | 336.42746 | 58.2 |
| 49822738 | -8.4 | | 6 | Y | 6 | 2 | 3.01 | 393.43242 | 88.77 |
| 4251491 | -8.4 | | 4 | Y | 6 | 1 | 2.63 | 378.42108 | 75.19 |
| 85148852 | -8.4 | | 2 | Y | 4 | 0 | 3.17 | 327.76166 | 48.42 |
| 24795684 | -8.4 | | 4 | Y | 7 | 1 | 2.54 | 353.37186 | 95.23 |
| 14735000 | -8.4 | | 3 | Y | 5 | 0 | 3.97 | 352.3805 | 57.9 |
| 24797873 | -8.4 | | 6 | Y | 6 | 0 | 3.97 | 405.48948 | 49.95 |
| 56315436 | -8.4 | | 8 | Y | 6 | 1 | 2.32 | 429.4597064 | 69.72 |
| 17506104 | -8.4 | | 6 | Y | 7 | 2 | 3.64 | 353.37186 | 104.02 |
| 26642293 | -8.4 | | 4 | Y | 5 | 0 | 2.83 | 344.44792 | 43.21 |
| 4251851 | -8.4 | | 4 | Y | 4 | 0 | 2.55 | 362.46474 | 41.82 |
| 49721767 | -8.4 | | 1 | Y | 6 | 0 | 1.78 | 359.44264 | 80.65 |
| 24400796 | -8.4 | | 6 | Y | 5 | 1 | 3.73 | 382.47598 | 77.79 |
| 26647291 | -8.4 | | 5 | Y | 5 | 1 | 2.85 | 363.4528 | 51.72 |
| 24800161 | -8.4 | | 9 | Y | 7 | 1 | 3.05 | 415.4876 | 76.34 |
| 49737352 | -8.4 | | 5 | Y | 6 | 0 | 3.6 | 416.94426 | 50.6 |
| 26537589 | -8.4 | | 6 | Y | 5 | 0 | 3.35 | 383.3880496 | 80.62 |
| 847956 | -8.4 | | 5 | Y | 5 | 1 | 2.5 | 321.37306 | 63.99 |
| 24390582 | -8.4 | | 6 | Y | 7 | 1 | 2.27 | 345.373 | 102.7 |
| 22403330 | -8.4 | | 8 | Y | 7 | 1 | 2.2 | 369.41432 | 90.29 |
| 121285886 | -8.3 | | 4 | Y | 5 | 1 | 3.6 | 347.41034 | 52.65 |
| 7967108 | -8.3 | | 4 | Y | 6 | 0 | 1.17 | 335.37488 | 85.2 |
| 24428908 | -8.3 | | 4 | Y | 5 | 1 | 2.27 | 383.46074 | 92.87 |
| 24300535 | -8.3 | | 4 | Y | 5 | 1 | 3.23 | 390.4716232 | 74.86 |
| 24391020 | -8.3 | | 2 | Y | 5 | 1 | 3.45 | 265.3131 | 55.11 |
| 49670769 | -8.3 | | 7 | Y | 6 | 1 | 2.25 | 366.41038 | 69.56 |
| 26647798 | -8.3 | | 5 | Y | 5 | 1 | 2.62 | 388.4557432 | 74.86 |
| 24399952 | -8.3 | | 5 | Y | 7 | 1 | 3.31 | 418.5346 | 75.94 |
| 26646894 | -8.3 | | 9 | Y | 6 | 0 | 3.03 | 424.53258 | 52.44 |
| 24787241 | -8.3 | | 4 | Y | 4 | 1 | 3.66 | 311.35502 | 80.7 |
| 99495446 | -8.3 | | 5 | Y | 7 | 1 | 2.46 | 441.4241296 | 108.39 |
| 26730014 | -8.3 | | 7 | Y | 5 | 1 | 3.88 | 417.54324 | 47.7 |
| 26651165 | -8.3 | | 3 | Y | 4 | 2 | 3.68 | 408.31822 | 54.13 |
| 125001277 | -8.3 | | 3 | Y | 4 | 0 | 3.85 | 323.41208 | 74.33 |
| 24364207 | -8.3 | | 4 | Y | 7 | 1 | 1.92 | 326.30346 | 103.43 |
| 26514224 | -8.3 | | 5 | Y | 6 | 1 | 2.66 | 344.38494 | 89.47 |
| 4262600 | -8.3 | | 4 | Y | 6 | 1 | 2.14 | 354.42612 | 105.12 |
| 24816619 | -8.3 | | 5 | Y | 5 | 1 | 3.12 | 325.35846 | 64.63 |
| 26662555 | -8.3 | | 4 | Y | 7 | 0 | 2.03 | 328.31934 | 88.5 |
| 24311601 | -8.3 | | 6 | Y | 7 | 2 | 2.38 | 378.37802 | 89.8 |
| 103076350 | -8.3 | | 7 | Y | 6 | 1 | 2.82 | 391.4629 | 67.6 |
| 56320073 | -8.3 | | 5 | Y | 6 | 2 | 3.12 | 313.3047 | 96.19 |
| 24409013 | -8.3 | | 6 | Y | 6 | 1 | 3.28 | 392.4741 | 98.1 |
| 99454785 | -8.3 | | 1 | Y | 4 | 0 | 3 | 254.28386 | 41.9 |
| 14734501 | -8.3 | | 8 | Y | 7 | 2 | 3.54 | 448.55728 | 88.4 |
| 49818158 | -8.3 | | 3 | Y | 5 | 0 | 1.38 | 320.3586432 | 49.85 |
| 24784208 | -8.3 | | 4 | Y | 7 | 2 | 2.54 | 360.45714 | 143.59 |
| 17387422 | -8.3 | | 3 | Y | 3 | 1 | 3.5 | 296.38674 | 66.76 |
| 24391503 | -8.3 | | 5 | Y | 7 | 2 | 1 | 360.45062 | 89.27 |
| 864166 | -8.3 | | 4 | Y | 7 | 0 | 2.41 | 353.41822 | 65.06 |
| 24368087 | -8.3 | | 4 | Y | 6 | 0 | 1.8 | 329.34716 | 76.82 |
| 24408934 | -8.3 | | 5 | Y | 7 | 1 | 2.73 | 337.37576 | 81.93 |
| 24290462 | -8.3 | | 2 | Y | 1 | 0 | 3.93 | 247.3111 | 11.3 |
| 144097781 | -8.3 | | 3 | Y | 5 | 0 | 3.28 | 360.4025832 | 71.79 |
| 49673905 | -8.3 | | 8 | Y | 6 | 2 | 3.04 | 413.53308 | 90.65 |
| 3717551 | -8.3 | | 4 | Y | 5 | 0 | 3.09 | 343.42332 | 112.77 |
| 7972186 | -8.3 | | 2 | Y | 5 | 0 | 3.44 | 285.38732 | 46.84 |
| 17416349 | -8.3 | | 4 | Y | 3 | 0 | 3.97 | 335.3979632 | 74.87 |
| 135383445 | -8.3 | | 2 | Y | 3 | 0 | 3.12 | 291.3405632 | 45.76 |
| 47202514 | -8.3 | | 3 | Y | 3 | 0 | 3.73 | 311.3501232 | 37.38 |
| 17517397 | -8.3 | | 6 | Y | 4 | 2 | 3.98 | 374.90428 | 58.2 |
| 103163614 | -8.3 | | 6 | Y | 7 | 1 | 2.82 | 438.56084 | 136.88 |
| 24817747 | -8.3 | | 6 | Y | 5 | 2 | 3.63 | 448.0212 | 85.25 |
| 85269963 | -8.3 | | 4 | Y | 6 | 2 | 2.83 | 317.3629 | 120.97 |
| 56324285 | -8.3 | | 8 | Y | 6 | 1 | 3.46 | 404.52302 | 101.01 |
| 4256259 | -8.3 | | 4 | Y | 6 | 0 | 2.69 | 345.38962 | 72.91 |
| 17443958 | -8.3 | | 2 | Y | 5 | 0 | 3.92 | 324.652961 | 71.64 |
| 24408066 | -8.3 | | 2 | Y | 4 | 0 | 3.24 | 305.37366 | 47.78 |
| 49674528 | -8.3 | | 3 | Y | 4 | 1 | 3.45 | 357.3474696 | 63.78 |
| 24289022 | -8.3 | | 0 | Y | 4 | 0 | 1.65 | 233.22488 | 55.74 |
| 4265825 | -8.3 | | 5 | Y | 6 | 0 | 2.62 | 380.50162 | 75.3 |
| 24298075 | -8.3 | | 2 | Y | 5 | 0 | 2.31 | 324.3737 | 55.04 |
| 24298127 | -8.3 | | 2 | Y | 6 | 2 | 1.75 | 281.3125 | 83.56 |
| 49718557 | -8.3 | | 7 | Y | 6 | 0 | 3.62 | 417.50018 | 64.43 |
| 14730517 | -8.3 | | 3 | Y | 5 | 1 | 3.72 | 365.44876 | 81.45 |
| 24333344 | -8.3 | | 6 | Y | 6 | 2 | 2.91 | 352.3838 | 76.99 |
| 7972392 | -8.3 | | 2 | Y | 5 | 1 | 1.84 | 281.35556 | 62.53 |
| 85198379 | -8.3 | | 6 | Y | 7 | 0 | 2.98 | 422.5383 | 92.37 |
| 49820106 | -8.3 | | 6 | Y | 6 | 3 | 1.92 | 367.4415 | 84.04 |
| 24368217 | -8.3 | | 4 | Y | 6 | 0 | 2.59 | 339.34198 | 68.98 |
| 3717849 | -8.3 | | 4 | Y | 6 | 3 | 1.97 | 330.37828 | 95.5 |
| 24305297 | -8.3 | | 5 | Y | 7 | 1 | 2.28 | 415.4380232 | 98.67 |
| 24293697 | -8.3 | | 1 | Y | 5 | 0 | 2.71 | 337.77808 | 72.06 |
| 4264250 | -8.3 | | 5 | Y | 5 | 1 | 3.56 | 326.36966 | 104.35 |
| 26614186 | -8.3 | | 4 | Y | 5 | 2 | 2.75 | 296.3669 | 62.45 |
| 3716109 | -8.3 | | 6 | Y | 7 | 0 | 3.88 | 427.49668 | 129.57 |
| 24296130 | -8.3 | | 2 | Y | 6 | 1 | 3.29 | 341.3843 | 102.41 |
| 4247562 | -8.3 | | 6 | Y | 6 | 1 | 3.52 | 408.49012 | 67.87 |
| 47197385 | -8.3 | | 6 | Y | 5 | 0 | 3.51 | 386.4597832 | 42.01 |
| 49721283 | -8.3 | | 8 | Y | 6 | 2 | 2.57 | 420.50082 | 74.94 |
| 103073761 | -8.3 | | 5 | Y | 4 | 1 | 3.43 | 338.42342 | 79.46 |
| 24782485 | -8.3 | | 4 | Y | 5 | 0 | 3.4 | 352.40694 | 86.49 |
| 7969210 | -8.3 | | 2 | Y | 4 | 0 | 2.87 | 327.44384 | 72.64 |
| 85269848 | -8.3 | | 8 | Y | 7 | 2 | 3.51 | 414.4947 | 93.73 |
| 24800652 | -8.3 | | 7 | Y | 6 | 0 | 3.57 | 399.4882 | 56.07 |
| 124949268 | -8.3 | | 6 | Y | 7 | 1 | 2.81 | 385.45678 | 72.92 |
| 24828449 | -8.3 | | 6 | Y | 5 | 1 | 1.91 | 350.41098 | 66.48 |
| 24792159 | -8.3 | | 5 | Y | 4 | 1 | 3.89 | 375.86912 | 81.86 |
| 4265874 | -8.3 | | 3 | Y | 6 | 1 | 3.16 | 306.31866 | 76.19 |
| 85199671 | -8.3 | | 5 | Y | 7 | 2 | 3.13 | 398.47868 | 129.31 |
| 24286742 | -8.3 | | 3 | Y | 4 | 0 | 2.63 | 264.27868 | 52.08 |
| 24370700 | -8.3 | | 2 | Y | 6 | 1 | 3.15 | 354.74394 | 85.19 |
| 4250701 | -8.3 | | 3 | Y | 7 | 1 | 3.02 | 322.31806 | 81.77 |
| 24809769 | -8.3 | | 2 | Y | 5 | 2 | 3.05 | 309.40872 | 62.53 |
| 865985 | -8.3 | | 6 | Y | 7 | 0 | 3.18 | 398.45224 | 83.56 |
| 22410920 | -8.3 | | 7 | Y | 7 | 2 | 2.99 | 436.90914 | 109.95 |
| 22406875 | -8.3 | | 9 | Y | 7 | 0 | 3.29 | 438.4746632 | 102.62 |
| 24398891 | -8.3 | | 4 | Y | 4 | 1 | 2.16 | 294.34772 | 49.41 |
| 49733902 | -8.3 | | 6 | Y | 7 | 1 | 1.59 | 389.46862 | 96.98 |
| 26660480 | -8.3 | | 3 | Y | 5 | 1 | 2.66 | 288.3200832 | 50.28 |
| 49675285 | -8.3 | | 5 | Y | 4 | 1 | 3.49 | 326.43264 | 56.04 |
| 26620360 | -8.3 | | 2 | Y | 4 | 0 | 3.56 | 344.4064 | 40.62 |
| 99454489 | -8.3 | | 7 | Y | 5 | 3 | 3.61 | 371.86058 | 74.81 |
| 24827972 | -8.3 | | 6 | Y | 7 | 2 | 1.72 | 342.34592 | 85.89 |
| 103074514 | -8.3 | | 4 | Y | 4 | 0 | 3.85 | 344.4031832 | 67.2 |
| 7971034 | -8.3 | | 3 | Y | 7 | 1 | 2.06 | 378.46912 | 131.43 |
| 26640007 | -8.3 | | 6 | Y | 5 | 2 | 2.7 | 364.43756 | 73.4 |
| 49647577 | -8.3 | | 6 | Y | 7 | 2 | 2.53 | 397.42112 | 112.58 |
| 26530189 | -8.3 | | 5 | Y | 5 | 1 | 2.4 | 341.3282296 | 62.3 |
| 85198999 | -8.3 | | 5 | Y | 7 | 2 | 2.45 | 395.2047 | 89.8 |
| 4251690 | -8.3 | | 6 | Y | 6 | 1 | 1.63 | 337.37246 | 73.22 |
| 17441816 | -8.3 | | 5 | Y | 5 | 1 | 3.05 | 372.48116 | 74.86 |
| 863179 | -8.3 | | 1 | Y | 5 | 0 | 2.98 | 283.32508 | 51.39 |
| 85268708 | -8.3 | | 5 | Y | 5 | 1 | 3.41 | 393.27494 | 68.29 |
| 49679945 | -8.3 | | 7 | Y | 6 | 1 | 3.06 | 404.52302 | 76.25 |
| 856213 | -8.3 | | 3 | Y | 5 | 3 | 2.79 | 325.40482 | 80.47 |
| 26636869 | -8.3 | | 6 | Y | 6 | 1 | 3.58 | 399.53294 | 126.24 |
| 99358349 | -8.3 | | 4 | Y | 4 | 0 | 2.21 | 279.29002 | 54.45 |
| 24375420 | -8.3 | | 3 | Y | 5 | 2 | 2.82 | 311.2591896 | 69.98 |
| 49665143 | -8.3 | | 3 | Y | 6 | 2 | 2.36 | 316.33178 | 123.23 |
| 4257206 | -8.3 | | 6 | Y | 7 | 3 | 2.12 | 402.49052 | 164.45 |
| 49737701 | -8.3 | | 6 | Y | 7 | 1 | 1.75 | 346.37768 | 82.82 |
| 24836957 | -8.3 | | 7 | Y | 7 | 1 | 2.72 | 403.43054 | 91.4 |
| 26647508 | -8.3 | | 4 | Y | 7 | 0 | 0.83 | 376.47316 | 76.05 |
| 847361 | -8.3 | | 5 | Y | 4 | 0 | 3.82 | 316.3500232 | 73.45 |
| 861349 | -8.3 | | 2 | Y | 4 | 0 | 2.54 | 314.42194 | 40.62 |
| 49737111 | -8.3 | | 6 | Y | 7 | 0 | 2.01 | 354.40298 | 73.39 |
| 4243629 | -8.3 | | 2 | Y | 4 | 0 | 3.06 | 323.38564 | 38.77 |
| 26539633 | -8.3 | | 4 | Y | 5 | 0 | 3.91 | 398.8083096 | 88 |
| 51088232 | -8.3 | | 4 | Y | 6 | 0 | 3.71 | 403.51996 | 47.5 |
| 92763989 | -8.3 | | 4 | Y | 5 | 2 | 1.91 | 307.34648 | 70.02 |
| 17411161 | -8.3 | | 8 | Y | 7 | 1 | 3.6 | 430.49732 | 128.27 |
| 110923162 | -8.3 | | 5 | Y | 6 | 0 | 2.97 | 347.36728 | 77.14 |
| 24344872 | -8.3 | | 3 | Y | 7 | 0 | 2.31 | 376.36214 | 89.71 |
| 17472624 | -8.3 | | 3 | Y | 4 | 1 | 2.95 | 338.44334 | 49.41 |
| 49665124 | -8.3 | | 6 | Y | 7 | 3 | 1.95 | 412.50526 | 128.98 |
| 90341274 | -8.3 | | 1 | Y | 4 | 1 | 3.11 | 283.2539032 | 51.58 |
| 14729396 | -8.3 | | 2 | Y | 5 | 0 | 1.94 | 330.3534632 | 53.76 |
| 49675309 | -8.3 | | 5 | Y | 5 | 0 | 3.57 | 390.5395 | 66.07 |
| 49723826 | -8.3 | | 8 | Y | 7 | 0 | 3.52 | 415.87008 | 77.69 |
| 24334068 | -8.3 | | 6 | Y | 5 | 1 | 3.88 | 396.4346832 | 85.75 |
| 7969256 | -8.3 | | 5 | Y | 5 | 0 | 3.23 | 368.47584 | 106.25 |
| 22412806 | -8.3 | | 2 | Y | 3 | 1 | 3.7 | 317.38106 | 42.23 |
| 125307020 | -8.3 | | 4 | Y | 5 | 1 | 2.97 | 357.42346 | 85.89 |
| 17464507 | -8.3 | | 2 | Y | 4 | 0 | 3.84 | 340.4393 | 78.51 |
| 24358782 | -8.3 | | 3 | Y | 7 | 2 | 0.93 | 350.38952 | 118.75 |
| 24818170 | -8.3 | | 3 | Y | 6 | 0 | 1.33 | 289.3081432 | 54.26 |
| 26534147 | -8.3 | | 5 | Y | 6 | 0 | 3.7 | 318.32936 | 80.8 |
| 24331648 | -8.3 | | 4 | Y | 5 | 0 | 3.29 | 334.39496 | 85.97 |
| 7965716 | -8.3 | | 6 | Y | 6 | 1 | 2.73 | 351.39904 | 69.29 |
| 24327399 | -8.3 | | 6 | Y | 6 | 1 | 2.5 | 370.44214 | 69.56 |
| 24336476 | -8.3 | | 3 | Y | 6 | 0 | 3.18 | 294.2616 | 88.92 |
| 865120 | -8.3 | | 5 | Y | 7 | 2 | 2.4 | 396.43636 | 92.78 |
| 24821644 | -8.3 | | 4 | Y | 3 | 1 | 3.38 | 327.3247064 | 42.23 |
| 57260751 | -8.3 | | 6 | Y | 7 | 0 | 3.37 | 407.50704 | 129.77 |
| 49673783 | -8.3 | | 6 | Y | 5 | 0 | 3.56 | 407.9309 | 48 |
| 26633305 | -8.3 | | 8 | Y | 7 | 1 | 3.11 | 416.4691432 | 103.57 |
| 17512468 | -8.3 | | 1 | Y | 5 | 4 | 3.06 | 284.33628 | 112.04 |
| 26530618 | -8.3 | | 4 | Y | 4 | 0 | 3.46 | 333.3077696 | 39.42 |
| 24805285 | -8.3 | | 0 | Y | 5 | 0 | 3.02 | 392.52894 | 53.99 |
| 49822777 | -8.3 | | 7 | Y | 6 | 1 | 3.37 | 410.48608 | 84.09 |
| 49819392 | -8.3 | | 7 | Y | 6 | 2 | 2.93 | 396.88834 | 96.45 |
| 49724106 | -8.3 | | 8 | Y | 7 | 1 | 2.71 | 409.52454 | 73.36 |
| 4258998 | -8.3 | | 5 | Y | 6 | 1 | 2.24 | 329.39352 | 65.64 |
| 24807074 | -8.3 | | 1 | Y | 4 | 0 | 3.79 | 276.28938 | 47.51 |
| 24363464 | -8.3 | | 4 | Y | 6 | 2 | 3.49 | 357.3621 | 84.08 |
| 26622174 | -8.3 | | 4 | Y | 5 | 1 | 3.73 | 371.4964 | 84.38 |
| 49648162 | -8.3 | | 2 | Y | 5 | 1 | 3.74 | 309.36236 | 59.91 |
| 57256402 | -8.3 | | 5 | Y | 7 | 1 | 2.07 | 415.5059 | 95.17 |
| 17463304 | -8.3 | | 2 | Y | 5 | 1 | 2.21 | 309.316 | 69.67 |
| 57261982 | -8.3 | | 5 | Y | 7 | 2 | 3.22 | 363.74916 | 94.09 |
| 14718862 | -8.3 | | 1 | Y | 3 | 1 | 4 | 319.39694 | 42.23 |
| 26725854 | -8.3 | | 1 | Y | 5 | 2 | 3.25 | 345.80338 | 103.09 |
| 74373762 | -8.3 | | 4 | Y | 6 | 0 | 3.7 | 436.93228 | 107.45 |
| 24834770 | -8.3 | | 5 | Y | 7 | 2 | 3.14 | 404.4170064 | 149.99 |
| 14719108 | -8.3 | | 2 | Y | 6 | 0 | 2.83 | 351.3311632 | 64.31 |
| 26650558 | -8.3 | | 5 | Y | 4 | 2 | 2.69 | 378.3881496 | 52.57 |
| 24369084 | -8.3 | | 3 | Y | 5 | 0 | 2.72 | 349.4063 | 82.92 |
| 24407871 | -8.3 | | 7 | Y | 6 | 1 | 3.59 | 423.4088496 | 93.13 |
| 22402844 | -8.3 | | 6 | Y | 7 | 1 | 2.81 | 430.49732 | 139.05 |
| 51085899 | -8.3 | | 5 | Y | 7 | 1 | 2.08 | 362.40836 | 110.37 |
| 24793601 | -8.3 | | 4 | Y | 5 | 1 | 2.25 | 334.2958296 | 59.29 |


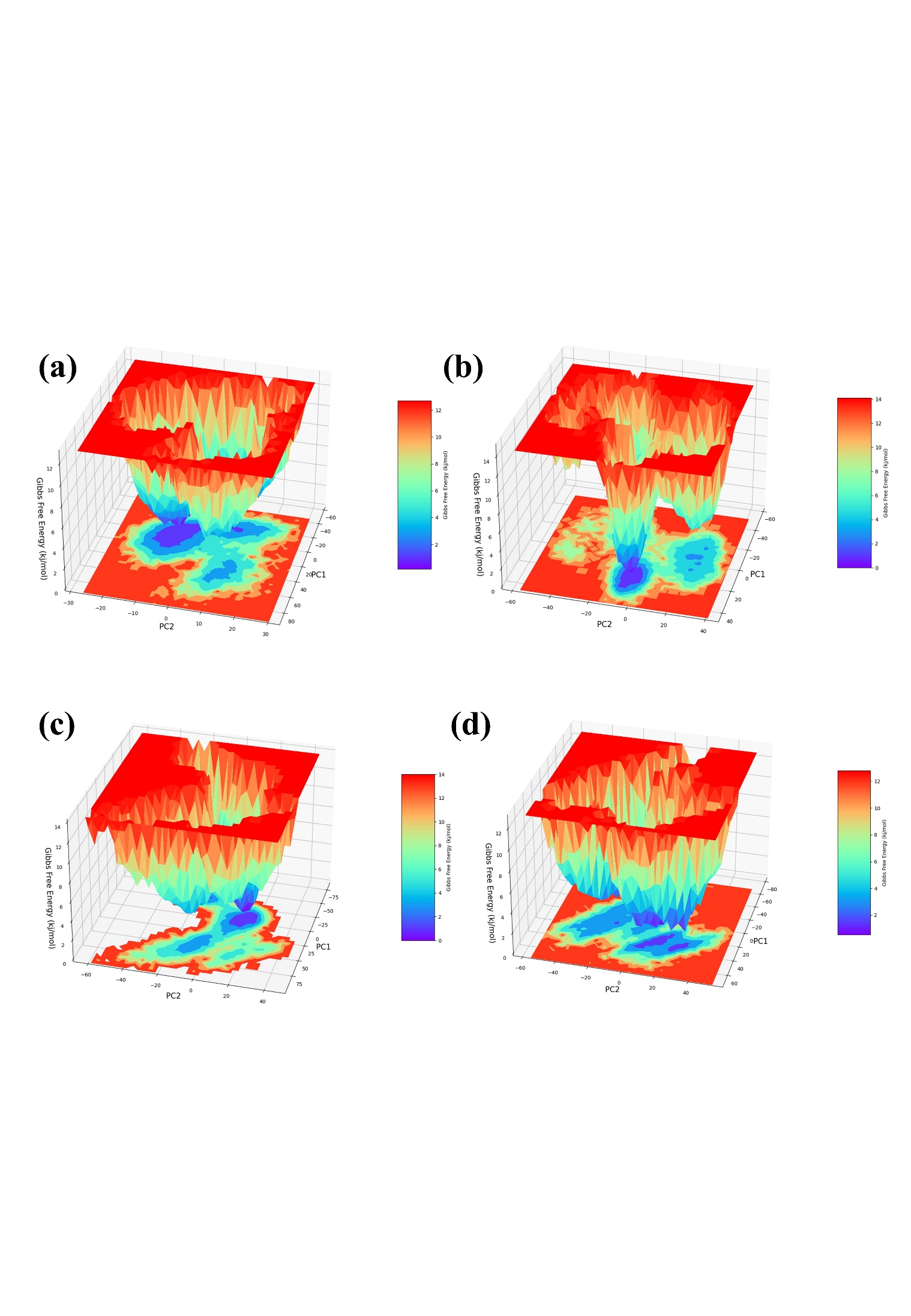
**Figure S1**: The 3D FEL plot of the HMPV nucleocapsid protein complexed with (a) 24330502 (b) 24292974 (c) 17515455 and (d) Gamma-Fagarine.
